# Building resilience against the growing threat of arboviruses II: a scoping review of *Aedes* vector competence, larval and adult bionomics and species distribution in Africa

**DOI:** 10.1186/s13071-026-07552-5

**Published:** 2026-07-28

**Authors:** Richard M. Oxborough, Basile Kamgang, Armel N. Tedjou, Tarekegn A. Abeku, Fatima Ahmed, Joseph R. Biggs, Kallista Chan, Jackie Cook, Amy Edwards, Basiliana Emidi, Jane Falconer, Louisa A. Messenger, Frederik Seelig, Roz Taylor, Aurelie P. Yougang, Jo Lines, Sian E. Clarke, Mojca Kristan

**Affiliations:** 1 Parasitology and Vector Biology (PARAVEC) Laboratory, School of Public Health, University of Nevada, Las Vegas, NV USA; 2 Department of Biological Sciences, College of Southern Nevada, Las Vegas, NV USA; 3 Centre for Research in Infectious Diseases (CRID), P.O. Box 13591, Yaoundé, Cameroon; 4 Malaria Consortium, London, UK; 5 Department of Disease Control, Faculty of Infectious and Tropical Diseases, London School of Hygiene and Tropical Medicine, London, WC1E 7HT UK; 6 Department of Infectious Disease Epidemiology and International Health, Faculty of Epidemiology and Population Health, London School of Hygiene and Tropical Medicine, London, WC1E 7HT UK; 7 National Institute for Medical Research, Dodoma Centre, P.O. Box 805, Dodoma, Tanzania; 8 Library, Archive and Open Research Services, London School of Hygiene and Tropical Medicine, London, WC1E 7HT UK; 9 Center for Immunology of Viral Infections (CiViA), Department of Biomedicine, Aarhus University, 8000 Aarhus, Denmark

**Keywords:** Vector competence, Bionomics, Species distribution, Arbovirus, *Aedes aegypti*, *Aedes albopictus*, Chikungunya, Dengue, Yellow fever

## Abstract

**Background:**

Reports of arboviral outbreaks are increasing, yet there are acknowledged shortfalls in surveillance data and outbreak preparedness on the African continent. The second part of this scoping review features *Aedes* vector–pathogen interactions, bionomics and species distribution.

**Methods:**

We reviewed studies published between 1980 and 2023 to synthesize existing information and identify knowledge gaps to guide future *Aedes* research and control in Africa.

**Results:**

877 full-text articles were reviewed, 70 featured vector–pathogen interactions, 230 vector bionomics and 394 species distribution. Vector competence studies were limited to a handful of countries, highlighting knowledge gaps regarding urban, bridge and sylvatic vector species competence for major arboviruses. *Aedes albopictus* was highly competent for chikungunya virus with the E1-226V mutation, which has been implicated in epidemics where *Ae. aegypti* was displaced. Vector competence was dependent on virus lineage and mosquito population. *Ae. aegypti* competence to dengue virus was highly variable, whereas *Ae. albopictus* was an efficient vector of Zika virus for African but not Asian lineages. Discarded tires were the most frequently reported positive breeding habitats, ranking among the top three in 55% of publications, followed by other discarded items (cans, bottles, broken plastic items) and water storage containers; however, the relative importance varied by location. The pupal index was reported less frequently than traditional indices (container, Breteau and house indices), which co-occurred in 84% of studies reporting indices. As pupal counts are a better proxy for adult mosquito production, their wider use is recommended to identify locally important breeding sites for targeted control. Adult vector monitoring has shifted away from human landing catch towards BG-Sentinel and CDC-LT traps which may be less accurate proxies of human-vector contact, but efficiency can be improved using attractants. *Ae. aegypti* was highly anthropophilic and had a major biting peak before sunset. However, findings of *Ae. aegypti* biting at night-time and being zoophilic in some locations demonstrate the need for periodic monitoring, given changing environmental conditions.

**Conclusions:**

This review highlights how *Aedes* populations vary in their competence for key arboviruses, their breeding habitats, and biting behaviors across Africa. Understanding these patterns can guide targeted vector control and improve preparedness for arbovirus outbreaks.

**Graphical Abstract:**

**Figure:**
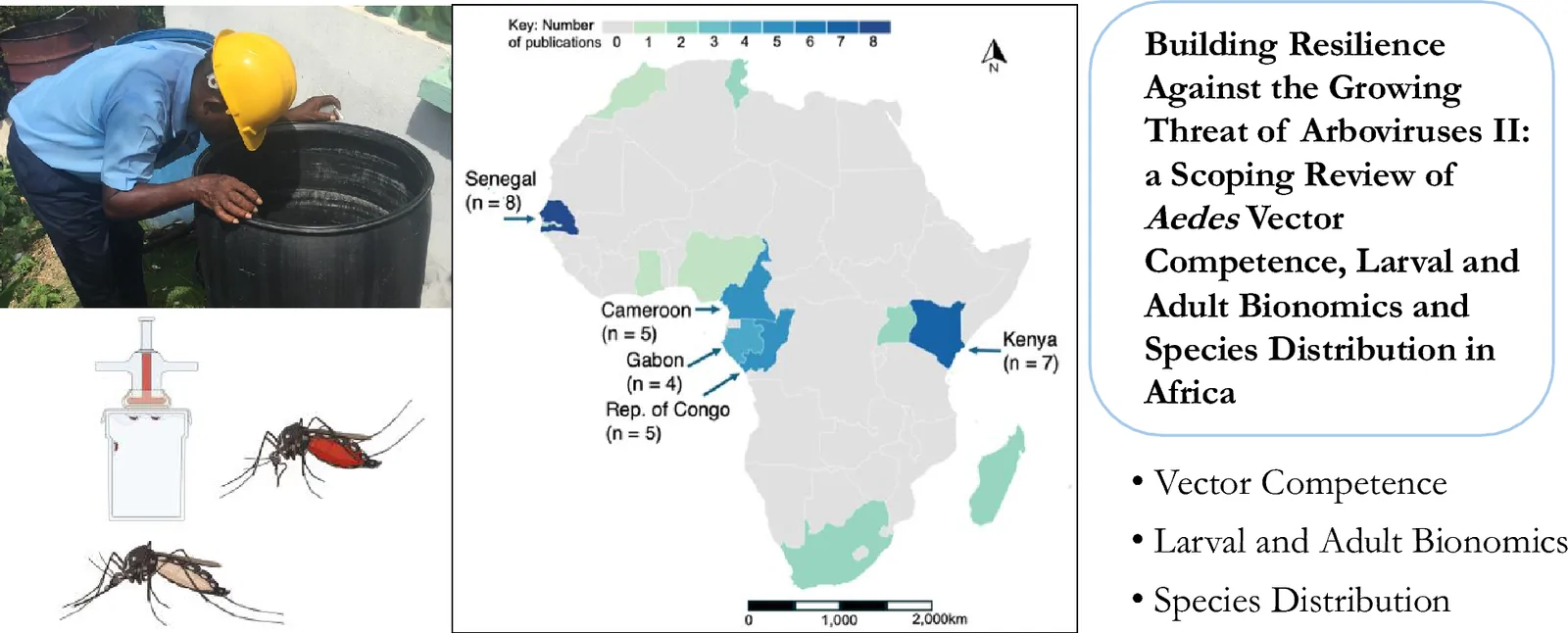

**Supplementary Information:**

The online version contains supplementary material available at https://doi.org/10.1186/s13071-026-07552-5.

## Background

Reports of arboviral outbreaks are increasing, yet there are acknowledged shortfalls in surveillance data and outbreak preparedness on the African continent [1, 2]. Several arboviruses of major public health importance in Africa—including yellow fever, dengue, chikungunya, and Zika—are transmitted by *Aedes* mosquitoes [3]. The recent years have seen unprecedented epidemics of dengue and chikungunya, both globally and within Africa [4], while yellow fever continues to kill between 31,000–82,000 people annually (with most deaths in Africa) [5]. However, the epidemiology and burden of arboviral diseases in Africa is insufficiently characterized. Control strategies are largely focused on malaria, and under-resourced healthcare constrains effective detection and management of arboviral infections [6]. Africa is also recognized as the origin of numerous arboviruses [7–9], as well as *Aedes aegypti*, which remains a primary vector worldwide [10, 11]. *Aedes aegypti* has been described as two subspecies: *Aedes aegypti aegypti* and *Aedes aegypti formosus,* which are genetically distinct and have different bionomic traits [12]. *Aedes aegypti formosus* is found mostly in sylvatic environments in sub-Saharan Africa, tends to be exophilic, and has a feeding preference for wild animals [13]. The second most important arboviral vector, *Ae. albopictus*, was introduced into mainland Africa from Asia in the early 1990s and has since spread across the continent [14, 15].

We conducted a scoping review of all literature published between 1980 and 2023 to summarize the state of knowledge of *Aedes* arbovirus vectors in Africa, specifically regarding vector–pathogen interactions, bionomics and vector species distribution. Vector–pathogen interactions includes vector competence, which describes the intrinsic ability of an arthropod vector to transmit an infectious agent [16], while bionomics is a broader term covering life processes, behavior and ecology of a vector species [17]. Such information is essential for understanding the factors that drive arbovirus transmission and for guiding evidence-based surveillance and vector–control strategies [18]. This scoping review complements our previous summary of *Aedes* surveillance, control strategies and insecticide resistance in Africa [19]. Together, they can be used to assist national disease control programs and other stakeholders to develop effective vector surveillance and control programs and guide future research to counter the growing outbreaks of arboviral diseases in Africa.

## Methods

The protocol for this scoping review is available through the Open Science Foundation (OSF) (https://doi.org/10.17605/OSF.IO/VZ5GH) [20], and results reported here according to PRISMA-ScR (Preferred Reporting Items for Systematic Reviews and Meta-Analyses Extension for Scoping Reviews) guidelines [21]. An initial exploratory review of a random subset of the available recent literature was conducted to develop a list of more detailed study questions (sub-questions) which were then categorized to produce two scoping reviews. Sub-questions for this second review focused on determining which *Aedes* species are primary and secondary vectors of each of the main *Aedes*-borne viral diseases, which are the most important breeding sites and how this varies by location, and the use of immature stage indices to predict outbreak risk. We also summarized the information available on host-seeking and resting adult *Aedes*, blood meal sources and biting times by species and location, and geographical species distribution, including the range and comparability of methods used in collecting these data. All maps were created using the web-based data visualization tool Datawrapper (Datawrapper GmbH, Berlin, Germany).

### Search strategy

Searches were conducted in 12 electronic databases that index published peer-reviewed and grey literature, using strings of terms, synonyms and controlled vocabulary terms to reflect three concepts described previously [19].The complete strings of search terms are published through the London School of Hygiene and Tropical Medicine (LSHTM) Data Compass digital repository (https://doi.org/10.17037/DATA.00004717) [22]. The bibliographic databases were identified based on their relevance to the topic following a consultation with an experienced librarian/information specialist who performed the searches. Databases were searched in January 2023, as described previously [19]. Search strategy verification was done by checking reference lists of key articles focusing on *Aedes* mosquitoes in Africa. Reference lists of these articles were additionally screened for potentially relevant citations missed by our electronic search. All references retrieved using the agreed search strategy were compiled, saved and de-duplicated in EndNote reference manager software, creating a review-specific database.

### Eligibility criteria and screening process

Journal articles published between January 1980 to January 2023 on studies conducted on the geographical continent of Africa (including French overseas departments of Mayotte and La Réunion) and published in any language, were eligible for inclusion. The 1980 cut-off was chosen pragmatically to capture the modern baseline prior to major ecological and epidemiological changes, including the later spread of *Ae. albopictus* and the expansion of urban arbovirus transmission. The EndNote database was exported to Rayyan systematic review software, allowing the review team to work together on the screening process, as described previously [19]. Articles were screened for relevance to one or more aspects of the two reviews in question, namely mosquito vector species being *Ae. aegypti*, *Ae. albopictus* or any of a list of 21 selected secondary *Aedes* vector species and topics of *Aedes* surveillance, vector control, insecticide resistance (review 1 [19]); vector–pathogen interactions, bionomics or distribution (review 2). Vector–pathogen interactions includes competence studies which involved laboratory infection studies using wild collected *Aedes* mosquitoes that were blood-fed with infectious blood spiked with characterized virus strains to determine parameters including dissemination of infection (viral dissemination in the wings, legs, head) and transmission rate (viral particles in the saliva). Competence studies were either conducted in the country where mosquitoes were collected or internationally after transfer of mosquito eggs overseas. Publications which involved screening wild *Aedes* mosquito populations for presence of DENV, CHIKV, YFV and ZIKV arbovirus infection were also included. We have reported mosquito species such as *Ae. bromeliae* (part of the *Ae. simpsoni* complex [23]) and subspecies of *Ae. aegypti formosus* and more urban-adapted *Ae. aegypti aegypti* as described in each publication. Subsequent studies of the subspecies have shown mixing of the two forms occurs and morphological features may not be reliable [24]. Articles were excluded if they did not include primary data, were conference proceedings or abstracts, were not journal articles, the full text could not be obtained, mosquito species or disease were not of interest, subject was not of interest (not related to any of the six topics of interest in the two reviews), data were not collected in Africa, or were collected pre-1980.

## Results

**Fig. 1:**
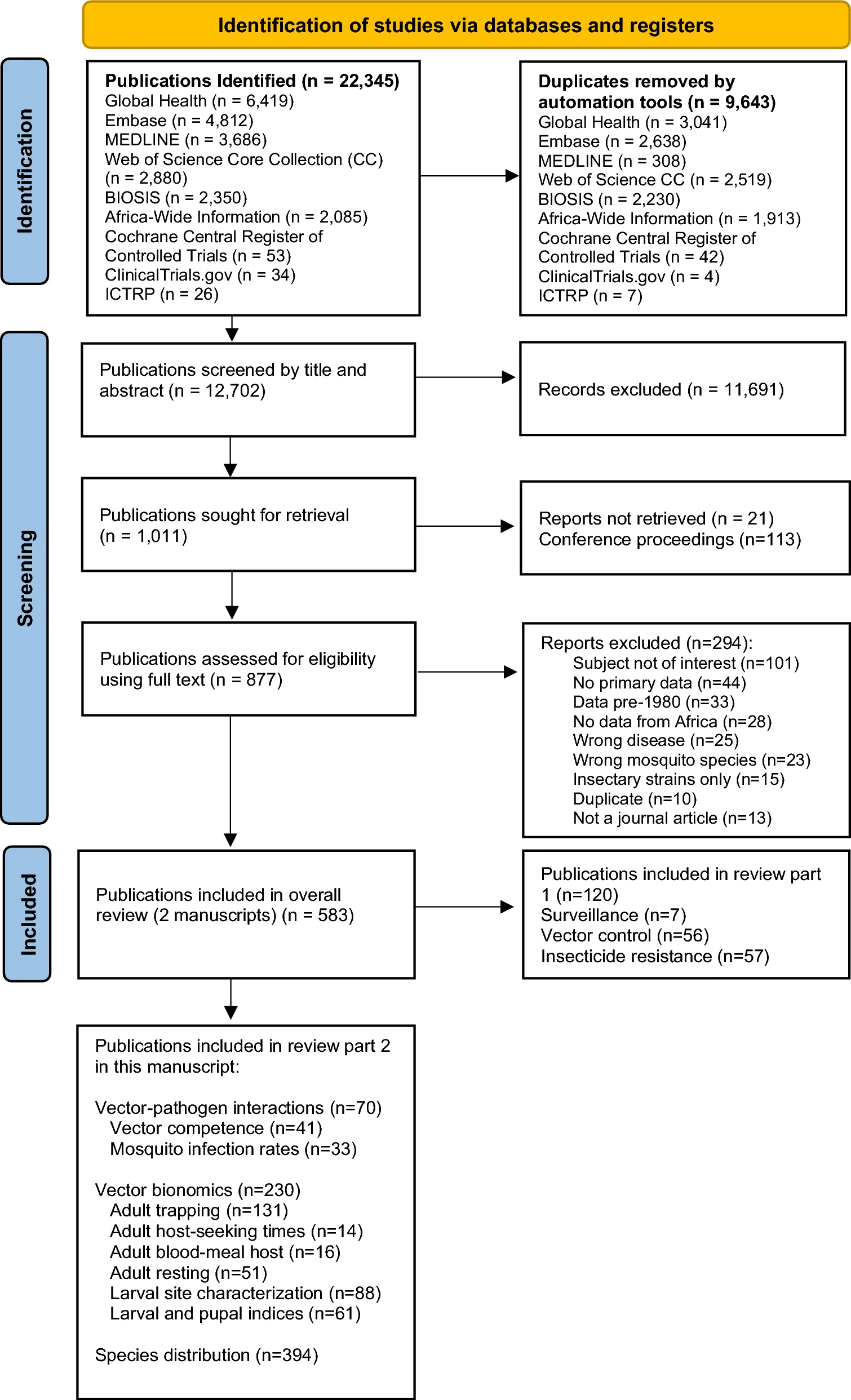
PRISMA flow chart illustrating the screening process for the scoping review, including sub-categorization of eligible publications containing vector competence, bionomics or species distribution data

Database keyword searches yielded a total of 22,345 articles published between 1980 and 2023, from which 12,702 publications were screened by title and abstract (Fig. 1). Of those, full texts were sought for 1011 publications of which 583 were eligible for inclusion in the overall review. This part of the scoping review examined three topics: vector–pathogen interactions, vector bionomics and species distribution. Of 462 eligible articles, 70 were included in the analyses of vector–pathogen interactions, 230 of vector bionomics and 394 of vector species distribution; categories were not mutually exclusive. Vector–pathogen interactions and bionomics were divided into sub-topics summarized in supplementary Table S1.1 vector competence (*n = *41), S1.2 mosquito infection rates (*n = *33), S2.1 adult mosquito trapping (*n = *131), S2.2 CO_2_ attractants (*n = *29), S2.3 host-seeking times (*n = *13), S2.4 blood–meal hosts (*n = *15), S2.5 adult mosquito resting (*n = *52), S2.6 larval and pupal indices (*n = *61), S2.7 breeding site characterization (*n = *88).

### Morphological identification of *Aedes* arbovirus vectors and distribution overview

A variety of resources were cited for morphological identification (> 28 articles cited), with the most cited keys summarized in Table 1. There were also locally relevant *Aedes* keys used for Madagascar [25], Sudan [26], and Cabo Verde [27]. Molecular methods were not routinely used for species identification but were utilized for specific studies (beyond the scope of this review) to distinguish cryptic species such as *Ae. furcifer* and *Ae. taylori* [28] and explore genetic differences between the subspecies *Ae. aegypti aegypti* and *Ae. aegypti formosus* [29].

**Table 1:** Most frequently cited references for morphological identification

Publication year	Authors	Title	Times cited	References
1941	Edwards, FW	Mosquitoes of the Ethiopian region. III: Culicidae adults and pupae	80	[30]
1952	Hopkins, G	Mosquitoes of the Ethiopian region, I: larval bionomics of mosquitoes and taxonomy of Culicine larvae	18	[31]
1972	Gillett, JD	Common African Mosquitoes and their medical importance	19	[32]
1996	Jupp, PG	Mosquitoes of Southern Africa: Culicinae and Toxorhynchitinae	29	[33]
2004	Rueda, LM	Pictorial keys for the identification of mosquitoes (Diptera:Culicidae) associated with dengue virus transmission	14	[34]
2004	Huang, Y	The subgenus *Stegomyia* of *Aedes* in the Afrotropical Region with keys to the species (Diptera: Culicidae)	33	[35]

There were 394 publications that featured *Aedes* species distribution data, with *Ae. aegypti* reported in 37 countries and *Ae. albopictus* in 23 countries from 97 publications. *Aedes vittatus* was the most widely reported secondary vector species (20 countries), followed by sylvatic or bridge species *Ae. africanus*, *Ae. luteocephalus*, *Ae. metallicus* and *Ae. fowleri* (Tables 2).

**Table 2:** Ranking of *Aedes* species by number of countries present between 1980–2023 (denominator of 54 African countries plus overseas territories)

Mosquito species	Number of countries	Number of publications
*Ae. aegypti*	37	302
*Ae. albopictus*	23	97
*Ae. vittatus*	20	61
*Ae. africanus*	15	52
*Ae. luteocephalus*	11	52
*Ae. metallicus*	10	23
*Ae. fowleri*	10	13
*Ae. furcifer*	7	37
*Ae. hirsutus*	5	11
*Ae. bromeliae*	5	8
*Ae. taylori*	4	24
*Ae. unilineatus*	4	9
*Ae. dalzielii*	3	16
*Ae. minutus*	1	3
*Ae. neoafricanus*	1	2

### *Aedes* vector–pathogen interactions

#### Laboratory studies of vector competence

**Fig. 2:**
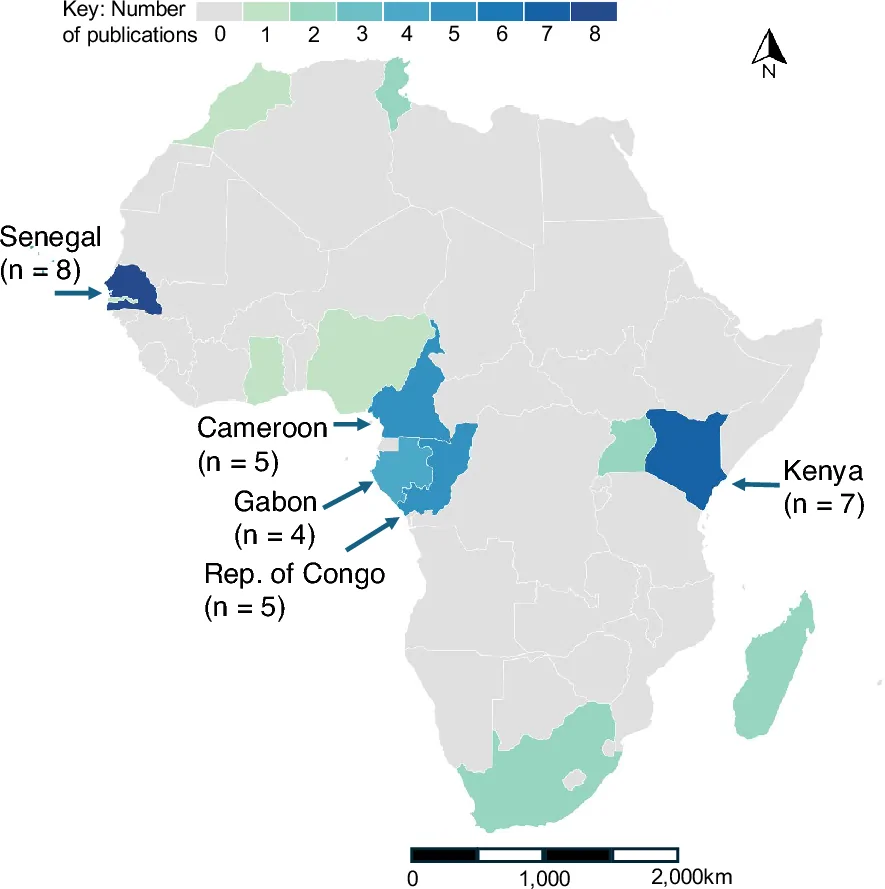
Number and geographical distribution of publications on *Aedes* vector competence since 1980, (small islands not visible, Cabo Verde *n = *4, Mayotte *n = *2, La Réunion *n = *8)

*Aedes* vector competence data (laboratory studies measuring viral infection, dissemination, and transmission using wild-collected *Aedes* that were blood-fed with infectious blood spiked with characterized virus strains) were featured in 41 publications from 14/54 (26%) countries in Africa plus the territories of La Réunion and Mayotte, as shown in Fig. 2. Vector competence studies were conducted in few countries, with Senegal (*n = *8), La Réunion (*n = *8), Kenya (*n = *7), Republic of Congo (*n = *5), Cameroon (*n = *5) and Gabon (*n = *4) being the primary locations; all of which reported major arbovirus epidemics prior to vector competence studies. There was a notable contribution from researchers linked to Institut Pasteur: featuring in half (21/41) of the studies, including publications from Gabon, Tunisia, Morocco, Cameroon, Republic of Congo, Madagascar, La Réunion and Cabo Verde (Table 3).

**Table 3:** Summary of arbovirus vector competence studies conducted in Africa with *Ae. aegypti*, *Ae. aegypti formosus*, *Ae. albopictus* and secondary vector species

Mosquito species	DENV	CHIKV	YFV	ZIKV	Countries
*Ae. aegypti*	12	5	4	2	Cabo Verde [39, 44], Cameroon [40, 43, 45], Gabon, Kenya [42, 46–49], Madagascar [50], Mayotte [41], Senegal[36–38, 51–53], South Africa [54, 55]
*Ae. aegypti formosus*	4	2	2	1	Cabo Verde [56], Gabon [57, 58], Ghana[59], Madagascar [60], Nigeria [61], Uganda [62]
*Ae. albopictus*	6	8	2	5	Cameroon [43, 45, 63], Gabon [57], La Réunion [41, 64–67], Mayotte, Morocco[68], Rep of Congo [69], Tunisia [70, 71], Misc. [72]
*Ae. bromeliae*	0	1	0	0	Kenya [73]
*Ae. cordellieri*	0	0	1	0	South Africa [55]
*Ae. furcifer*	2	1	1	0	Senegal[37, 74], South Africa [54, 55]
*Ae. luteocephalus*	2	0	0	0	Senegal [37, 74]
*Ae. simpsoni* s.l	0	0	2	0	Kenya[49], South Africa [55]
*Ae. taylori*	1	0	0	0	Senegal [37]
*Ae. vittatus*	1	1	0	0	Kenya[73], Senegal [74]
Total	28	18	12	8	

Table 3 provides an overview of the vector competence studies conducted by mosquito species, virus and country. A substantial proportion of studies focused on the competence of *Ae. aegypti* and invasive *Ae. albopictus* for dengue virus (DENV) and chikungunya virus (CHIKV). Studies of sylvatic and bridge vector species, other than *Ae. aegypti formosus*, were limited to Senegal, Kenya and South Africa. A simplified summary of vector competence is shown in Table 4. In general, *Ae. aegypti* in Africa had lower vector competence for dengue than more efficient mosquito populations from other regions (e.g. Asia). In Senegal, both sylvatic and urban *Ae. aegypti* had low vector competence nationwide for DENV-1, 3 and 4 [36–38]. In Cabo Verde, *Ae. aegypti* was highly competent for DENV-2 and 3 strains but had low susceptibility for DENV-1 and 4 [39]. Studies in Cameroon demonstrated high geographic variability in competence for dengue [40]. *Aedes aegypti* was a competent CHIKV vector in several cities in Kenya as well as Mayotte [41, 42]. In Cabo Verde, Cameroon and Gabon *Ae. aegypti* were potentially effective ZIKV vectors [43, 44].

**Table 4:** Simplified summary of vector competence of *Aedes* species in Africa for chikungunya (CHIKV), dengue (DENV), yellow fever (YFV) and Zika (ZIKV) viruses

Vector species	DENV	CHIKV	YFV	ZIKV
*Ae. aegypti*	Highly variable competence depending on DENV strain and mosquito origin [36–38, 40]	Highly efficient primary vector, similar competence as *Ae. albopictus *[41, 42]	Variable competence by location [45, 55, 75]	Potentially effective ZIKV vector in several countries [43, 44]
*Ae. aegypti formosus*	Low efficiency or refractory [56, 58–60]	Less efficient vector than *Ae. albopictus* and *Ae. aegypti* [56, 57]	Low efficiency but implicated as primary vector in Nigeria outbreak [56, 61]	Competent vector of African lineage ZIKV [62]
*Ae. albopictus*	Competent vector but to a lesser degree than for CHIKV [57, 67, 68, 71]	Highly efficient primary vector of E1-226 V mutated CHIKV in several parts of Africa [41, 64, 68, 70]	Low transmission potential [45, 68]	Efficient vector of African virus lineages, but strong midgut barrier to Asian strains [43, 71]
Other species	–	*Ae. vittatus* highly competent vector, *Ae. furcifer* and *Ae. bromeliae* potential vectors [51, 73]	–	–

*Ae. aegypti formosus* (Aaf) from Ghana were refractory to DENV-2 (isolated from Ghana), while samples from Gabon, Madagascar, Senegal and Cabo Verde exhibited lower vector competence for DENV-2 than comparison populations of *Ae. aegypti* [56, 58–60] (Table S1.1). In Gabon Aaf was a less competent CHIKV vector than *Ae. albopictus* [57]. Aaf in Nigeria were relatively resistant to YFV infection but were believed to be the primary vector of an urban epidemic in Oyo State [61]. However, in Uganda, Aaf were competent vectors for a local lineage of ZIKV [62] and in Cabo Verde had high potential to transmit CHIKV and YFV [56].

Six studies showed that *Ae. albopictus* from Gabon, La Réunion, Morocco and Tunisia was a competent vector for DENV (mostly using an Asian isolate of DENV-2 from Bangkok, 1974) but exhibited lower transmission efficiency than with CHIKV [57, 67, 68, 71]. *Aedes albopictus* is a highly competent primary vector of CHIKV in parts of Africa (Table 5), equally competent as *Ae. aegypti* [63, 64], and more efficient than *Ae. aegypti formosus* [57]. *Aedes albopictus* from La Réunion [64], Mayotte [41], Gabon [57], Cameroon [63], Tunisia [70] and Morocco [68] were particularly susceptible to the 06.21 strain, isolated during the outbreak on La Réunion in 2005/06 [65]. This strain carries a point mutation, which changed the primary structure of the E1 envelope glycoprotein and increased vector competence of *Ae. albopictus,* which is purported to explain the expansion of CHIKV transmission [76]. An exception was in the Republic of Congo, where *Ae. albopictus* was less able than *Ae. aegypti* to transmit a CHIKV strain (Congo_2011) which harbors the same E1-226V mutation. *Aedes albopictus* from the Republic of Congo were found to be genetically different to those from La Réunion, where the *Ae. albopictus*-adaptive substitution E1-A226V has been selected [69]. Five vector competence studies were conducted with ZIKV and showed that virus lineage was an important factor, with African *Ae. albopictus* populations having a strong midgut barrier to the Asian ZIKV genotypes [71] but being more competent with African lineages [43]. *Aedes albopictus* from Tunisia and Central Africa were capable of transmitting YFV, but the virus was only detected in mosquito saliva 14–21 days post-infection (compared to 3 days for CHIKV), suggesting lower transmission potential [45, 68].

**Table 5:** Summary of *Aedes albopictus* vector competence studies for CHIKV

Country (Reference)	Year of study	Strain of CHIKV	Key findings
La Réunion and Mayotte [64]	2006	05.115, La Réunion 06.21, La Réunion 06.111, Mayotte 06.117, DR Congo	Dissemination rates > 2 times higher with 06.21 than 05.115 strain. *Ae. albopictus* from La Réunion equally competent as *Ae. aegypti* from Vietnam
La Réunion [65]	2006	06.21, La Réunion	*Ae. albopictus* was co-infected and disseminated CHIKV and DENV
Gabon [57]	2007	06.21, La Réunion	*Ae. albopictus* more efficient than *Ae. aegypti formosus*
Cameroon [63]	2007	06.21, La Réunion	*Ae. aegypti* and *Ae. albopictus* from Cameroon both highly competent vectors
La Réunion and Mayotte [41]	Nd	E1-226A, La Réunion E1-226V, La Réunion	*Ae. albopictus* more susceptible to E1-226V than E1-226A. This may explain the predominance of E1-226V where *Ae. albopictus* is the main vector
Republic of Congo and La Réunion [69]	2015	DRC_2000, DR Congo Congo_2011, DR Congo 06.21, La Réunion 05.115, La Réunion	*Ae. albopictus* less able than *Ae. aegypti* from Rep. of Congo to transmit the most recent epidemic CHIKV strain, Congo_2011, which harbors the E1-226V mutation
Morocco [68]	2016	06.21, La Réunion	*Ae. albopictus* highly capable of transmitting CHIKV and virus detectable 3 days post-exposure
Tunisia [70]	2017–18	06.21, La Réunion	*Ae. albopictus* from Tunisia were as competent as those from La Réunion

*Nd* not described

A limited number of competence studies conducted with sylvatic or bridge species demonstrated that *Ae. vittatus* is a competent vector of CHIKV in Senegal and Kenya, while *Ae. furcifer* and *Ae. bromeliae* are potential CHIKV vectors [51, 73] with *Ae. furcifer* and *Ae. luteocephalus* competent vectors of DENV in Senegal [74].

#### Field surveys—detection of arbovirus infections in wild *Aedes* populations

**Fig. 3:**
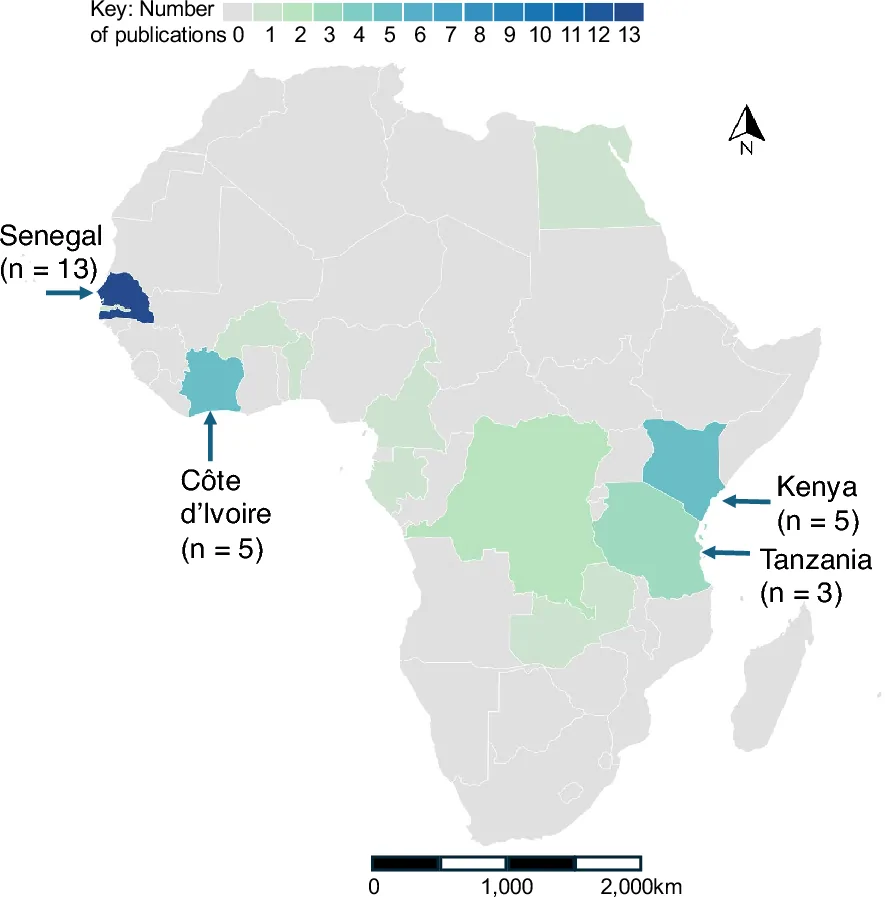
Number and geographical distribution of publications on arbovirus infection in wild-collected *Aedes* since 1980, by country (small islands not visible, Comoros *n = *1)

There were 33 publications which involved screening wild *Aedes* mosquito populations for presence of arboviruses (DENV, CHIKV, YFV and ZIKV), conducted in 12/54 (22%) countries with Senegal (13), Kenya (5) and Côte d’Ivoire (5) having the most published studies (Fig. 3; Table S1.2). Mosquitoes were pooled together and tested for the presence of virus. Maximum pool size varied by study from 10 to 100 individuals and was described to varying degrees of detail. Most studies reported only the number of positive pools and infection prevalence calculations were infrequently reported and used different approaches. Minimum infection rate (MIR) (total positive pools ÷ total mosquitoes tested × 1000) gives a simple metric per 1000 mosquitoes. MIR values reported were 0.2 for *Ae. aegypti* with DENV in Kenya [77], and 4.0 for CHIKV in Comoros [78]. More complex maximum likelihood modeling estimates were 8.49 for *Ae. aegypti* DENV prevalence in Tanzania [79] and 8.6 for *Ae. aegypti formosus* CHIKV prevalence in Senegal [80]. It is notable that many arbovirus detection studies were done during outbreak investigations, including five dengue [63, 77, 79, 81, 82], three chikungunya [78, 83, 84] and three yellow fever epidemics [85–87]. *Aedes aegypti* tested positive for CHIKV in four countries, DENV-2 in three countries and DENV-3 in two countries, ZIKV in Senegal and YFV in Senegal and Côte d’Ivoire (Table 6). There were four studies of *Ae. aegypti* in Tanzania, Kenya and Côte d’Ivoire that resulted in no virus detection, three of which had moderate or large sample sizes up to several thousand mosquitoes analyzed [88–90]. This demonstrates that arbovirus circulation is highly variable over time and that large sample sizes are often required to detect virus.

**Table 6:** Summary of *Aedes* arbovirus prevalence studies that detected positive virus pools

Mosquito species	Country and year of detection
Dengue	
*Ae. aegypti*	Côte d’Ivoire (nd, DENV-3) [81], Kenya (2013–14, DENV-2) [77], Tanzania (2014, DENV-2) [79], Kenya (2014–16, nd) [92], Egypt (2017, DENV-2) [82], Benin (2020–21, DENV-3) [93]
*Ae. albopictus*	Gabon (2007, DENV-2) [63]
*Ae. furcifer*, *Ae. taylori*, *Ae. luteocephalus*	Senegal (1981–82, 1988–91, DENV-2) [94, 95]
Chikungunya	
*Ae. aegypti*	Senegal (1996–97) [84], Comoros (2005) [78], Kenya (2014–16) [92], DR Congo (2019) [83]
*Ae. aegypti formosus*	Senegal (2015) [80]
*Ae. albopictus*	Gabon (2007) [63], DR Congo (2019) [83, 91]
*Ae. furcifer*, *Ae. luteocephalus*	Senegal (1988–1991, 2015) [80, 95]
*Ae. dalzieli*, *Ae. taylori*, *Ae. vittatus*	Senegal (1988–1991) [95]
*Ae. minutus*	Senegal (2015) [80]
Yellow fever	
*Ae. aegypti*	Senegal (1996–97, 2003–4) [84, 85, 96], Côte d’Ivoire (2008) [86]
*Ae. furcifer*	Senegal (1981–82, 1993–94, 2002, 2003–4, 2010, 2015) [80, 87, 95–98], Côte d’Ivoire (1999) [99]
*Ae. taylori*, *Ae. luteocephalus*	Senegal (1981–82, 1993–94, 2010, 2015) [80, 95, 97, 98]
*Ae. africanus*, *Ae. vittatus*	Senegal (2010, 2015–17) [80, 98]
*Ae. centropunctatus*, *Ae. dalzieli*, *Ae. mcintoshi*	Senegal (2010) [98]
Zika	
*Ae. aegypti*, *Ae. vittatus*	Senegal (1981–82, 2011) [95, 100]
*Ae. furcifer*, *Ae. luteocephalus*	Senegal (1981–82, 1993–94, 2011, 2015) [80, 95, 97, 100]
*Ae. taylori*, *Ae. dalzieli*	Senegal (1981–82, 2011, 2015) [80, 95, 100]
*Ae. neoafricanus, Ae. fowleri, Ae. minutus*	Senegal (1981–82) [95]
*Ae. africanus, Ae. hirsutus*, *Ae. metallicus*	Senegal (2011) [100]
*Ae. bromeliae*, *Ae. unileatus*	Senegal (2015–17) [101]

*nd* not described

*Aedes aegypti formosus* were found to be infected with CHIKV in Senegal in both the forest canopy and within a village [80]. *Aedes albopictus* tested positive for CHIKV in Gabon [63] and DR Congo [83, 91] and was implicated as the primary vector during outbreaks in parts of Central Africa where *Ae. aegypti* had been displaced. *Aedes albopictus* only tested positive for DENV in one study in Gabon [63]. Several sylvatic or bridge vector species were positive for either CHIKV, DENV, YFV or ZIKV in Senegal and Côte d’Ivoire. Three species (*Ae. furcifer*, *Ae. luteocephalus* and *Ae. taylori*) were positive in studies in Senegal for all four arboviruses (Table 6).

### *Aedes* bionomics

**Fig. 4:**
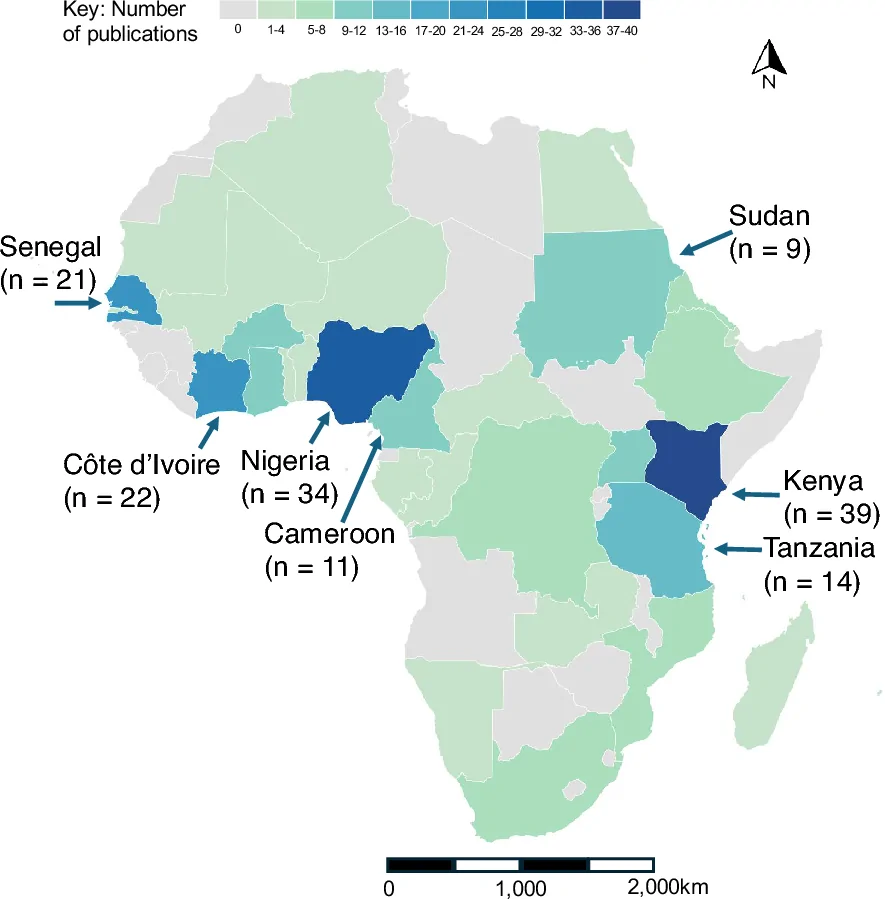
Number and geographical distribution of publications on *Aedes* arbovirus vector bionomics since 1980, by country. Total publications = 230 (small islands not visible, Cabo Verde *n = *3, Comoros *n = *2, Mauritius *n = *1, Mayotte *n = *1, La Réunion *n = *8)

Bionomics is the study of vector biology, behavior and ecology in relation to how they survive and transmit pathogens [17]. Studies concerning *Aedes* vector bionomics were quite numerous, with a total of 230 publications from 35 countries, of which 13 countries, including the territory of La Réunion, produced more than four publications (Fig. 4).

#### Larval bionomics—characterization and productivity of breeding sites

**Fig. 5:**
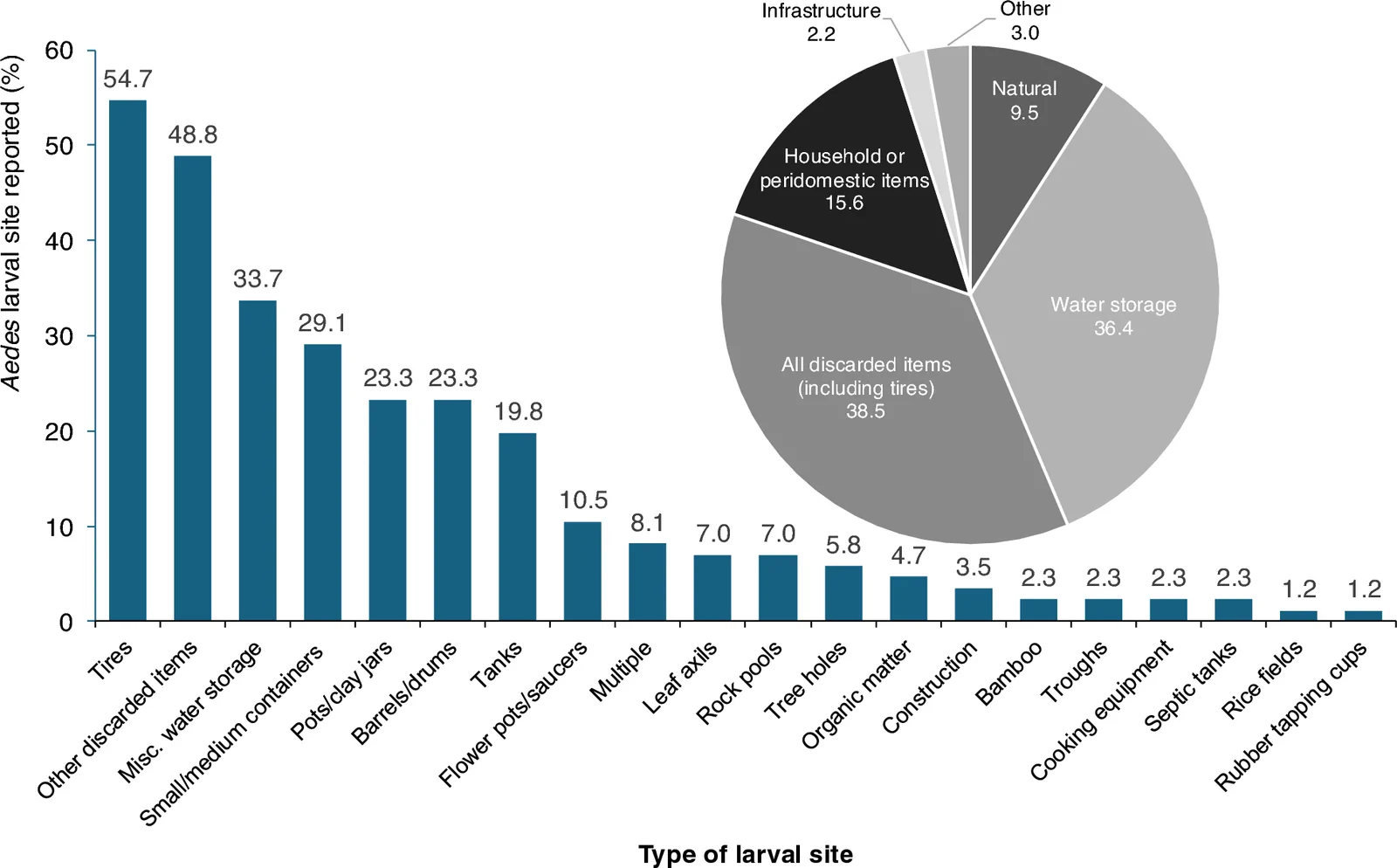
Most frequently reported categories of positive *Aedes* larval habitat (% of studies reporting each among the top three positive sites)

Surveys of *Aedes* breeding sites were reported in 150 publications, with information on the environment and characteristics of positive sites reported in 86/150 (57%). Most studies were conducted relatively recently, with 65/86 (76%) since 2010. The type of breeding sites varied substantially, but could be grouped into six broad categories, with the most important being discarded items (tires, cans, bottles, broken plastic items, broken water storage vessels), water storage containers (barrels/drums, pots/clay jars, tanks, troughs, jerry cans), household or peridomestic items (portable usable plastic containers, cooking equipment, flower pots) and natural larval sites (tree holes, leaf axils, bamboo, rock pools, coconut shell, rice field, latex rubber tapping cup) (Fig. 5). Discarded tires were the most frequently reported positive larval site (featuring in the top three larval sites in 54.7% of all publications), followed by other discarded items (48.8%) and several types of water storage containers (misc. water storage (33.7%), pots/clay jars (23.3%), barrels/drums (23.3%), tanks (19.8%)) (Fig. 5). Mombasa city, Kenya (the setting of several dengue epidemics) is a typical urban setting where jerry cans for water storage were the most abundant container searched, but discarded tires had the highest number positive for *Ae. aegypti* larvae/pupae, followed by drums, plastic buckets and jerry cans [77]. In Burkina Faso a comparison of three locations near Ouagadougou (a site of significant dengue epidemics) determined significant differences based on urbanicity. Car tires were a common *Ae. aegypti* breeding site in urban and peri-urban areas, but far less common in a rural setting, where animal drinking troughs were the most productive, while infested large water storage containers were most common in areas without piped water [17].

The variability by location (presumably based on sociocultural, economic factors and ecology) was particularly noteworthy, with some less common larval sites being of significant local importance. For example, in rural villages of South Omo Zone, Ethiopia, leaf axils of the false banana (*Ensete ventricosum*) and banana (*Musa* spp.) were the two leading positive larval habitats [102]. The type of water storage containers also varied depending on cultural context, with clay pots/jars (known locally as zeers) common positive containers in Sudan [103–105] and in parts of West Africa (known as *canaris*) [106, 107], while tanks [108], barrels [109], jerry cans [110], and buckets [111] were used and positive for immature stages elsewhere. Studies in Côte d’Ivoire indicated that land use changes greatly influenced the presence of *Aedes* and the relative importance of containers, with tree holes and bamboo common larval habitats in rainforest, latex collection cups in rubber plantations whilst water storage and discarded containers were important in villages [112, 113].

**Fig. 6:**
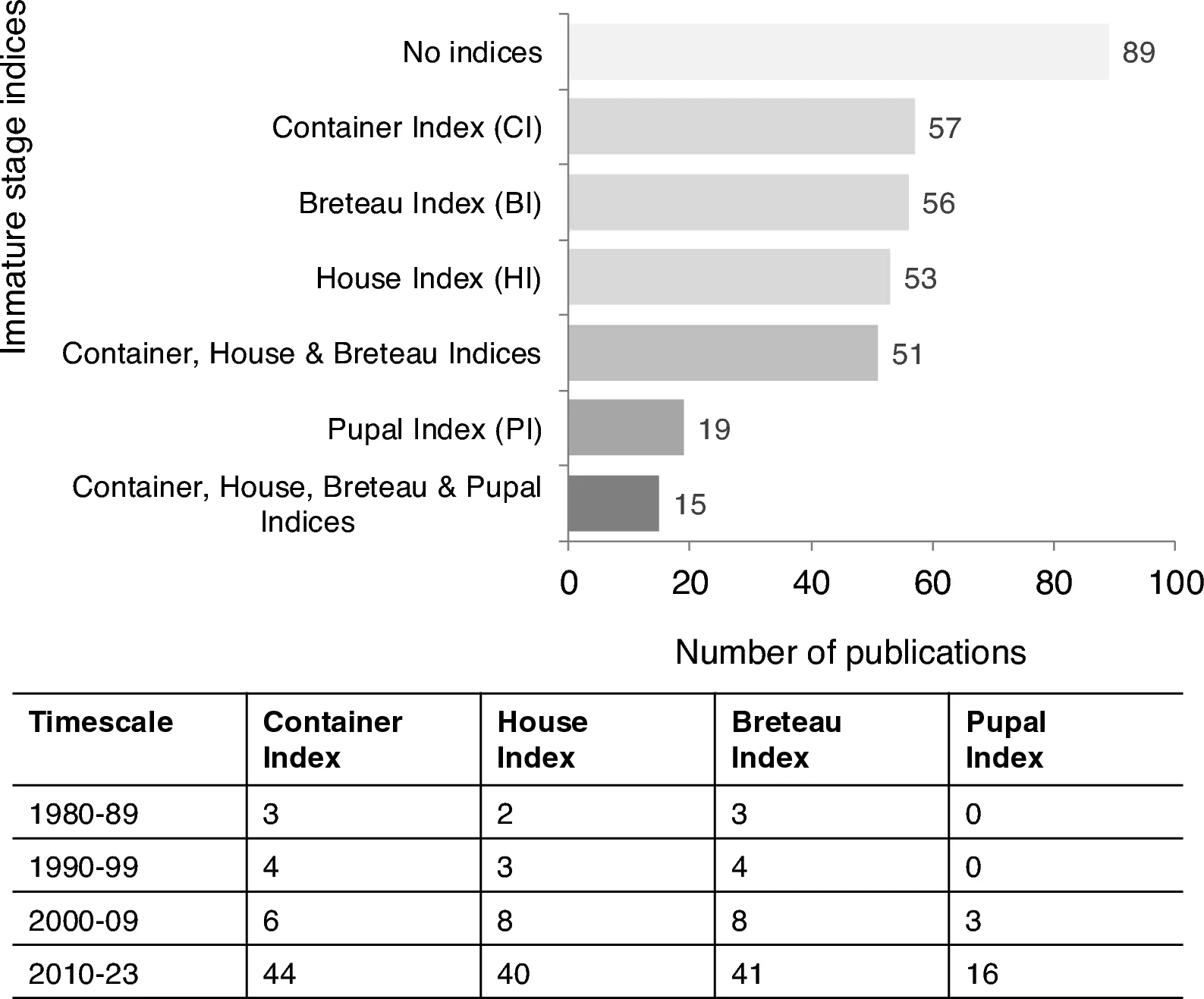
Type of *Aedes* indices reported in publications that included surveys of immature stages, and change in indices reported over ten year time intervals from 1980–2023

Larval or pupal indices were reported as a measure of vector infestation, which serve as proxies for arbovirus transmission risk, in 41% of breeding site studies (61/150) (Fig. 6, Table S2.6). The remaining 59% of studies tended to only report species composition, abundance and breeding site characteristics. Container index, house index and Breteau index were frequently reported together (51/61) in 84% of publications that reported immature stage indices, while the pupal index only featured in 31% (19/61). Most publications featuring indices featured data collected between 2010–23, with few in the 1980s and 1990s (Fig. 6).

Key: House index (HI) – Percentage of houses (including indoor and outdoor spaces) positive for *Aedes*. Container index (CI) – Percentage of water containers positive for *Aedes*. Breteau index (BI) – Number of positive containers per 100 houses inspected. Pupal index (PI)—Number of *Aedes* pupae per 100 houses inspected [114].

#### Adult *Aedes* bionomics; host-seeking and resting, including trapping methods used

**Fig. 7:**
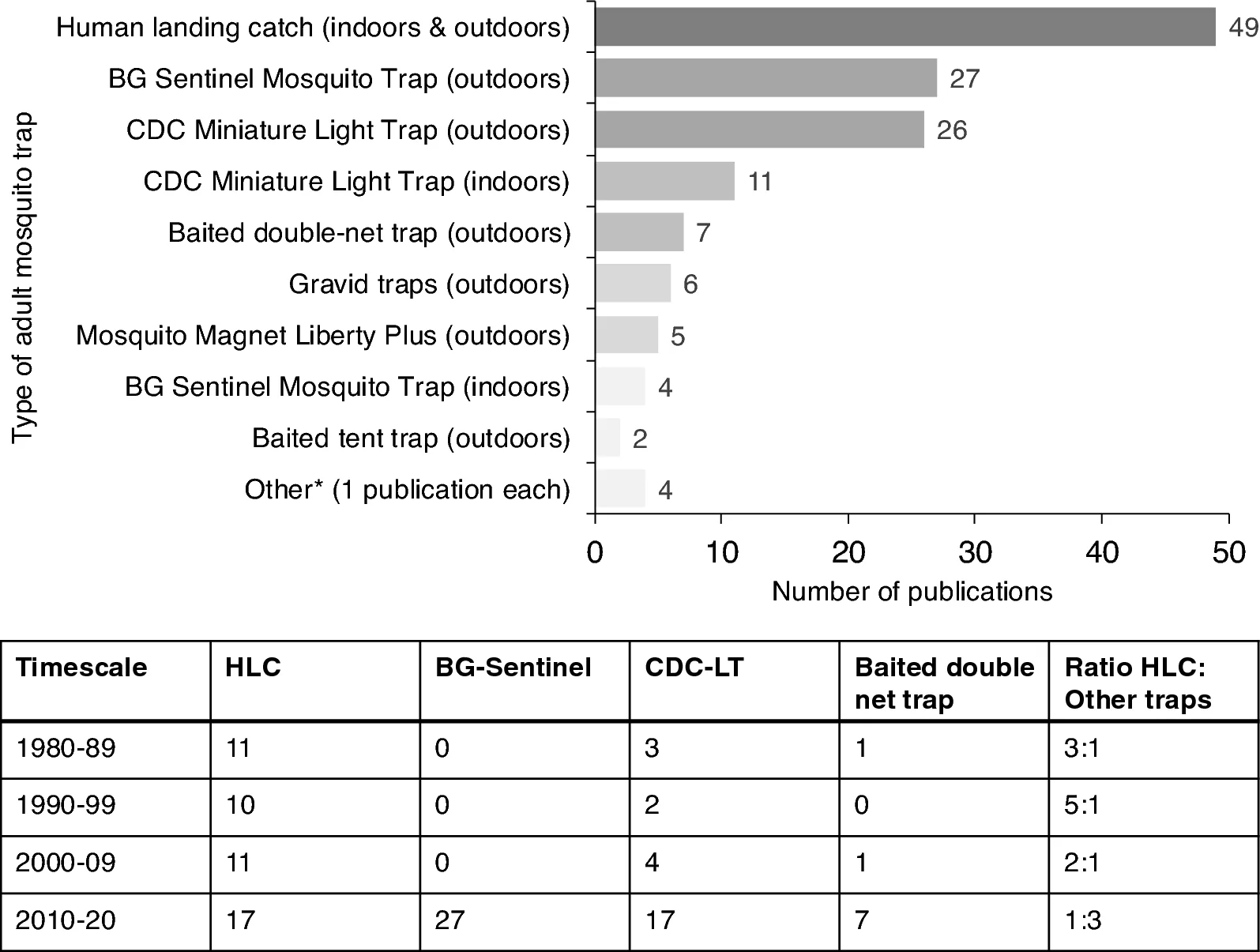
Number of publications featuring adult mosquito traps used to collect host-seeking adult *Aedes* mosquitoes and change in trapping methods used over ten year time intervals from 1980–2020

110 publications featured collection of adult host-seeking *Aedes*, with human-landing catch (HLC), BG Sentinel Traps (Biogents AG, Regensburg, Germany) and CDC Miniature Light Traps (US Centers for Disease Control and Prevention) the three most common trapping methods (Fig. 7). While HLC was usually conducted as paired indoor and outdoor collections, all other adult traps were predominantly used outdoors. There was a shift over time in trap choice with HLC the most common method during 1980–2009 (74% of studies), but BG-Sentinel, CDC-LT or baited double net traps were preferred during 2010–2020 over HLC (Fig. 7). The bait used to attract host-seeking mosquitoes to the traps varied between studies. Carbon dioxide was used in 29 publications, paired with CDC-LT (16 studies), BG Sentinel Traps (11), and Mosquito Magnet Liberty Plus (5) (American Biophysics Corporation, Rhode Island, USA) (Table S2.2). The source of CO_2_ was dry ice in 15 studies (from Kenya, Uganda and South Africa), sugar/yeast in three (Ghana, Cameroon, Kenya), propane tanks for the Mosquito Magnet Liberty Plus (Tanzania) and CO_2_-filled bottles in Mauritania. Other attractants used to increase trap efficiency were the standard BG-lure[115], varying numbers of live mice contained inside BG-Sentinel traps [116, 117], light traps baited with sheep skin volatiles [118], live sheep, goats, chickens or pigeons [119] or setting traps near animal enclosures [115].

In addition to collections of host-seeking adult mosquitoes, resting collections were also conducted in 48 studies. Methods included indoor pyrethrum spray catch (*n = *21 publications), mechanical aspiration (*n = *17 outdoors, *n = *15 indoors), mouth aspiration (*n = *12), sweep nets (*n = *6) and resting boxes (*n = *4) (Table S2.3).

#### Adult *Aedes* bionomics; sources of blood meals and biting times

There were 16 publications that reported *Aedes* blood–meal sources, with the number of samples generally low for arbovirus vector species of interest. Four publications determined that humans were the primary blood–meal source for *Ae. aegypti* and *Ae. aegypti formosus*, while two studies from Kenya showed *Ae. aegypti* plasticity with a high proportion having fed on domesticated and sylvatic animals (Table 7). Sylvatic species *Ae. taylori* and *Ae. vittatus* fed on non-human primates, which are potential arbovirus reservoirs, in a forested area of Senegal [120].

**Table 7:** Publications which determined blood–meal hosts of *Aedes* (minimum sample size > 20)

Mosquito species	Year of study	Country and setting (reference)	Trap type	Location (in/out)	% human-fed	Primary blood-meal host
*Ae. aegypti*	2014–18	Kenya; urban sites, Kisumu, Mombasa, Nairobi [48]	BG-Sentinel with CO^2^, mechanical aspirator	BG outdoors, aspirator indoors	24% (24/102)	Several animal species (cow, goat, dog, cat)
*Ae. aegypti*	2015–16	Kenya; settlements near caves, West Pokot [121]	BG-Sentinel with CO^2^	Outdoors, peridomestic	4% (3/68)	Rock hyrax 79% (54/68)
*Ae. aegypti*	2016–17	Burkina Faso; urban, peri urban, rural sites near Ouagadougou [17]	Mechanical aspirator	Indoors and outdoors	88% (158/180)	Human
*Ae. aegypti*	2017–18	Ghana; urban, peri urban, rural sites in Sahel savannah [122]	BG-Sentinel, mechanical aspirator	Indoors and outdoors	90% (18/20)	Human
*Ae. aegypti*	2019	Senegal; four sites, Aaa from Dakar, Barkedji, Louga; Aaf were from Kedougou (forested) [115]	BG-Sentinel, mechanical aspirator	Indoors and outdoors	92.3% (1,056/1,138)	Human
*Ae. aegypti formosus*					86.2% (319/370)	
*Ae. aegypti*	2019	Senegal; 5 locations across Senegal [123]	BG-Sentinel, mechanical aspirator	Indoors and outdoors	78.6% (964/1,226)	Human
*Ae. taylori*, *Ae. vittatus*	2010–11	Senegal; natural forest sites, Kedougou [120]	Mechanical aspirator	Indoors and outdoors	0% (0/14) 0% (0/16)	Non-human primates and avian hosts

Biting times of *Aedes* species were reported in 13 publications, with *Ae. aegypti* the main species studied (*n = *11) (Table 8). The late afternoon period was the primary biting peak for *Ae. aegypti*, with an early morning peak either absent or at a far lower rate (Table 8). However, several studies showed *Ae. aegypti* biting at night, with the major indoor-biting peak between 9 pm-midnight in rural Côte d’Ivoire [124] and extended biting peaks reported after sunset in rural and urban Kenya [125], Niger [126], Nigeria [127], Mali [128] and Senegal [116]. Biting times were substantially different in open versus shaded areas outdoors in Mali, with biting earlier in the morning and later in the afternoon in open, lighter locations [128]. In Senegal biting was later in the morning and earlier in the afternoon during the cool, dry season as compared to the hot, rainy seasons [115]. In La Réunion *Ae. albopictus* seasonal differences were reported based on the time of sunset, with the afternoon outdoor biting peak occurring consistently 2 h before sunset regardless of season [129].

**Table 8:** Publications which monitored arbovirus vector species host-seeking periodicity

Mosquito species	Year of study	Country (reference)	Trapping method	Trapping period	Primary peak	Secondary peak
*Ae. aegypti* *Ae. vittatus*	1988–89	Nigeria [130]	Emergent trap (septic tanks)^a^	24 h	4–6 pm 4–6 pm	4–6 am 4–6 am
*Ae. aegypti*	1996–97	Côte d’Ivoire [124]	HLC (out/in)	12 h: 6 pm–6 am	9 pm-midnight	NA
*Ae. aegypti*	2002–17	Niger [126]	HLC (out/in)	12 h: 7 pm–7 am	7 pm-1am	NA
*Ae. aegypti* *Ae. luteocephalus*	2014–15	Nigeria [127]	HLC (out/in)	24 h	5–8 pm 7–9 pm	NA
*Ae. aegypti*	2014–16	Kenya [131]	HLC (out)	10 h: 5:30–11:30 am 3:30–7:30 pm	3:30–6:30 pm	5:30–7:30am
*Ae. aegypti*	2015–16	Ghana [132]	HLC (out/in)	12 h: 6 am–6 pm	3–4 pm	6-8am
*Ae. aegypti*	2016	Mali [128]	HLC (out)	18.5 h: 5 am–11:30 pm	8–9 pm^b^ 6–8:30pm^c^	7–8:30 am^b^ 9–10:30 am^c^
*Ae. aegypti*	2017–18	Nigeria [133]	HLC (out)	12 h: 6 pm–6am	7–9 pm	NA
*Ae. aegypti*	2017–18	Kenya [125]	HLC (out/in)	17 h: 4:30 am–9:30 pm	Diurnal, not clear	Some nocturnal
*Ae. aegypti*	2018	Benin [134]	HLC (out/in)	11 h: 6 am–midday, 3–8 pm	5–6 pm	8–9am
*Ae. aegypti*	2019	Senegal [116]	BG-Sentinel (out)	14 h: 6 am–8 pm	4–6 pm^d^ 6–8 pm^e^	8–10 am^d^ 6–8 am^e^
*Ae. albopictus*	Nd	La Réunion [129]	Human-baited double net trap (out/in)	24 h	^f^4 pm/5:30 pm	8:30 am
*Ae. albopictus*	2009	Cameroon [135]	Human-baited double net trap (out)	24 h	4–6 pm	NA

*out* outdoors, *in *indoors (trap location)

^a^Emergent traps monitor activity rather than host-seeking per se

^b^Open areas

^c^Shady areas

^d^Cool, dry season

^e^Hot dry and rainy seasons

^f^Peak at 4 pm in winter, 5:30 pm in summer; always 2 h before sunset outdoors

## Discussion

Mosquito identification keys remain essential for accurate species identification, which can be supported by molecular techniques. Unlike for the study of *Anopheles* in Africa, there is no single comprehensive key for all African *Aedes* species, which may partly explain the wide variety of references cited. However, Huang, 2001 [136] can be used to identify samples down to subgenus level, and the *Stegomyia* keys of Huang 2004 [35] and Rueda 2004 [34] include many of the most important African arbovirus vector species including *Ae. aegypti*, *Ae. albopictus*, *Ae. africanus*, *Ae. luteocephalus*, *Ae. opok*, *Ae. neoafricanus*, *Ae. metallicus*, *Ae. simpsoni* group, and *Ae. unilineatus*. When studying sylvatic or bridge vectors additional keys for other subgenera may be required such as *Diceromyia* (e.g. *Ae. furcifer*, *Ae. taylori*) (Huang and Rueda, 2016 [137]) and *Ochlerotatus* (e.g. *Ae. caspius*) (Huang and Rueda, 2014 [138]. There is also an excellent regional illustrated West Africa key covering many of the most important *Aedes* arbovirus species by Robert et al., 2022 [139].

Vector competence studies are crucial to explain and assess potential arbovirus epidemic risk so that preventative measures such as vector control can be established [140]. Studies of CHIKV, DENV and ZIKV demonstrated how vector competence is highly dependent on the specific virus strain and mosquito population, highlighting the ongoing and often rapid coevolution of mosquito and virus [141]. A similar observation to the finding that African *Ae. albopictus* populations have a strong midgut barrier to Asian ZIKV genotypes has also been reported for *Ae. albopictus* in France, which were able to transmit the African genotype of ZIKV better than the Asian genotype, stressing the importance of each pairing of mosquito population and virus strain [71, 142]. Vector competence studies were restricted to a handful of countries and ecological settings, highlighting knowledge gaps regarding the competence of different vector species to major arboviruses across urban, peri-urban and sylvatic settings. This gap likely reflects constraints in infrastructure and specialized laboratory capacity. Given the ongoing spread of *Ae. albopictus* through Africa and its established role as a primary vector of CHIKV, there is a need for further vector competence studies from a wider geographical area, using relevant virus strains that are in circulation. In addition to the primary urban and peri-urban species *Ae. aegypti* and *Ae. albopictus*, there were 16 secondary *Aedes* species identified that either demonstrated vector competence in laboratory studies or from which wild populations have tested positive for DENV, CHIKV, YFV, or ZIKV, although further work is required to determine their epidemiological importance in transmission cycles. Virus screening of potential vector species provides useful evidence towards implication of vectors involved in ongoing transmission, but several zero-detection studies highlight the importance of timing during active virus circulation and the need for large sample sizes. Arbovirus detection in *Aedes* vectors is generally more challenging and costly than detection of malaria parasites, due to typically low infection rates, requiring the processing of large sample sizes (several thousand mosquitoes) to reliably detect viral circulation and the need for a reliable cold chain to prevent degradation of RNA viruses [143–145]. Arbovirus surveillance in vectors needs improving as outbreaks become more widespread, affecting large populations [146]. A WHO-led capacity assessment identified gaps reported by most countries included a lack of training and retraining for staff involved in surveillance and control of arboviral diseases in addition to a lack of financial and technical support and community awareness [2].

Monitoring of immature and adult arbovirus vector species remains of critical importance to guide appropriate vector control measures for the local context. The traditional container and house/premises immature-stage indices developed in the 1920s, followed by the Breteau index in the 1950s, are included in several WHO arbovirus surveillance guidance documents [114]. All three indices were commonly reported together; however, there is limited evidence of association of these traditional indices with transmission risk and outbreak prediction [147, 148]. The pupal index was developed more recently in the 1990s to determine the productivity of container types and guide targeted vector control and is described in several WHO guideline documents [110, 149–151]. Amongst the articles reviewed, the pupal index was much less frequently reported than the three traditional indices but has been more widely used in recent years. Pupal counts are a better proxy for the number of adult mosquitoes being produced by a breeding site, so may be more relevant to transmission risk and can be used to identify key breeding containers for targeted control. The number of positive containers for larvae can be misleading as a large water storage container can be many times more productive than numerous positive small containers, such as coconut husks. Tires, other discarded items and water storage containers were the most frequently positive larval sites. Nonetheless, there was geographical variation depending on the location characteristics (presumably based on ecology and sociocultural/economic factors) highlighting the value of local data collection before the design and implementation of control campaigns. Elsewhere, the SUM method was developed in Asia, where the shape, use and material of containers are described to identify key containers that disproportionately produce the majority of *Ae. aegypti* [152]. Variations of this approach have been used globally in many studies to identify key containers to target for control, with each ecological setting having its own set of key *Aedes* containers.

Human landing catch, whilst being the gold standard for estimating human-vector contact and thus transmission risk, has come under scrutiny due to ethical concerns around exposing collectors to potential arbovirus infection, given the lack of effective vaccines or prophylaxis for most arboviruses, except for yellow fever [153, 154]. Alternative trapping methods such as BG-Sentinel and CDC-LT have been adopted, but it is important to conduct regional trapping comparisons where data are absent to ensure high trap efficiency, which can be improved with various attractants such as BG-lures, mice [117] or animal volatiles [118]. Where use of dry ice is not possible or prohibitively expensive, yeast and sugar as a source of CO_2_ may be a suitable alternative [122]. *Aedes aegypti* is regarded as a highly anthropophilic, day-biting species that is generally known for exhibiting bimodal biting in the early morning and late afternoon [155, 156], and indeed crepuscular biting was the pattern typically observed in most studies in this review. The atypical finding in Katiola, Côte d’Ivoire of nocturnal *Ae. aegypti* biting was attributed to the presence of an unusual number of jars and earthenware pots used for traditional medicines [124]. However, increased use of artificial lights has been demonstrated to extend *Ae. aegypti* biting [157]; therefore, periodic monitoring of biting times covering a 24 h period is recommended, across a range of settings to take account of change in human lifestyles. Similarly, findings from parts of Kenya showing substantial animal feeding by *Ae. aegypti* highlight the dynamic context-specific nature of host preference and the need for regional monitoring to assess relative host importance. It is well known that host preference is not static but is characterized by behavioral plasticity that allows mosquitoes to switch hosts based on availability [158, 159].

Despite availability of a yellow fever vaccine, there continue to be epidemics in Africa due to occasional urban spillover from sylvatic reservoirs, maintained by non-human primates and sylvatic *Aedes* such as *Ae. furcifer, Ae. taylori*, *Ae. luteocephalus*, and *Ae. africanus* [160]. However, study of sylvatic arbovirus vectors was generally neglected, with Senegal, Kenya, Côte d’Ivoire, Cameroon and South Africa being exceptions. With increasing deforestation, human encroachment and land use changes, sylvatic vector species composition and behavior is changing and should be monitored in more countries to better predict and understand outbreak risk [161].

## Conclusions

This review summarizes the current state of knowledge on arbovirus vector competence, bionomics and distribution of *Aedes* mosquitoes in Africa. It serves as a foundation for engaging stakeholders and advancing the research agenda toward strengthening evidence-based practices and standards for detecting and controlling arbovirus epidemics.

**Table:** 

Topic	Key recommendations/priorities for future research
Vector Competence	Studies needed across a wider geographical area, with priority given to using virus strains from recent epidemics and local mosquito populations, including sylvatic and bridge species. To achieve this greater investment is needed to strengthen local research infrastructure and capacity
Vector Bionomics	Pupal index data should be gathered and reported more routinely to quantify the most locally important *Aedes* breeding sites to target for control Alternatives to HLC such as the double-net trap, CDC-LT, or BG-Sentinel with human, animal, synthetic or CO_2_ attractants should be considered for trapping host-seeking *Aedes* More bionomic research is needed into blood–meal hosts and biting times, particularly with rapid urbanization and changing environments in Africa
Vector Distribution	Study of sylvatic vector species was limited to a handful of countries. A much greater understanding is needed to predict potential sources of future outbreaks, particularly in areas of yellow fever risk. Identification and monitoring of potential bridge vectors may also be useful to anticipate spillover of zoonotic arboviruses

## Supplementary Information

Additional file 1: Supplementary Tables S1.1–S2.7 list the publications included in this scoping review.

Additional file 2.

Additional file 3.

Additional file 4.

Additional file 5.

Additional file 6.

Additional file 7.

Additional file 8.

Additional file 9.

## Data Availability

All data sets are included in this published article and its supplementary information files.

## Abbreviations

Aaf: *Aedes aegypti formosus*
CDC-LT: Centers for Disease Control and Prevention-Light Trap
CHIKV: Chikungunya virus
DENV: Dengue virus
HLC: Human landing catch
LSHTM: London School of Hygiene and Tropical Medicine
PRISMA-ScR: Preferred Reporting Items for Systematic Reviews and Meta-Analyses Extension for Scoping Reviews
YFV: Yellow fever virus
ZIKV: Zika virus
