# Supplementary material for: Building resilience against the growing threat of arboviruses II: a scoping review of Aedes vector competence, larval and adult bionomics and species distribution in Africa
Source: Parasit Vectors. 2026 Jul 28;19:460. doi: 10.1186/s13071-026-07552-5 (PMC13693134; doi:10.1186/s13071-026-07552-5)
Supplement: Supplementary file 1 [file 13071_2026_7552_MOESM1_ESM.docx]

| **Supplementary Table S1.1 Vector-pathogen interactions; vector competence** | | | | | | |
| --- | --- | --- | --- | --- | --- | --- |
| **No.** | **Study** | **Publication Year** | **Country** | **Mosquito Species** | **Virus strains** | **Ref** |
| 1 | Jupp et al. | 1981 | South Africa | *Ae. furcifer* | ChikV | [1] |
| 2 | Powell | 1985 | Kenya, Senegal, The Gambia, Uganda | *Ae. aegypti*  *Ae. albopictus* | YFV (strain not described) | [13] |
| 3 | Miller et al. | 1989 | Nigeria | *Ae. aegypti*  *Ae. aegypti formosus* | YFV (isolated from Ogbomosho outbreak) | [29] |
| 4 | Vazeille-Falcoz et al. | 1999 | Gabon | *Ae. aegypti formosus* | DENV-2 (Rosen-Bangkok, 1974) | [11] |
| 5 | Vazeille et al. | 2001 | Madagascar | *Ae. aegypti*  *Ae. albopictus* | DENV-2 (Rosen-Bangkok, 1974) | [14] |
| 6 | Paupy et al. | 2001 | La Réunion | *Ae. albopictus* | DENV-2 (Rosen-Bangkok, 1974) | [5] |
| 7 | Jupp et al. | 2002 | South Africa | Several | YFV (BA55, TVPl617 Nigeria), (BC7914, Kenya) | [23] |
| 8 | Diallo et al. | 2005 | Senegal | *Ae. furcifer, Ae. luteocephalus, Ae. vitattus, Ae. aegypti* | DENV-2 (New Guinea, NGC, 1944), (1349, Burkina Faso, 1982), (PM33974, Guinea, 1981), (DakAr2022, Burkina Faso, 1980) | [37] |
| 9 | Vazeille et al. | 2007 | La Réunion  Mayotte | *Ae. albopictus* | CHIKV (05.115, 2005, Reunion), (06.21, 2005, Reunion), (06.111, 2006, Mayotte), (06.117, DR Congo, 1999-2000) | [15] |
| 10 | Diallo et al. | 2008 | Senegal | *Ae. aegypti* | DENV-2 (ArD 140875 (1999, Kedougou), ArA 6894 (1986, Bobo-Dioulasso) | [36] |
| 11 | Vazeille et al. | 2008 | Gabon | *Ae. albopictus* | DENV-2 (Rosen-Bangkok, 1974), CHIKV (06.21) | [2] |
| 12 | Sylla et al. | 2009 | Senegal | *Ae. aegypti*  *Ae. aegypti formosus* | DENV-2 (JAM1409, 1983, Jamaica, | [16] |
| 13 | Martin et al. | 2010 | La Réunion  Mayotte | *Ae. aegypti*  *Ae. albopictus* | CHIKV (E1-226A & E1-226V) | [27] |
| 14 | Paupy et al. | 2010 | Cameroon, Gabon | *Ae. aegypti*  *Ae. albopictus* | DENV-2 (D2S32), CHIKV (06.21, Reunion) | [17] |
| 15 | Vazeille et al. | 2010 | La Réunion | *Ae. albopictus* | CHIKV (06.21, 2005, Reunion), DENV-1 (185/04 , 2004, Reunion) | [18] |
| 16 | Moutailler et al. | 2010 | Madagascar | *Ae. aegypti*  *Ae. aegypti formosus* | DENV-2 (Rosen-Bangkok, 1974) | [30] |
| 17 | Mousson et al. | 2012 | La Réunion | *Ae. albopictus* | DENV-2 (Rosen-Bangkok, 1974) | [3] |
| 18 | Ellis et al. | 2012 | Kenya | *Ae. aegypti*  *Ae. simpsoni* | YFV (Sudan, 2003) | [39] |
| 19 | Vazeille et al. | 2013 | Cabo Verde | *Ae. aegypti formosus* | DENV-3 (2009, Cape Verde), DENV-2 (1974, Thailand), CHIKV (A226V, Reunion), YFV (1979, Senegal) | [19] |
| 20 | Gaye et al. | 2014 | Senegal | *Ae. aegypti*  *Ae. aegypti formosus* | DENV-1 (IbH28328, Ibadan, Nigeria 1964), DENV-3 (H87, 1957, Hawaii) | [8] |
| 21 | Chepkorir et al. | 2014 | Kenya | *Ae. aegypti* | DENV-2 (008/01/2012, Mandera, Kenya, 2012) | [33] |
| 22 | Diagne et al. | 2014 | Senegal, Cabo Verde | *Ae. aegypti*  *Ae. vittatus* | CHIKV (ArD30237, Kedougou, 1979), (CS13-288, Gagnik, 1962), (HD180738, Sine Saloum, 2005) | [35] |
| 23 | Dickson et al. | 2014 | Senegal | *Ae. aegypti* | DENV-2 (75505, Kedougou, Senegal, 1990), DENV-2 (JAM1409, Jamaica 1983), YFV (BA55, Nigeria, 1986), YFV (Dak1279, Diourbel, 1965) | [38] |
| 24 | Da Moura et al. | 2015 | Cabo Verde | *Ae. aegypti* | DENV-1 (42735/BR PE (GenBank Accession N° EU259529), DENV-2 (3808/BR-PE (EU259569), DENV-3 (85469/BR-PE (EU259607), DENV-4 (1385 (U1842). | [34] |
| 25 | Vazeille et al. | 2016 | Rep of Congo, La Réunion | *Ae. aegypti*  *Ae. albopictus* | CHIKV (DRC_2000 and Congo_2011 strains, DR Congo, 2000 & 2011), CHIKV (06.21, Reunion), CHIKV (05.115, Reunion) | [20] |
| 26 | Mbaika et al. | 2016 | Kenya | *Ae. aegypti* | CHIKV (Lamu, 2004-05, Kenya) | [28] |
| 27 | Agha et al. | 2017 | Kenya | *Ae. aegypti* | CHIKV (Lamu001 strain 2004-2005) | [40] |
| 28 | Vazeille et al. | 2018 | La Réunion | *Ae. albopictus* | ZIKV (2014, New Caledonia) | [21] |
| 29 | Mulwa et al. | 2018 | Kenya | *Ae. bromeliae Ae. vittatus* | CHIKV (Lamu, 001, 2004-05, Kenya) | [31] |
| 30 | Ben Ayed et al. | 2019 | Tunisia | *Ae. detritus Ae. caspius* | ZIKV Asian strain (NC-2014-5132) | [4] |
| 31 | Amraoui et al. | 2019 | Morocco | *Ae. albopictus* | CHIKV (06.21, Reunion), DENV-2 (Rosen-Bangkok, 1974), ZIKV (NC-2014-5132, 2014 New Caledonia), YFV (S79, 1979 Senegal) | [7] |
| 32 | Gaye et al. | 2019 | Senegal | *Ae. aegypti*  *Ae. aegypti formosus*  *Ae. furcifer* | DENV-1 (SH 29177, 1979, Senegal), DENV-3 (Carec 01–11828, 2001, Barbados), DENV-3 (S-162 TvP-3622, 1993, Somalia), DENV-4 (SH 38549, 1983, Dakar, Senegal), DENV-4 (Haiti 73, 1994, Haiti) | [9] |
| 33 | Kamgang et al. | 2019 | Rep of Congo, Cameroon | *Ae. aegypti*  *Ae. albopictus* | DENV-2 (Rosen-Bangkok, 1974) | [25] |
| 34 | Kamgang et al. | 2019 | Rep of Congo, Cameroon | *Ae. aegypti*  *Ae. albopictus* | YFV (S79, 1979, Senegal) | [26] |
| 35 | Agha et al. | 2019 | Kenya | *Ae. aegypti* | DENV-2 (Sample number:008/01/2012, Mandera, Kenya, 2012) | [41] |
| 36 | Mutebi et al. | 2020 | Uganda | *Ae. aegypti formosus* | ZIKV (MR766, Uganda, 1947) | [10] |
| 37 | Kamgang et al. | 2020 | Rep of Congo, Cameroon | *Ae. aegypti*  *Ae. albopictus* | ZIKV (Ae. taylori-tc/SEN/1984/41662-DAK, 1984, Senegal) | [24] |
| 38 | Bohers et al. | 2020 | Tunisia | *Ae. albopictus* | CHIKV (06.21, Reunion), DENV-2 (Rosen, Bangkok, 1974) and ZIKV (NC-2014-5132, New Caledonia, 2014) | [32] |
| 39 | Amoa-Bosompem et al. | 2021 | Ghana | *Ae. aegypti* | DENV-1 (strain D1/Hu/Saitama/NIID100/2014), DENV-2 (strain DENV-2/GH/NMIMR-BC-UG-F299/2017) | [6] |
| 40 | Obadia et al. | 2022 | Cameroon, Rep of Congo, Gabon, La Réunin | *Ae. aegypti*  *Ae. albopictus* | ZIKV (Dakar; KU955592, Senegal, 1984) ZIKV (Cambodia, KU955593, 2010), ZIKV (Malaysia, KX694533, 1966), ZIKV (LR792671.1, 2016) | [12] |
| 41 | Rose et al. | 2022 | Cabo Verde | *Ae. aegypti*  *Ae. aegypti formosus* | ZIKV (2010, Cambodia & 2011, Senegal strains) | [22] |

**References**

1.	Jupp PG, McIntosh BM, Dos Santos I, DeMoor P: **Laboratory vector studies on six mosquito and one tick species with chikungunya virus.** *Trans R Soc Trop Med Hyg* 1981, **75:**15-19.

2.	Vazeille M, Moutailler S, Pages F, Jarjaval F, Failloux AB: **Introduction of Aedes albopictus in Gabon: what consequences for dengue and chikungunya transmission?** *Trop Med Int Health* 2008, **13:**1176-1179.

3.	Mousson L, Zouache K, Arias-Goeta C, Raquin V, Mavingui P, Failloux AB: **The native Wolbachia symbionts limit transmission of dengue virus in Aedes albopictus.** *PLoS Negl Trop Dis* 2012, **6:**e1989.

4.	Ben Ayed W, Amraoui F, M'ghirbi Y, Schaffner F, Rhaim A, Failloux AB, Bouattour A: **A Survey of Aedes (Diptera: Culicidae) Mosquitoes in Tunisia and the Potential Role of Aedes detritus and Aedes caspius in the Transmission of Zika Virus.** *J Med Entomol* 2019, **56:**1377-1383.

5.	Paupy C, Girod R, Salvan M, Rodhain F, Failloux AB: **Population structure of Aedes albopictus from La Réunion Island (Indian Ocean) with respect to susceptibility to a dengue virus.** *Heredity (Edinb)* 2001, **87:**273-283.

6.	Amoa-Bosompem M, Kobayashi D, Itokawa K, Murota K, Faizah AN, Azerigyik FA, Hayashi T, Ohashi M, Bonney JHK, Dadzie S, et al: **Determining vector competence of Aedes aegypti from Ghana in transmitting dengue virus serotypes 1 and 2.** *Parasit Vectors* 2021, **14:**228.

7.	Amraoui F, Ben Ayed W, Madec Y, Faraj C, Himmi O, Btissam A, Sarih M, Failloux AB: **Potential of Aedes albopictus to cause the emergence of arboviruses in Morocco.** *PLoS Negl Trop Dis* 2019, **13:**e0006997.

8.	Gaye A, Faye O, Diagne CT, Diallo D, Weaver SC, Sall AA, Diallo M: **Oral susceptibility of Aedes aegypti (Diptera: Culicidae) from Senegal for dengue serotypes 1 and 3 viruses.** *Trop Med Int Health* 2014, **19:**1355-1359.

9.	Gaye A, Wang E, Vasilakis N, Guzman H, Diallo D, Talla C, Ba Y, Dia I, Weaver SC, Diallo M: **Potential for sylvatic and urban Aedes mosquitoes from Senegal to transmit the new emerging dengue serotypes 1, 3 and 4 in West Africa.** *PLoS Negl Trop Dis* 2019, **13:**e0007043.

10.	Mutebi JP, Lutwama JJ: **Vector Competence of Aedes aegypti Populations from Entebbe, Uganda, for Zika Virus.** *Am J Trop Med Hyg* 2020, **104:**564-566.

11.	Vazeille-Falcoz M, Failloux AB, Mousson L, Elissa N, Rodhain F: **[Oral receptivity of Aedes aegypti formosus from Franceville (Gabon, central Africa) for type 2 dengue virus].** *Bull Soc Pathol Exot* 1999, **92:**341-342.

12.	Obadia T, Gutierrez-Bugallo G, Duong V, Nuñez AI, Fernandes RS, Kamgang B, Hery L, Gomard Y, Abbo SR, Jiolle D, et al: **Zika vector competence data reveals risks of outbreaks: the contribution of the European ZIKAlliance project.** *Nat Commun* 2022, **13:**4490.

13.	Powell JR: **Geographic genetic differentiation and arbovirus competency: Aedes aegypti and yellow fever.** *Parassitologia* 1985, **27:**13-20.

14.	Vazeille M, Mousson L, Rakatoarivony I, Villeret R, Rodhain F, Duchemin JB, Failloux AB: **Population genetic structure and competence as a vector for dengue type 2 virus of Aedes aegypti and Aedes albopictus from Madagascar.** *Am J Trop Med Hyg* 2001, **65:**491-497.

15.	Vazeille M, Moutailler S, Coudrier D, Rousseaux C, Khun H, Huerre M, Thiria J, Dehecq JS, Fontenille D, Schuffenecker I, et al: **Two Chikungunya isolates from the outbreak of La Reunion (Indian Ocean) exhibit different patterns of infection in the mosquito, Aedes albopictus.** *PLoS One* 2007, **2:**e1168.

16.	Sylla M, Bosio C, Urdaneta-Marquez L, Ndiaye M, Black WC: **Gene flow, subspecies composition, and dengue virus-2 susceptibility among Aedes aegypti collections in Senegal.** *PLoS Negl Trop Dis* 2009, **3:**e408.

17.	Paupy C, Ollomo B, Kamgang B, Moutailler S, Rousset D, Demanou M, Hervé JP, Leroy E, Simard F: **Comparative role of Aedes albopictus and Aedes aegypti in the emergence of Dengue and Chikungunya in central Africa.** *Vector Borne Zoonotic Dis* 2010, **10:**259-266.

18.	Vazeille M, Mousson L, Martin E, Failloux AB: **Orally co-Infected Aedes albopictus from La Reunion Island, Indian Ocean, can deliver both dengue and chikungunya infectious viral particles in their saliva.** *PLoS Negl Trop Dis* 2010, **4:**e706.

19.	Vazeille M, Yébakima A, Lourenço-de-Oliveira R, Andriamahefazafy B, Correira A, Rodrigues JM, Veiga A, Moreira A, Leparc-Goffart I, Grandadam M, Failloux AB: **Oral receptivity of Aedes aegypti from Cape Verde for yellow fever, dengue, and chikungunya viruses.** *Vector Borne Zoonotic Dis* 2013, **13:**37-40.

20.	Vazeille M, Zouache K, Vega-Rúa A, Thiberge JM, Caro V, Yébakima A, Mousson L, Piorkowski G, Dauga C, Vaney MC, et al: **Importance of mosquito "quasispecies" in selecting an epidemic arthropod-borne virus.** *Sci Rep* 2016, **6:**29564.

21.	Vazeille M, Dehecq JS, Failloux AB: **Vectorial status of the Asian tiger mosquito Aedes albopictus of La Réunion Island for Zika virus.** *Med Vet Entomol* 2018, **32:**251-254.

22.	Rose NH, Dabo S, da Veiga Leal S, Sylla M, Diagne CT, Faye O, Sall AA, McBride CS, Lambrechts L: **Enhanced mosquito vectorial capacity underlies the Cape Verde Zika epidemic.** *PLoS Biol* 2022, **20:**e3001864.

23.	Jupp PG, Kemp A: **Laboratory vector competence experiments with yellow fever virus and five South African mosquito species including Aedes aegypti.** *Trans R Soc Trop Med Hyg* 2002, **96:**493-498.

24.	Kamgang B, Vazeille M, Tedjou A, Yougang AP, Wilson-Bahun TA, Mousson L, Wondji CS, Failloux AB: **Different populations of Aedes aegypti and Aedes albopictus (Diptera: Culicidae) from Central Africa are susceptible to Zika virus infection.** *PLoS Negl Trop Dis* 2020, **14:**e0008163.

25.	Kamgang B, Vazeille M, Tedjou AN, Wilson-Bahun TA, Yougang AP, Mousson L, Wondji CS, Failloux AB: **Risk of dengue in Central Africa: Vector competence studies with Aedes aegypti and Aedes albopictus (Diptera: Culicidae) populations and dengue 2 virus.** *PLoS Negl Trop Dis* 2019, **13:**e0007985.

26.	Kamgang B, Vazeille M, Yougang AP, Tedjou AN, Wilson-Bahun TA, Mousson L, Wondji CS, Failloux AB: **Potential of  *Aedes albopictus*   and   *Aedes aegypti* (Diptera: Culicidae) to transmit yellow fever virus in urban areas in Central Africa.** *Emerg Microbes Infect* 2019, **8:**1636-1641.

27.	Martin E, Moutailler S, Madec Y, Failloux AB: **Differential responses of the mosquito Aedes albopictus from the Indian Ocean region to two chikungunya isolates.** *BMC Ecol* 2010, **10:**8.

28.	Mbaika S, Lutomiah J, Chepkorir E, Mulwa F, Khayeka-Wandabwa C, Tigoi C, Oyoo-Okoth E, Mutisya J, Ng'ang'a Z, Sang R: **Vector competence of Aedes aegypti in transmitting Chikungunya virus: effects and implications of extrinsic incubation temperature on dissemination and infection rates.** *Virol J* 2016, **13:**114.

29.	Miller BR, Monath TP, Tabachnick WJ, Ezike VI: **Epidemic yellow fever caused by an incompetent mosquito vector.** *Trop Med Parasitol* 1989, **40:**396-399.

30.	Moutailler S, Guichoux E, Vaizelle M, Failloux A: **Differential mortalities of dengue-infected Aedes aegypti: preliminary results.** *Annales de la Societe Entomologique de France* 2010.

31.	Mulwa F, Lutomiah J, Chepkorir E, Okello S, Eyase F, Tigoi C, Kahato M, Sang R: **Vector competence of Aedes bromeliae and Aedes vitattus mosquito populations from Kenya for chikungunya virus.** *PLoS Negl Trop Dis* 2018, **12:**e0006746.

32.	Bohers C, Mousson L, Madec Y, Vazeille M, Rhim A, M'ghirbi Y, Bouattour A, Failloux AB: **The recently introduced Aedes albopictus in Tunisia has the potential to transmit chikungunya, dengue and Zika viruses.** *PLoS Negl Trop Dis* 2020, **14:**e0008475.

33.	Chepkorir E, Lutomiah J, Mutisya J, Mulwa F, Limbaso K, Orindi B, Ng'ang'a Z, Sang R: **Vector competence of Aedes aegypti populations from Kilifi and Nairobi for dengue 2 virus and the influence of temperature.** *Parasit Vectors* 2014, **7:**435.

34.	da Moura AJ, de Melo Santos MA, Oliveira CM, Guedes DR, de Carvalho-Leandro D, da Cruz Brito ML, Rocha HD, Gómez LF, Ayres CF: **Vector competence of the Aedes aegypti population from Santiago Island, Cape Verde, to different serotypes of dengue virus.** *Parasit Vectors* 2015, **8:**114.

35.	Diagne CT, Faye O, Guerbois M, Knight R, Diallo D, Ba Y, Dia I, Weaver SC, Sall AA, Diallo M: **Vector competence of Aedes aegypti and Aedes vittatus (Diptera: Culicidae) from Senegal and Cape Verde archipelago for West African lineages of chikungunya virus.** *Am J Trop Med Hyg* 2014, **91:**635-641.

36.	Diallo M, Ba Y, Faye O, Soumare ML, Dia I, Sall AA: **Vector competence of Aedes aegypti populations from Senegal for sylvatic and epidemic dengue 2 virus isolated in West Africa.** *Trans R Soc Trop Med Hyg* 2008, **102:**493-498.

37.	Diallo M, Sall AA, Moncayo AC, Ba Y, Fernandez Z, Ortiz D, Coffey LL, Mathiot C, Tesh RB, Weaver SC: **Potential role of sylvatic and domestic African mosquito species in dengue emergence.** *Am J Trop Med Hyg* 2005, **73:**445-449.

38.	Dickson LB, Sanchez-Vargas I, Sylla M, Fleming K, Black WC: **Vector competence in West African Aedes aegypti Is Flavivirus species and genotype dependent.** *PLoS Negl Trop Dis* 2014, **8:**e3153.

39.	Ellis BR, Sang RC, Horne KM, Higgs S, Wesson DM: **Yellow fever virus susceptibility of two mosquito vectors from Kenya, East Africa.** *Trans R Soc Trop Med Hyg* 2012, **106:**387-389.

40.	Agha SB, Chepkorir E, Mulwa F, Tigoi C, Arum S, Guarido MM, Ambala P, Chelangat B, Lutomiah J, Tchouassi DP, et al: **Vector competence of populations of Aedes aegypti from three distinct cities in Kenya for chikungunya virus.** *PLoS Negl Trop Dis* 2017, **11:**e0005860.

41.	Agha SB, Tchouassi DP, Turell MJ, Bastos ADS, Sang R: **Entomological assessment of dengue virus transmission risk in three urban areas of Kenya.** *PLoS Negl Trop Dis* 2019, **13:**e0007686.
