# Supplementary material for: Building resilience against the growing threat of arboviruses II: a scoping review of Aedes vector competence, larval and adult bionomics and species distribution in Africa
Source: Parasit Vectors. 2026 Jul 28;19:460. doi: 10.1186/s13071-026-07552-5 (PMC13693134; doi:10.1186/s13071-026-07552-5)
Supplement: Supplementary file 2 [file 13071_2026_7552_MOESM2_ESM.docx]

| **Supplementary Table S1.2 Vector competence; mosquito arbovirus prevalence** | | | | | | |
| --- | --- | --- | --- | --- | --- | --- |
| No. | Study | Publication Year | Country | Mosquito Species | Virus | Ref |
| 1 | Roche et al. | 1983 | Côte d’Ivoire Burkina Faso | *Ae. furcifer*  *Ae. luteocephalus* | DENV-2 | [1] |
| 2 | Cornet et al. | 1984 | Senegal | *Ae. furcifer*  *Ae. taylori*  *Ae. luteocephalus*  *Ae. aegypti + more* | DENV-2 | [2] |
| 3 | Monlun et al. | 1993 | Senegal | *Ae. furcifer*  *Ae. taylori*  *Ae. luteocephalus*  *Ae. dalzielii* | YFV | [3] |
| 4 | Robert et al. | 1993 | Burkina Faso | *Ae. aegypti*  *Ae. luteocephalus*  *Ae. opok*  *Ae. africanus*  *Ae. metallicus*  *Ae. unilieatus* | YFV | [4] |
| 5 | Traore-Lamizana et al. | 1996 | Senegal | *Ae. furcifer*  *Ae. taylori*  *Ae. luteocephalus* | YFV | [5] |
| 6 | Thonnon et al. | 1998 | Senegal | *Ae. aegypti*  *Ae. luteocephalus*  *Ae. metallicus* | YFV | [6] |
| 7 | Thonnon et al. | 1999 | Senegal | *Ae. aegypti* | CHIKV, YFV | [7] |
| 8 | Akoua-Koffi et al. | 2001 | Côte d’Ivoire | *Ae. aegypti* | YFV | [8] |
| 9 | Faye et al. | 2007 | Senegal | *Ae. aegypti*  *Ae. furcifer*  *Ae. luteocephalus*  *Ae. taylori*  *Ae. metallicus* | YFV | [9] |
| 10 | Sang et al. | 2008 | Comoros | *Ae. aegypti*  *Ae. simpsoni* | CHIKV | [10] |
| 11 | Crabtree et al. | 2009 | Kenya | *Ae. mcintoshi*  *Ae. ochraceus* | WNV, BUNV | [11] |
| 12 | Paupy et al. | 2010 | Cameroon | *Ae. aegypti*  *Ae. albopictus* | DENV-2, CHIKV | [12] |
| 13 | Diallo et al. | 2013 | Senegal | *Ae. aegypti*  *Ae. furcifer*  *Ae. vittatus* | YFV | [13] |
| 14 | Kone et al. | 2013 | Côte d’Ivoire | *Ae. aegypti*  *Ae. luteocephalus*  *Ae. opok* | YFV | [14] |
| 15 | Konan et al. | 2014 | Côte d’Ivoire | *Ae. aegypti*  *Ae. africanus*  *Ae. luteocephalus*  *Ae. vittatus*  *Ae. opok* + more | YFV | [15] |
| 16 | Masaninga et al. | 2014 | Zambia | *Ae. aegypti*  *Ae. minutis*  *Ae. mutilus* | YFV | [16] |
| 17 | Diallo et al. | 2014 | Senegal | *Ae. furcifer*  *Ae. dalzielii*  *Ae. africanus*  *Ae. taylori*  *Ae. metallicus*  *Ae. hirsutus*  *Ae. luteocephalus*  *Ae. aegypti* | ZIKV | [17] |
| 18 | Diallo et al. | 2014 | Senegal | *Ae. furcifer*  *Ae. dalzielii*  *Ae. africanus*  *Ae. taylori*  *Ae. luteocephalus*  *Ae. aegypti*  *Ae. vittatus*  *Ae. mcintoshi* | YFV | [18] |
| 19 | Lutomiah et al. | 2016 | Kenya | *Ae. aegypti*  *Ae. tricholabis* | DENV | [19] |
| 20 | Mboera et al. | 2016 | Tanzania | *Ae. aegypti* | DENV | [20] |
| 21 | Kajeguka et al. | 2017 | Tanzania | *Ae. aegypti* | DENV, CHIKV | [21] |
| 22 | Iwashita et al. | 2018 | Kenya | *Ae. aegypti* | DENV, YFV, WNV, ONYV, CHIKV, RVF | [22] |
| 23 | Kone et al. | 2018 | Côte d’Ivoire | *Ae. aegypti* | DENV-3 | [23] |
| 24 | Ndiaye et al. | 2018 | Senegal | *Ae. aegypti*  *Ae. furcifer*  *Ae. dalzielii*  *Ae. vexans*  *Ae. minutus*  *Ae. sudanensis* | WNV, Barkedji, Usutu, Sindbis etc. | [24] |
| 25 | Selhorst et al. | 2020 | DR Congo | *Ae. aegypti*  *Ae. albopictus* | CHIKV | [25] |
| 26 | Heath et al. | 2020 | Kenya | *Ae. aegypti* | ZIKV, CHIKV, DENV | [26] |
| 27 | Diallo et al. | 2020 | Senegal | *Ae. furcifer*  *Ae. dalzielii*  *Ae. luteocephalus*  *Ae. vittatus*  *Ae. aegypti formosus*  *Ae. minutus* | CHIKV, YFV, ZIKV | [27] |
| 28 | Diouf et al. | 2020 | Senegal | *Ae. aegypti*  *Ae. vittatus*  *Ae. taylori*  *Ae. luteocephalus*  *Ae. furcifer*  *Ae. dalzielii* + more | ZIKV | [28] |
| 29 | De Weggheleire et al. | 2021 | DR Congo | *Ae. aegypti* | CHIKV | [29] |
| 30 | Chiuya et al. | 2021 | Kenya | *Ae. aegypti*  *Ae. africanus*  *Ae. metallicus*  *Ae. tricholabis*  *Ae. mcintoshi*  *Ae. simpsoni* | DENV, CHIKV, YFV, WNV | [30] |
| 31 | Ngingo et al. | 2022 | Tanzania | *Ae. aegypti* | DENV | [31] |
| 32 | Tchibozo et al. | 2022 | Benin | *Ae. aegypti* | DENV-3 | [32] |
| 33 | El-Kady et al. | 2022 | Egypt | *Ae. aegypti* | DENV-2 | [33] |

**References**

1.	JC R, R C, JP H: **Ninety-six strains of dengue 2 virus isolated from mosquitoes collected in Ivory Coast and Upper Volta. [French].** *Annales de Virologie* 1983.

2.	Cornet M, Saluzzo J-F, Hervy J-P, Digoutte J-P, Germain M, Chauvancy M-F, Eyraud M, Ferrara L, Heme G, Legros F: **Dengue 2 au Sénégal oriental :  une poussée épizootique  en milieu selvatique ;  isolements du virus  à partir de moustiques  et d'un singe  et considérations épidémiologiques.** *Cah ORSTOM, sér Ent méd et Parasitol* 1984, **vol. XXII, no 4,:**313-323.

3.	Monlun E, Zeller H, Le Guenno B, Traoré-Lamizana M, Hervy JP, Adam F, Ferrara L, Fontenille D, Sylla R, Mondo M: **[Surveillance of the circulation of arbovirus of medical interest in the region of eastern Senegal].** *Bull Soc Pathol Exot* 1993, **86:**21-28.

4.	Robert V, Lhuillier M, Meunier D, Sarthou JL, Monteny N, Digoutte JP, Cornet M, Germain M, Cordellier R: **[Yellow fever virus, dengue 2 and other arboviruses isolated from mosquitos, in Burkina Faso, from 1983 to 1986. Entomological and epidemiological considerations].** *Bull Soc Pathol Exot* 1993, **86:**90-100.

5.	Traoré-Lamizana M, Fontenille D, Zeller HG, Mondo M, Diallo M, Adam F, Eyraud M, Maiga A, Digoutte JP: **Surveillance for yellow fever virus in eastern Senegal during 1993.** *J Med Entomol* 1996, **33:**760-765.

6.	Thonnon J, Spiegel A, Diallo M, Sylla R, Fall A, Mondo M, Fontenille D: **Yellow fever outbreak in Kaffrine, Senegal 1996: epidemiological and entomological findings.** *Trop Med Int Health* 1998, **3:**872-877.

7.	Thonnon J, Spiegel A, Diallo M, Diallo A, Fontenille D: **[Chikungunya virus outbreak in Senegal in 1996 and 1997].** *Bull Soc Pathol Exot* 1999, **92:**79-82.

8.	Akoua-Koffi C, Diarrassouba S, Bénié VB, Ngbichi JM, Bozoua T, Bosson A, Akran V, Carnevale P, Ehouman A: **[Investigation surrounding a fatal case of yellow fever in Côte d'Ivoire in 1999].** *Bull Soc Pathol Exot* 2001, **94:**227-230.

9.	Faye O, Diallo M, Dia I, Ba Y, Mondo M, Sylla R, Faye PC, Sall AA: **[Integrated approach to yellow fever surveillance: pilot study in Senegal in 2003-2004].** *Bull Soc Pathol Exot* 2007, **100:**187-192.

10.	Sang RC, Ahmed O, Faye O, Kelly CL, Yahaya AA, Mmadi I, Toilibou A, Sergon K, Brown J, Agata N, et al: **Entomologic investigations of a chikungunya virus epidemic in the Union of the Comoros, 2005.** *Am J Trop Med Hyg* 2008, **78:**77-82.

11.	Crabtree M, Sang R, Lutomiah J, Richardson J, Miller B: **Arbovirus surveillance of mosquitoes collected at sites of active Rift Valley fever virus transmission: Kenya, 2006-2007.** *J Med Entomol* 2009, **46:**961-964.

12.	Paupy C, Ollomo B, Kamgang B, Moutailler S, Rousset D, Demanou M, Hervé JP, Leroy E, Simard F: **Comparative role of Aedes albopictus and Aedes aegypti in the emergence of Dengue and Chikungunya in central Africa.** *Vector Borne Zoonotic Dis* 2010, **10:**259-266.

13.	Diallo M, Tall A, Dia I, Ba Y, Sarr FD, Ly A, Faye J, Badiane A, Diakhaby G, Faye P, et al: **Yellow fever outbreak in central part of Senegal 2002: epidemiological findings.** *Journal of Public Health and Epidemiology* 2013, **5-7:**291-296.

14.	Kone AB, Konan YL, Coulibaly ZI, Fofana D, Guindo-Coulibaly N, Diallo M, Doannio JM, Ekra KD, Odehouri-Koudou P: **[Entomological evaluation of the risk of urban outbreak of yellow fever in 2008 in Abidjan, Côte d'Ivoire].** *Med Sante Trop* 2013, **23:**66-71.

15.	Konan YL, Coulibaly ZI, Allali KB, Tétchi SM, Koné AB, Coulibaly D, Ekra KD, Doannio JM, Oudéhouri-Koudou P: **[Management of the yellow fever epidemic in 2010 in Séguéla (Côte d'Ivoire): value of multidisciplinary investigation].** *Sante Publique* 2014, **26:**859-867.

16.	Masaninga F, Muleba M, Masendu H, Songolo P, Mweene-Ndumba I, Mazaba-Liwewe ML, Kamuliwo M, Ameneshewa B, Siziya S, Babaniyi OA: **Distribution of yellow fever vectors in Northwestern and Western Provinces, Zambia.** *Asian Pac J Trop Med* 2014, **7S1:**S88-92.

17.	Diallo D, Sall AA, Diagne CT, Faye O, Ba Y, Hanley KA, Buenemann M, Weaver SC, Diallo M: **Zika virus emergence in mosquitoes in southeastern Senegal, 2011.** *PLoS One* 2014, **9:**e109442.

18.	Diallo D, Sall AA, Diagne CT, Faye O, Hanley KA, Buenemann M, Ba Y, Weaver SC, Diallo M: **Patterns of a sylvatic yellow fever virus amplification in southeastern Senegal, 2010.** *Am J Trop Med Hyg* 2014, **90:**1003-1013.

19.	Lutomiah J, Barrera R, Makio A, Mutisya J, Koka H, Owaka S, Koskei E, Nyunja A, Eyase F, Coldren R, Sang R: **Dengue Outbreak in Mombasa City, Kenya, 2013-2014: Entomologic Investigations.** *PLoS Negl Trop Dis* 2016, **10:**e0004981.

20.	Mboera LE, Mweya CN, Rumisha SF, Tungu PK, Stanley G, Makange MR, Misinzo G, De Nardo P, Vairo F, Oriyo NM: **The Risk of Dengue Virus Transmission in Dar es Salaam, Tanzania during an Epidemic Period of 2014.** *PLoS Negl Trop Dis* 2016, **10:**e0004313.

21.	Kajeguka D, Kaaya R, Desrochers R, Iranpour M, Kavishe R, Mwakalinga S, Schioler K, Alifrangis M, Lindsay L, Dibernardo A, et al: **Mapping clusters of chikungunya and dengue transmission in northern Tanzania using disease exposure and vector data.** *Tanzania Journal of Health Research* 2017, **19 (4)**.

22.	Iwashita H, Higa Y, Futami K, Lutiali PA, Njenga SM, Nabeshima T, Minakawa N: **Mosquito arbovirus survey in selected areas of Kenya: detection of insect-specific virus.** *Trop Med Health* 2018, **46:**19.

23.	Kone B, Konan L, Fofana D, Koffi A, Coulibaly D, Benie J: **Entomological evaluation of the risk of spread ofthe dengue 3 epidemic in the health district ofCocody (Abidjan, Côte d’Ivoire).** *International Journal of Mosquito Research* 2018, **5 (5):**19-24.

24.	Ndiaye EH, Diallo D, Fall G, Ba Y, Faye O, Dia I, Diallo M: **Arboviruses isolated from the Barkedji mosquito-based surveillance system, 2012-2013.** *BMC Infect Dis* 2018, **18:**642.

25.	Selhorst P, Makiala-Mandanda S, De Smet B, Mariën J, Anthony C, Binene-Mbuka G, De Weggheleire A, Ilombe G, Kinganda-Lusamaki E, Pukuta-Simbu E, et al: **Molecular characterization of chikungunya virus during the 2019 outbreak in the Democratic Republic of the Congo.** *Emerg Microbes Infect* 2020, **9:**1912-1918.

26.	Heath CJ, Grossi-Soyster EN, Ndenga BA, Mutuku FM, Sahoo MK, Ngugi HN, Mbakaya JO, Siema P, Kitron U, Zahiri N, et al: **Evidence of transovarial transmission of Chikungunya and Dengue viruses in field-caught mosquitoes in Kenya.** *PLoS Negl Trop Dis* 2020, **14:**e0008362.

27.	Diallo D, Fall G, Diagne CT, Gaye A, Ba Y, Dia I, Faye O, Diallo M: **Concurrent amplification of Zika, chikungunya, and yellow fever virus in a sylvatic focus of arboviruses in Southeastern Senegal, 2015.** *BMC Microbiol* 2020, **20:**181.

28.	Diouf B, Gaye A, Diagne CT, Diallo M, Diallo D: **Zika virus in southeastern Senegal: survival of the vectors and the virus during the dry season.** *BMC Infect Dis* 2020, **20:**371.

29.	De Weggheleire A, Nkuba-Ndaye A, Mbala-Kingebeni P, Mariën J, Kindombe-Luzolo E, Ilombe G, Mangala-Sonzi D, Binene-Mbuka G, De Smet B, Vogt F, et al: **A Multidisciplinary Investigation of the First Chikungunya Virus Outbreak in Matadi in the Democratic Republic of the Congo.** *Viruses* 2021, **13**.

30.	Chiuya T, Masiga DK, Falzon LC, Bastos ADS, Fèvre EM, Villinger J: **A survey of mosquito-borne and insect-specific viruses in hospitals and livestock markets in western Kenya.** *PLoS One* 2021, **16:**e0252369.

31.	Ngingo BL, Mboera LEG, Chengula A, Machelle I, Makange MR, Msolla M, Mwanyika GO, Rugarabamu S, Misinzo G: **Aedes aegypti abundance, larval indices and risk for dengue virus transmission in Kinondoni district, Tanzania.** *Trop Med Health* 2022, **50:**1.

32.	Tchibozo C, Hounkanrin G, Yadouleton A, Bialonski A, Agboli E, Lühken R, Schmidt-Chanasit J, Jöst H: **Surveillance of arthropod-borne viruses in Benin, West Africa 2020-2021: detection of dengue virus 3 in Aedes aegypti (Diptera: Culicidae).** *Mil Med Res* 2022, **9:**64.

33.	El-Kady AM, Osman HA, Alemam MF, Marghani D, Shanawaz MA, Wakid MH, Al-Megrin WAI, Elshabrawy HA, Abdella OH, Allemailem KS, et al: **Circulation of Dengue Virus Serotype 2 in Humans and Mosquitoes During an Outbreak in El Quseir City, Egypt.** *Infect Drug Resist* 2022, **15:**2713-2721.
