# Supplementary material for: Building resilience against the growing threat of arboviruses II: a scoping review of Aedes vector competence, larval and adult bionomics and species distribution in Africa
Source: Parasit Vectors. 2026 Jul 28;19:460. doi: 10.1186/s13071-026-07552-5 (PMC13693134; doi:10.1186/s13071-026-07552-5)
Supplement: Supplementary file 3 [file 13071_2026_7552_MOESM3_ESM.docx]

| **Supplementary Table S2.1. Bionomics- *Aedes* Adult Traps Publications** | | | | | | |
| --- | --- | --- | --- | --- | --- | --- |
| Number | Study | Publication Year | Country | Adult trap type | Mosquito Species | Ref |
| 1 | Linthicum et al. | 1985 | Kenya | CDC miniature light trap | *Aedes cumminsii, Ae. lineatopennis, Ae. circumluteolus, Ae. sudanensis, Ae. dentatus, Ae. quasiunivittatus* | [1] |
| 2 | Merlin et al. | 1986 | Rep. of Congo | larval collection, ovitraps, human landing catch | *Aedes aegypti* | [2] |
| 3 | Birley et al. | 1987 | Kenya | Human Landing Catch (HLC) | *Aedes aegypti* | [3] |
| 4 | Irving-Bell et al. | 1987 | Nigeria | Other (describe in next column) | *Aedes aegypti, Ae. vittatus* | [4] |
| 5 | Sabanitelli et al. | 1988 | Comoros | Human Landing Catch (HLC) | *Aedes aegypti* | [5] |
| 6 | Fontenille et al. | 1988 | Madagascar | Human Landing Catch (HLC) | *Aedes albopictus, Ae. moucheti/lambrechti, Ae. albocephalus, Ae. aegypti, Ae. vittatus, Ae. madagascarensis, Ae. tiptoni* | [6] |
| 7 | Lindsay et al. | 1989 | Gambia | Human Landing Catch (HLC) | *Aedes luteocephalus, Ae. simpsoni, Ae. longipalpis, Ae. irritans, Ae. furcifer/taylor* | [7] |
| 8 | Nasidi et al. | 1989 | Nigeria | Larval and pupal survey collection | *Aedes aegypti* | [8] |
| 9 | Beier et al. | 1990 | Kenya | Multiple (details in next column) | *Aedes aegypti, Ae. ochraceus, Ae. mcintoshi* | [9] |
| 10 | Mathiot et al. | 1990 | Central African Republic | Human Landing Catch (HLC) | *Aedes aegypti, Ae. africanus, Ae. argenteopunctatus, Ae. circumluteolus, Ae. cumminsi, Ae. dendrophilus, Ae.* | [10] |
| 11 | Kemp et al. | 1991 | South Africa | Ovitrap/oviposition trap | *Aedes aegypti, Ae. demeilloni, Ae. dendrophilus, Ae. simpsoni, Ae. strelitziae.* | [11] |
| 12 | Nwoke et al. | 1993 | Nigeria | Other (describe in next column) | *Aedes aegypti, Ae. aegypti and Ae. vittatus* | [12] |
| 13 | Karch et al. | 1993 | DRC | Multiple (details in next column) | *Aedes aegypti, Ae. argenteopunctus, Ae. faricanus, Ae. simpsoni, Ae vittatus* | [13] |
| 14 | Lutwama et al. | 1994 | Uganda | Human Landing Catch (HLC) | *Aedes bromeliae/Aedes simpsoni* | [14] |
| 15 | Karch et al. | 1995 | Democratic Republic of Congo | Human Landing Catch (HLC) | *Aedes aegypti* | [15] |
| 16 | Fontenille et al. | 1995 | Senegal | Multiple (details in next column) | *Aedes mcintoshi, Ae. ochraceus, Ae. fowleri, Ae. dalzieli,* | [16] |
| 17 | Traore-Lamizana et al. | 1996 | Senegal | Human Landing Catch (HLC) | *Aedes furcifer, Ae. taylori, Ae. luteocephalus* | [17] |
| 18 | Diarrassouba et al. | 1997 | Cote d'Ivoire | Human Landing Catch (HLC) | *Aedes aegypti, Ae. fowleri* | [18] |
| 19 | Thonnon et al. | 1998 | Senegal | Human Landing Catch (HLC) | *Aedes aegypti, Ae. luteocephalus, Ae. metallicus.* | [19] |
| 20 | Thonnon et al. | 1999 | Senegal | CDC miniature light trap | *Aedes aegypti* | [20] |
| 21 | Diarrassouba et al. | 2000 | Cote d'Ivoire | Human Landing Catch (HLC) | *Aedes aegypti, Ae. furcifer, Ae. africanus, Ae. tarsalis* | [21] |
| 22 | Akoua-Koffi et al. | 2001 | Cote d'Ivoire | Multiple (details in next column) | *Aedes aegypti* | [22] |
| 23 | Fontenille et al. | 2001 | Cameroon | Multiple (details in next column) | *Aedes albopictus* | [23] |
| 24 | Mostafa et al. | 2002 | Egypt | Multiple (details in next column) | *Ae. caspius, Ae.detritus* | [24] |
| 25 | Miyingo-Kezimbira et al. | 2003 | Uganda | Multiple (details in next column) | *Aedes bromeliae/Aedes simpsoni* | [25] |
| 26 | Okogun et al. | 2003 | Nigeria |  |  | [26] |
| 27 | Appawu et al. | 2006 | Ghana | Larval and pupal survey collection | *Aedes mosquitoes - species not specified* | [27] |
| 28 | Doannio et al. | 2006 | Cote d'Ivoire |  | *Aedes aegypti, Ae. furcifer, Ae vittatus, Ae palpalis,* | [28] |
| 29 | Ba et al. | 2006 | Senegal | BG Sentinel | *Aedes aegypti* | [29] |
| 30 | Faye et al. | 2007 | Senegal | Human Landing Catch (HLC) | *Aedes furcifer, Ae. aegypti, Ae. luteocephalus, Ae. taylori, Ae. metallicus.* | [30] |
| 31 | Sang et al. | 2008 | Comoros | Human Landing Catch (HLC) | *Aedes aegypti, Ae. simpsoni complex* | [31] |
| 32 | Gould et al. | 2008 | Sudan | Larval and pupal survey collection | *Aedes aegypti, Aedes luteocephalus* | [32] |
| 33 | Muturi et al. | 2008 | Kenya | Multiple (details in next column) | *Aedes cumminsii, Ae. taylori* | [33] |
| 34 | Oguoma et al. | 2008 | Nigeria | Multiple (details in next column) | *Ae. albopictus, Ae. aegypti, Ae. africanus* | [34] |
| 35 | Onyido et al. | 2008 | Nigeria | Multiple (details in next column) | *Aedes aegypti, A. aegypti, A. africanus, A. vittatus, A. luteocephalus.* | [35] |
| 36 | Lacroix et al. | 2009 | Reunion | Ovitrap/oviposition trap | *Aedes albopictus* | [36] |
| 37 | Onyido et al. | 2010 | Nigeria | Ovitrap/oviposition trap | *Aedes aegypti* | [37] |
| 38 | Yaro et al. | 2010 | Burkina Faso | larval collection, human landing catch | *Aedes aegypti* | [38] |
| 39 | Aribodor et al. | 2011 | Nigeria | Resting collection | *Aedes aegypti* | [39] |
| 40 | Konan et al. | 2011 | Cote d'Ivoire | Larval and pupal survey collection | *Aedes aegypti, Ae. opok, Ae. luteocephalus, Ae. apicoargentus, Ae. dendrophilus* | [40] |
| 41 | Mwangangi et al. | 2012 | Kenya | Multiple (details in next column) | *Aedes aegypti, Ae. tricholabis, Ae. dalzieli, Ae. sudanensis* | [41] |
| 42 | Tchouassi et al. | 2012 | Kenya | CDC miniature light trap | *Ae. mcintoshi, Ae. ochraceus, Ae. sudanensis, Ae. tricholabis, Ae. furcifer, Ae. hirsutus.* | [42] |
| 43 | Mutebi et al. | 2012 | Uganda | CDC miniature light trap | *Aedes aegypti, Ae. albocephalus, Ae. argenteopunctuatus, Ae. domesticus, Ae. hirsutus, Ae. leptolabis, Ae.* | [43] |
| 44 | Kamgang et al. | 2012 | Cameroon | Mouth aspirator | *Aedes albopictus, Ae. aegypti* | [44] |
| 45 | Raharimalala et al. | 2012 | Madagascar | Multiple (details in next column) | *Aedes aegypti, Ae. albopictus* | [45] |
| 46 | Crabtree et al. | 2013 | Uganda | CDC backpack | *Aedes cumminsii, Ae. tarsalis* | [46] |
| 47 | Diallo et al. | 2013 | Senegal | CDC backpack | *Aedes aegypti, Ae. luteocephalus, Ae. vittatus, Ae. taylori, Ae. furcifer, Ae. dalzieli, Ae. argenteopunctatus* | [47] |
| 48 | Lutomiah et al. | 2013 | Kenya | Multiple (details in next column) | *Aedes mcintoshi, Ae. circumluteolus, Ae. tricholabis, Ae. ochraceus, Ae. sudanensis, Ae. cumminsi, Ae.* | [48] |
| 49 | Diallo et al. | 2013 | Senegal | Human Landing Catch (HLC) | *Aedes aegypti, Ae. furcifer, Ae. vittatus* | [49] |
| 50 | Ebenezer et al. | 2013 | Nigeria | Resting collection | *Aedes aegypti* | [50] |
| 51 | Kone et al. | 2013 | Côte d’Ivoire | Ovitrap/oviposition trap, larval collection; Larval and pupal survey collection | *Aedes aegypti, Ae. opok, Ae. luteocephalus* | [51] |
| 52 | Krajacich et al. | 2014 | Senegal | Other (describe in next column) | *Aedes aegypti, Ae. vittatus* | [52] |
| 53 | Yadouleton et al. | 2014 | Benin | Human Landing Catch (HLC) | *Ae. aegypti* | [53] |
| 54 | Konan et al. | 2014 | Côte d’Ivoire | larval collection, ovitraps,IRS, human landing catch; Multiple (details in next column) | *Aedes aegypti, Ae. africanus, Ae. luteocephalus , Ae. vittatus, Ae. opok, Ae. apicoargenteus, Ae. dendrophillus, Ae. haworti, Ae. fraseri, Ae. unillineatus; Ae. apicoargenteus, Ae. dendrophilus, Ae. haworthi, Ae. fraseri, Ae. opok, Ae. taeniarostris, Ae. unilineatus, Ae. africanus, Ae.mutilus, Ae. argenteo-punctatus, Ae. vittatus, Ae. luteocephalus* | [54] |
| 55 | Masaninga et al. | 2014 | Zambia | Multiple (details in next column) | *Aedes aegypti, Ae. mutilus, Ae. minutis* | [55] |
| 56 | Diallo et al. | 2014 | Senegal | Human Landing Catch (HLC) | *Aedes furcifer Ae. furcifer, Ae. luteocephalus, Ae. taylori, Ae. africanus, Ae. centropunctatus, Ae. dalzieli, Ae. mcintoshi, Ae. vitattus* | [56] |
| 57 | Yalwala et al. | 2015 | Kenya | Larval and pupal survey collection | *Aedes aegypti, Ae. aegypti formosus* | [57] |
| 58 | Gouagna et al. | 2015 | Reunion | BG Sentinel | *Aedes albopictus* | [58] |
| 59 | Irish et al. | 2015 | Tanzania | Gravid Aedes trap (GAT) | *Aedes aegypti, Ae. pembaensis* | [59] |
| 60 | Mweya et al. | 2015 | Tanzania | Multiple (details in next column) | *Aedes aegypti* | [60] |
| 61 | Kaddumukasa et al. | 2015 | Uganda | CDC miniature light trap | *Aedes africanus, A. africanus, A. coustani, A. implexus,* | [61] |
| 62 | Omondi et al. | 2015 | Kenya | Multiple (details in next column) |  | [62] |
| 63 | Oduola et al. | 2016 | Nigeria | Resting collection | *Aedes aegypti* | [63] |
| 64 | Suzuki-Takashi et al. | 2016 | Ghana | Human Landing Catch (HLC) | *Aedes aegypti, Ae. vittatus, Ae. albopictus, Ae. granti.* | [64] |
| 65 | Goff et al. | 2016 | Reunion | BG Sentinel | *Aedes albopictus* | [65] |
| 66 | Lutomiah et al. | 2016 | Kenya | BG Sentinel | *Aedes aegypti, Ae. tricholabis* | [66] |
| 67 | Mboera et al. | 2016 | Tanzania | Larval and pupal survey collection | *Aedes aegypti* | [67] |
| 68 | Muller et al. | 2016 | Mali | Multiple (details in next column) | *Aedes albopictus* | [68] |
| 69 | Arum et al. | 2016 | Kenya | CDC miniature light trap | *Aedes mcintoshi, Ae. ochraceus, Ae. sudanensis* | [69] |
| 70 | Ngugi et al. | 2017 | Kenya | Multiple (details in next column) | *Aedes aegypti* | [70] |
| 71 | Ndenga et al. | 2017 | Kenya | Multiple (details in next column) | *Aedes aegypti, Ae. simpsoni* | [71] |
| 72 | Agha et al. | 2017 | Kenya | BG Sentinel | *Aedes aegypti* | [72] |
| 73 | Kajeguka et al. | 2017 | Tanzania | Other (describe in next column) | *Aedes aegypti* | [73] |
| 74 | Mohamed et al. | 2017 | Sudan | Multiple (details in next column) | *Ae. caspius, Ae. vexans, Ae. quasiunivittatus, Ae. dentatus* | [74] |
| 75 | Zahouli et al. | 2017 | Cote d'Ivoire | Multiple (details in next column) | *Ae. aegypti, Ae. africanus Ae. dendrophilus Ae. furcifer and Ae. vittatus* | [75] |
| 76 | Guindo-Coulibaly et al. | 2018 | Cote d'Ivoire | Other (describe in next column) | *Aedes aegypti* | [76] |
| 77 | Chepkorir et al. | 2018 | Kenya | BG Sentinel | *Aedes aegypti* | [77] |
| 78 | Iwashita et al. | 2018 | Kenya | CDC miniature light trap | *Aedes aegypti* | [78] |
| 79 | Kone et al. | 2018 | Cote d'Ivoire | Human-baited double net trap | *Aedes aegypti* | [79] |
| 80 | Mugabe et al. | 2018 | Mozambique | Ovitrap/oviposition trap | *Aedes aegypti* | [80] |
| 81 | Ndiaye et al. | 2018 | Senegal | CDC miniature light trap | *Aedes ochraceus, Ae. aegypti, Ae. dalzielii, Ae. vexans, Ae. sudanensis, Ae. minutus, Ae. furcifer, Ae. argentopunctatus.* | [81] |
| 82 | Mutebi et al. | 2018 | Uganda | CDC miniature light trap | *Aedes aegypti* | [82] |
| 83 | Delatte et al. | 2018 | Reunion | Multiple (details in next column) | *Aedes albopictus* | [83] |
| 84 | Aikpon et al. | 2019 | Benin | Human Landing Catch (HLC) | *Aedes aegypti* | [84] |
| 85 | Sissoko et al. | 2019 | Mali | Sticky trap | *Aedes aegypti* | [85] |
| 86 | Fofana et al. | 2019 | Cote d'Ivoire | Larval and pupal survey collection | *Aedes aegypti* | [86] |
| 87 | Gneme et al. | 2019 | Burkina Faso | CDC miniature light trap | *Aedes aegypti* | [87] |
| 88 | Gorsich et al. | 2019 | South Africa | CDC miniature light trap | *Aedes aegypti, Ae. aerarius, Ae. dentatus complex, Ae. mcintoshi, Ae. metallicus, Ae. ochraceus, Ae.* | [88] |
| 89 | Mulchandani et al. | 2019 | Ethiopia | Larval and pupal survey collection | *Ae. simpsoni* | [89] |
| 90 | Iyaloo et al. | 2019 | Mauritius | Ovitrap/oviposition trap | *Aedes albopictus* | [90] |
| 91 | Labbo et al. | 2019 | Niger | Human Landing Catch (HLC) | *Aedes aegypti, Ae. vittatus, Aedes luteocephalus* | [91] |
| 92 | Karuitha et al. | 2019 | Kenya | Multiple (details in next column) | *Aedes aegypti, Ae simpsoni, Ae chausseri* | [92] |
| 93 | Nebinur et al. | 2019 | Eritrea | Larval and pupal survey collection | *Aedes aegypti* | [93] |
| 94 | Thabet et al. | 2019 | Togo | Multiple (details in next column) | *Ae. aegypti, Ae. Africanus, Ae. vittatus* | [94] |
| 95 | Agha et al. | 2019 | Kenya | Multiple (details in next column) | *Aedes aegypti* | [95] |
| 96 | Agwu et al. | 2019 | Nigeria | Multiple (details in next column) | *Aedes aegypti, Ae. aegypti, Ae. africanus, Ae. albopictus, Ae. cumminsii, Ae. luteocephalus, Ae. vittatus.* | [96] |
| 97 | Nwachukwu et al. | 2020 | Nigeria | CDC miniature light trap | *Aedes aegypti, Ae. africanus and Ae. luteocephalus.* | [97] |
| 98 | Philbert et al. | 2020 | Tanzania | Other (describe in next column) | *Aedes aegypti* | [98] |
| 99 | Diallo et al. | 2020 | Senegal | CDC backpack | *Aedes aegypti, Ae. vittatus, Ae. furcifer, Ae. Luteocephalus, Ae. Argenteopunctatus* | [99] |
| 100 | Namountougou et al. | 2020 | Burkina Faso | Larval and pupal survey collection | *Aedes aegypti* | [100] |
| 101 | Eastwood et al. | 2020 | Kenya | BG Sentinel | *Aedes aegypti, Ae. dentatus, Ae. tricholabis, Aedes chaussieri, Ae. cumminsi, Ae. mcintoshi* | [101] |
| 102 | Johnson et al. | 2020 | South Africa | CDC miniature light trap | *Aedes aegypti, Ae. aegypti, Ae dentatus group,* | [102] |
| 103 | Irikannu et al. | 2020 | Nigeria | Multiple (details in next column) | *Aedes aegypti, Ae albopictus* | [103] |
| 104 | Abilio et al. | 2020 | Mozambique | Ovitrap/oviposition trap | *Aedes luteocephalus, Ae. aegypti* | [104] |
| 105 | Diallo et al. | 2020 | Senegal | Human Landing Catch (HLC) | *Aedes furcifer* | [105] |
| 106 | Rose et al. | 2020 | Senegal | Ovitrap/oviposition trap | *Aedes aegypti* | [106] |
| 107 | Captain-Esoah et al. | 2020 | Ghana | Human Landing Catch (HLC) | *Aedes aegypti, Ae. vittatus* | [107] |
| 108 | Mayi et al. | 2020 | Cameroon | Multiple (details in next column) | *Aedes aegypti, Ae. albopictus, Ae. africanus, Ae. argenteopunctatus, Ae. simpsoni, Ae. domesticus, Ae.* | [108] |
| 109 | Fagbohun et al. | 2020 | Nigeria | Multiple (details in next column) | *Aedes aegypti, Ae. albopictus* | [109] |
| 110 | Musa et al. | 2020 | Kenya | Multiple (details in next column) | *Aedes aegypti, Ae. aegypti, Ae. metallicus, Ae. simpsoni, Ae. tarsalis, Ae. tricholabis* | [110] |
| 111 | Kampango et al. | 2021 | Tanzania | Larval and pupal survey collection | *Aedes aegypti, Ae. Bromeliae/Ae. lillie, Ae. metallicus* | [111] |
| 112 | Waldetensai et al. | 2021 | Ethiopia | Larval and pupal survey collection | *Aedes mosquitoes - species not specified* | [112] |
| 113 | Onwuzulike et al. | 2021 | Nigeria | Larval and pupal survey collection | *Aedes albopictus, Ae. aegypti, Ae. bromeliae* | [113] |
| 114 | Diouf et al. | 2021 | Senegal | CDC backpack | *Ae. aegypti formosus and Ae. aegypti aegypti subspecies* | [114] |
| 115 | Diouf et al. | 2021 | Senegal | BG Sentinel | *Aedes aegypti* | [115] |
| 116 | Gutu et al. | 2021 | Ethiopia | Larval and pupal survey collection | *Aedes aegypti, Ae. africanus* | [116] |
| 117 | Kpan et al. | 2021 | Cote d'Ivoire | Ovitrap/oviposition trap | *Aedes aegypti* | [117] |
| 118 | Hamaidia et al. | 2021 | Algeria | Multiple (details in next column) | *Aedes albopictus* | [118] |
| 119 | Chiuya et al. | 2021 | Kenya | Multiple (details in next column) | *Aedes aegypti, Ae. aegypti, Ae. africanus, Ae. hirsutus, Ae. micintoshi, Ae. simpsoni, Ae. tricholabis ,Ae.* | [119] |
| 120 | Guarido et al. | 2021 | South Africa | Multiple (details in next column) |  | [120] |
| 121 | Irikannu et al. | 2021 | Nigeria | Multiple (details in next column) | *Aedes aegypti and Ae. albopictus.* | [121] |
| 122 | Karisa et al. | 2021 | Kenya | Multiple (details in next column) | *Aedes aegypti* | [122] |
| 123 | Ngingo et al. | 2022 | Tanzania | Multiple (details in next column) | *Aedes aegypti* | [123] |
| 124 | Owusu-Asenso et al. | 2022 | Ghana | BG Sentinel | *Aedes aegypti, Ae. africanus and Ae. luteocephalus* | [124] |
| 125 | Kouadio et al. | 2022 | Cote d'Ivoire | Gravid Aedes trap (GAT) | *Aedes aegypti, Ae. luteocephalus, Ae. unilineatus* | [125] |
| 126 | Djiappi-Tchamen et al. | 2022 | Cameroon | BG Sentinel | *Aedes albopictus, Ae. aegypti, Ae. contigus, Ae. simpsoni* | [126] |
| 127 | Badolo et al. | 2022 | Burkina Faso | Multiple (details in next column) | *Aedes aegypti, Ae. vittatus, Ae. hirsutus, Ae. metallicus* | [127] |
| 128 | Sene et al. | 2022 | Senegal | BG Sentinel | *Aedes aegypti* | [128] |
| 129 | Stoek et al. | 2022 | Mauritania | Other (describe in next column) |  | [129] |
| 130 | Kamau et al. | 2022 | Kenya | CDC miniature light trap | *Aedes bromeliae/Aedes simpsoni* | [130] |
| 131 | Ndenga et al. | 2022 | Kenya |  | *Aedes aegypti* | [131] |

**References**

1.	Linthicum KJ, Kaburia HF, Davies FG, Lindqvist KJ: **A blood meal analysis of engorged mosquitoes found in Rift Valley fever epizootics area in Kenya.** *J Am Mosq Control Assoc* 1985, **1:**93-95.

2.	Merlin M, Josse R, Kouka-Bemba D, Meunier D, Senga J, Simonkovich E, Malonga JR, Manoukou F, Georges AJ: **[Evaluation of immunological and entomotological indices of yellow fever in Pointe-Noire, People's Republic of Congo].** *Bull Soc Pathol Exot Filiales* 1986, **79:**199-206.

3.	Birley MH, Mutero CM, Turner IF, Chadwick PR: **The effectiveness of mosquito coils containing esbiothrin under laboratory and field conditions.** *Ann Trop Med Parasitol* 1987, **81:**163-171.

4.	Irving-Bell RJ, Okoli EI, Diyelong DY, Lyimo EO, Onyia OC: **Septic tank mosquitoes: competition between species in central Nigeria.** *Med Vet Entomol* 1987, **1:**243-250.

5.	Sabanitelli G, Petrarca V, Bagalino S: **Notes on the vectors of malaria and Bancroftian filariasis on the islands of Grand Comoros and Moheli (Comoros).** *Bull Soc Pathol Exot Filiales* 1988, **82**.

6.	Fontenille D, Mathiot C, Rodhain F, Coulanges P: **[Arbovirus infections on the island of Nosy-Be; serologic and entomologic findings].** *Arch Inst Pasteur Madagascar* 1988, **54:**101-115.

7.	Lindsay SW, Janneh LM: **Preliminary field trials of personal protection against mosquitoes in The Gambia using deet or permethrin in soap, compared with other methods.** *Med Vet Entomol* 1989, **3:**97-100.

8.	Nasidi A, Monath TP, DeCock K, Tomori O, Cordellier R, Olaleye OD, Harry TO, Adeniyi JA, Sorungbe AO, Ajose-Coker AO: **Urban yellow fever epidemic in western Nigeria, 1987.** *Trans R Soc Trop Med Hyg* 1989, **83:**401-406.

9.	Beier JC, Odago WO, Onyango FK, Asiago CM, Koech DK, Roberts CR: **Relative abundance and blood feeding behavior of nocturnally active culicine mosquitoes in western Kenya.** *J Am Mosq Control Assoc* 1990, **6:**207-212.

10.	Mathiot CC, Grimaud G, Garry P, Bouquety JC, Mada A, Daguisy AM, Georges AJ: **An outbreak of human Semliki Forest virus infections in Central African Republic.** *Am J Trop Med Hyg* 1990, **42:**386-393.

11.	Kemp A, Jupp PG: **Potential for dengue in South Africa: mosquito ecology with particular reference to Aedes aegypti.** *J Am Mosq Control Assoc* 1991, **7:**574-583.

12.	Nwoke BEB, Nduka FO, Okereke OM, Ehighibe OC: **Sustainable urban development and human health: septic tank as a major breeding habitat of mosquito vectors of human diseases in south-eastern Nigeria.** *Applied Parasitology* 1993, **34:**1-10.

13.	Karch S, Asidi N, Manzambi ZM, Salaun JJ: **[The culicidian fauna and its nuisance in Kinshasha (Zaire)].** *Bull Soc Pathol Exot* 1993, **86:**68-75.

14.	Lutwama J, Mukwaya L: **Mark-release-recapture studies on three anthropophilic populations of  Aedes ( Stegomyia )  simpsoni  comple(Diptera: Culicidae) in Uganda.** *Bulletin of Entomological Research* 1994, **84**.

15.	Karch S, Asidi N, Manzambi Z, Salaun JJ, Mouchet J: **Impact of deltamethrin-impregnated bednets on biting rates of mosquitoes in Zaire.** *J Am Mosq Control Assoc* 1995, **11:**191-194.

16.	Fontenille D, Traore-Lamizana M, Zeller H, Mondo M, Diallo M, Digoutte JP: **Short report: Rift Valley fever in western Africa: isolations from Aedes mosquitoes during an interepizootic period.** *Am J Trop Med Hyg* 1995, **52:**403-404.

17.	Traoré-Lamizana M, Fontenille D, Zeller HG, Mondo M, Diallo M, Adam F, Eyraud M, Maiga A, Digoutte JP: **Surveillance for yellow fever virus in eastern Senegal during 1993.** *J Med Entomol* 1996, **33:**760-765.

18.	Diarrassouba S, Dossou-Yovo J: **Rythme d'activité atypique chez *Aedes aegypti* en zone de savane sub-soudanienne de Côte d'Ivoire.** *Bulletin de la Societe de pathologie exotique* 1997, **90**.

19.	Thonnon J, Spiegel A, Diallo M, Sylla R, Fall A, Mondo M, Fontenille D: **Yellow fever outbreak in Kaffrine, Senegal 1996: epidemiological and entomological findings.** *Trop Med Int Health* 1998, **3:**872-877.

20.	Thonnon J, Spiegel A, Diallo M, Diallo A, Fontenille D: **[Chikungunya virus outbreak in Senegal in 1996 and 1997].** *Bull Soc Pathol Exot* 1999, **92:**79-82.

21.	Diarrassouba S, Herve J, Dossou-Yovo J, Carnevale P: **Water stocking habits and seasonal variations in biting rates of Aedes (Stegomyia) aegypti Linnaeus (Diptera: Culicidae) in a sub-sudanian village of Cote d'Ivoire.** *Research and reviews in parasitology* 2000.

22.	Akoua-Koffi C, Diarrassouba S, Bénié VB, Ngbichi JM, Bozoua T, Bosson A, Akran V, Carnevale P, Ehouman A: **[Investigation surrounding a fatal case of yellow fever in Côte d'Ivoire in 1999].** *Bull Soc Pathol Exot* 2001, **94:**227-230.

23.	Fontenille D, Toto JC: **Aedes (Stegomyia) albopictus (Skuse), a potential new Dengue vector in southern Cameroon.** *Emerg Infect Dis* 2001, **7:**1066-1067.

24.	Mostafa AA, Allam KA, Osman MZ: **Mosquito species and their densities in some Egyptian governorates.** *J Egypt Soc Parasitol* 2002, **32:**9-20.

25.	Miyingo-Kezimbira A, Lutwama J: **Morphometrics of Larvae of *Aedes (Stegomyia) simpsoni*Complex (Diptera: Culicidae) in Uganda.** *International Journal of Tropical Insect Science* 2003, **23**.

26.	Okogun GR, Nwoke B, Okere A, Anosike J, Esekhegbe A: **Epidemiological implications of preferences of breeding sites of mosquito species in Midwestern Nigeria.** *Ann Agric Environ Med* 2003, **10:**217-222.

27.	Appawu M, Dadzie S, Abdul H, Asmah H, Boakye D, Wilson M, Ofori-Adjei D: **Surveillance of viral haemorrhagic fevers in ghana: entomological assessment of the risk of transmission in the northern regions.** *Ghana Med J* 2006, **40:**137-141.

28.	Doannio JM, Dossou-Yovo J, Diarrassouba S, Rakotondraibé ME, Chauvancy G, Rivière F: **[Comparison of specific composition and mosquitoes population dynamics in two villages of the centre of Côte-d'Ivoire, with and without irrigated rice growing area].** *Bull Soc Pathol Exot* 2006, **99:**204-206.

29.	Ba Y, Diallo D, Dia I, Diallo M: **[Feeding pattern of Rift Valley Fever virus vectors in Senegal. Implications in the disease epidemiology].** *Bull Soc Pathol Exot* 2006, **99:**283-289.

30.	Faye O, Diallo M, Dia I, Ba Y, Mondo M, Sylla R, Faye PC, Sall AA: **[Integrated approach to yellow fever surveillance: pilot study in Senegal in 2003-2004].** *Bull Soc Pathol Exot* 2007, **100:**187-192.

31.	Sang RC, Ahmed O, Faye O, Kelly CL, Yahaya AA, Mmadi I, Toilibou A, Sergon K, Brown J, Agata N, et al: **Entomologic investigations of a chikungunya virus epidemic in the Union of the Comoros, 2005.** *Am J Trop Med Hyg* 2008, **78:**77-82.

32.	Gould LH, Osman MS, Farnon EC, Griffith KS, Godsey MS, Karch S, Mulenda B, El Kholy A, Grandesso F, de Radiguès X, et al: **An outbreak of yellow fever with concurrent chikungunya virus transmission in South Kordofan, Sudan, 2005.** *Trans R Soc Trop Med Hyg* 2008, **102:**1247-1254.

33.	Muturi EJ, Muriu S, Shililu J, Mwangangi JM, Jacob BG, Mbogo C, Githure J, Novak RJ: **Blood-feeding patterns of Culex quinquefasciatus and other culicines and implications for disease transmission in Mwea rice scheme, Kenya.** *Parasitol Res* 2008, **102:**1329-1335.

34.	Oguoma V, Ikpeze O: **Species composition and abundance of mosquitoes of a tropical irrigation ecosystem.** *Animal Research International* 2008.

35.	Onyido A, Ozumba N, Ezike V, Chukwuekezie O, Nwosu E, Nwaorgu O, Ikpeze O: **Mosquito fauna of a tropical museum and zoological garden complex.** *Animal Research International* 2008, **5 (2)**.

36.	Lacroix R, Delatte H, Hue T, Reiter P: **Dispersal and survival of male and female Aedes albopictus (Diptera: Culicidae) on Réunion Island.** *J Med Entomol* 2009, **46:**1117-1124.

37.	Onyido A, Ozumba N, Ezike V, Nwosu E, Chukwuekezie O, Amadi E: **Surveillance studies of Aedes Stegomyia mosquitoes in three ecological locations of Enugu, South-eastern Nigeria.** *Internet Journal of Infectious Diseases* 2010, **8**.

38.	Yaro S, Zango A, Rouamba J, Diabaté A, Dabiré R, Kambiré C, Tiendrebeogo SM, Yonli T, Ouango JG, Diagbouga SP: **[Epidemiological situation of yellow fever in Burkina Faso from 2003 to 2008].** *Bull Soc Pathol Exot* 2010, **103:**44-47.

39.	Aribodor D, Ikpeze O, Onyido A, Okoye C: **Survey of indoor adult malaria vectors and challenges of using long lasting insecticide-treated nets in malaria control in Awka-Etiti, Anambra State, Nigeria.** *Nigerian Journal of Parasitology* 2011.

40.	Konan YL, Fofana D, Coulibaly ZI, Diallo A, Koné AB, Doannio JM, Ekra KD, Odéhouri-Koudou P: **[Entomological investigations conducted around ten cases of yellow fever in 2009 in the Denguélé sanitary region, Côte-d'Ivoire].** *Bull Soc Pathol Exot* 2011, **104:**296-302.

41.	Mwangangi JM, Midega J, Kahindi S, Njoroge L, Nzovu J, Githure J, Mbogo CM, Beier JC: **Mosquito species abundance and diversity in Malindi, Kenya and their potential implication in pathogen transmission.** *Parasitol Res* 2012, **110:**61-71.

42.	Tchouassi DP, Sang R, Sole CL, Bastos AD, Mithoefer K, Torto B: **Sheep skin odor improves trap captures of mosquito vectors of Rift Valley fever.** *PLoS Negl Trop Dis* 2012, **6:**e1879.

43.	Mutebi JP, Crabtree MB, Kading RC, Powers AM, Lutwama JJ, Miller BR: **Mosquitoes of western Uganda.** *J Med Entomol* 2012, **49:**1289-1306.

44.	Kamgang B, Nchoutpouen E, Simard F, Paupy C: **Notes on the blood-feeding behavior of Aedes albopictus (Diptera: Culicidae) in Cameroon.** *Parasit Vectors* 2012, **5:**57.

45.	Raharimalala FN, Ravaomanarivo LH, Ravelonandro P, Rafarasoa LS, Zouache K, Tran-Van V, Mousson L, Failloux AB, Hellard E, Moro CV, et al: **Biogeography of the two major arbovirus mosquito vectors, Aedes aegypti and Aedes albopictus (Diptera, Culicidae), in Madagascar.** *Parasit Vectors* 2012, **5:**56.

46.	Crabtree MB, Kading RC, Mutebi JP, Lutwama JJ, Miller BR: **Identification of host blood from engorged mosquitoes collected in western Uganda using cytochrome oxidase I gene sequences.** *J Wildl Dis* 2013, **49:**611-626.

47.	Diallo D, Chen R, Diagne CT, Ba Y, Dia I, Sall AA, Weaver SC, Diallo M: **Bloodfeeding patterns of sylvatic arbovirus vectors in southeastern Senegal.** *Trans R Soc Trop Med Hyg* 2013, **107:**200-203.

48.	Lutomiah J, Bast J, Clark J, Richardson J, Yalwala S, Oullo D, Mutisya J, Mulwa F, Musila L, Khamadi S, et al: **Abundance, diversity, and distribution of mosquito vectors in selected ecological regions of Kenya: public health implications.** *J Vector Ecol* 2013, **38:**134-142.

49.	Diallo M, Tall A, Dia I, Ba Y, Sarr FD, Ly A, Faye J, Badiane A, Diakhaby G, Faye P, et al: **Yellow fever outbreak in central part of Senegal 2002: epidemiological findings.** *Journal of Public Health and Epidemiology* 2013, **5-7:**291-296.

50.	Ebenezer A, Ben H, Enaregha E: **Atial distribution and indoor-resting density of mosquito species in the lowland rainforest of Bayelsa state, Nigeria.** *International Journal of Tropical Medicine* 2013.

51.	Kone AB, Konan YL, Coulibaly ZI, Fofana D, Guindo-Coulibaly N, Diallo M, Doannio JM, Ekra KD, Odehouri-Koudou P: **[Entomological evaluation of the risk of urban outbreak of yellow fever in 2008 in Abidjan, Côte d'Ivoire].** *Med Sante Trop* 2013, **23:**66-71.

52.	Krajacich BJ, Slade JR, Mulligan RT, Labrecque B, Kobylinski KC, Gray M, Kuklinski WS, Burton TA, Seaman JA, Sylla M, Foy BD: **Design and testing of a novel, protective human-baited tent trap for the collection of anthropophilic disease vectors.** *J Med Entomol* 2014, **51:**253-263.

53.	Yadouleton A, Agbanrin R, Vodounon C, Padonou G, Badirou K, Attolou R, Ursins F, Zola J, Allagbe H, Akogbeto M: **Seasonal distribution of Aedes aegypti in southern Benin: a risk of dengue virus transmission to urban populations.** *International Journal of Innovation and Applied Studies* 2014.

54.	Konan YL, Coulibaly ZI, Allali KB, Tétchi SM, Koné AB, Coulibaly D, Ekra KD, Doannio JM, Oudéhouri-Koudou P: **[Management of the yellow fever epidemic in 2010 in Séguéla (Côte d'Ivoire): value of multidisciplinary investigation].** *Sante Publique* 2014, **26:**859-867.

55.	Masaninga F, Muleba M, Masendu H, Songolo P, Mweene-Ndumba I, Mazaba-Liwewe ML, Kamuliwo M, Ameneshewa B, Siziya S, Babaniyi OA: **Distribution of yellow fever vectors in Northwestern and Western Provinces, Zambia.** *Asian Pac J Trop Med* 2014, **7S1:**S88-92.

56.	Diallo D, Sall AA, Diagne CT, Faye O, Hanley KA, Buenemann M, Ba Y, Weaver SC, Diallo M: **Patterns of a sylvatic yellow fever virus amplification in southeastern Senegal, 2010.** *Am J Trop Med Hyg* 2014, **90:**1003-1013.

57.	Yalwala S, Clark J, Oullo D, Ngonga D, Abuom D, Wanja E, Bast J: **Comparative efficacy of existing surveillance tools for Aedes aegypti in Western Kenya.** *J Vector Ecol* 2015, **40:**301-307.

58.	Gouagna LC, Dehecq JS, Fontenille D, Dumont Y, Boyer S: **Seasonal variation in size estimates of Aedes albopictus population based on standard mark-release-recapture experiments in an urban area on Reunion Island.** *Acta Trop* 2015, **143:**89-96.

59.	Irish SR, Batengana BM, Eiras AE, Cameron MM: **Evaluation of the AtrAedes™ Lure for Collection of Culex quinquefasciatus in Gravid Traps.** *J Am Mosq Control Assoc* 2015, **31:**107-109.

60.	Mweya CN, Kimera SI, Mellau LS, Mboera LE: **Inter-epidemic abundance and distribution of potential mosquito vectors for Rift Valley fever virus in Ngorongoro district, Tanzania.** *Glob Health Action* 2015, **8:**25929.

61.	Kaddumukasa M, Kayondo J, Masiga D, Akoi A, Lutwama J, Masembe C: **High proportion of mosquito vectors in Zika forest, Uganda, feeding on humans has implications for the spread of new arbovirus pathogens.** *African Journal of Biotechnology* 2015, **14**.

62.	Omondi D, Masiga DK, Ajamma YU, Fielding BC, Njoroge L, Villinger J: **Unraveling Host-Vector-Arbovirus Interactions by Two-Gene High Resolution Melting Mosquito Bloodmeal Analysis in a Kenyan Wildlife-Livestock Interface.** *PLoS One* 2015, **10:**e0134375.

63.	Oduola A, Obembe A, Adelaja O, Ande A: **Surveillance and insecticide susceptibility status of Culicine mosquitoes in selected communities utilizing long-lasting insecticidal nets in Kwara State, Nigeria.** *Animal Research International* 2016, **13**.

64.	Suzuki T, Osei JH, Sasaki A, Adimazoya M, Appawu M, Boakye D, Ohta N, Dadzie S: **Risk of transmission of viral haemorrhagic fevers and the insecticide susceptibilitystatus of.** *Ghana Med J* 2016, **50:**136-141.

65.	Le Goff G, Damiens D, Payet L, Ruttee AH, Jean F, Lebon C, Dehecq JS, Gouagna LC: **Enhancement of the BG-sentinel trap with varying number of mice for field sampling of male and female Aedes albopictus mosquitoes.** *Parasit Vectors* 2016, **9:**514.

66.	Lutomiah J, Barrera R, Makio A, Mutisya J, Koka H, Owaka S, Koskei E, Nyunja A, Eyase F, Coldren R, Sang R: **Dengue Outbreak in Mombasa City, Kenya, 2013-2014: Entomologic Investigations.** *PLoS Negl Trop Dis* 2016, **10:**e0004981.

67.	Mboera LE, Mweya CN, Rumisha SF, Tungu PK, Stanley G, Makange MR, Misinzo G, De Nardo P, Vairo F, Oriyo NM: **The Risk of Dengue Virus Transmission in Dar es Salaam, Tanzania during an Epidemic Period of 2014.** *PLoS Negl Trop Dis* 2016, **10:**e0004313.

68.	Müller GC, Tsabari O, Traore MM, Traore SF, Doumbia S, Kravchenko VD, Junnila A, Beier JC: **First record of Aedes albopictus in inland Africa along the River Niger in Bamako and Mopti, Mali.** *Acta Trop* 2016, **162:**245-247.

69.	Arum SO, Weldon CW, Orindi B, Tigoi C, Musili F, Landmann T, Tchouassi DP, Affognon HD, Sang R: **Plant resting site preferences and parity rates among the vectors of Rift Valley Fever in northeastern Kenya.** *Parasit Vectors* 2016, **9:**310.

70.	Ngugi HN, Mutuku FM, Ndenga BA, Musunzaji PS, Mbakaya JO, Aswani P, Irungu LW, Mukoko D, Vulule J, Kitron U, LaBeaud AD: **Characterization and productivity profiles of Aedes aegypti (L.) breeding habitats across rural and urban landscapes in western and coastal Kenya.** *Parasit Vectors* 2017, **10:**331.

71.	Ndenga BA, Mutuku FM, Ngugi HN, Mbakaya JO, Aswani P, Musunzaji PS, Vulule J, Mukoko D, Kitron U, LaBeaud AD: **Characteristics of Aedes aegypti adult mosquitoes in rural and urban areas of western and coastal Kenya.** *PLoS One* 2017, **12:**e0189971.

72.	Agha SB, Tchouassi DP, Bastos ADS, Sang R: **Dengue and yellow fever virus vectors: seasonal abundance, diversity and resting preferences in three Kenyan cities.** *Parasit Vectors* 2017, **10:**628.

73.	Kajeguka D, Kaaya R, Desrochers R, Iranpour M, Kavishe R, Mwakalinga S, Schioler K, Alifrangis M, Lindsay L, Dibernardo A, et al: **Mapping clusters of chikungunya and dengue transmission in northern Tanzania using disease exposure and vector data.** *Tanzania Journal of Health Research* 2017, **19 (4)**.

74.	Mohamed A, Ali A, Harbach R, Reeves R, Ibrahim K, AhmedMohmed M, Azrag R: **Aedes mosquitoes in the Republic of the Sudan, with dichotomous keys for the adult and larval stages.** *Journal of Natural History* 2017.

75.	Zahouli JBZ, Koudou BG, Müller P, Malone D, Tano Y, Utzinger J: **Effect of land-use changes on the abundance, distribution, and host-seeking behavior of Aedes arbovirus vectors in oil palm-dominated landscapes, southeastern Côte d'Ivoire.** *PLoS One* 2017, **12:**e0189082.

76.	Guindo-Coulibaly N, Diakite NR, Adja AM, Coulibaly JT, Bassa KF, Konan YL, N'Goran KE: **Biology of two larval morphological phenotypes of Aedes aegypti in Abidjan, Côte d'Ivoire.** *Bull Entomol Res* 2018, **108:**540-546.

77.	Chepkorir E, Venter M, Lutomiah J, Mulwa F, Arum S, Tchouassi DP, Sang R: **The occurrence, diversity and blood feeding patterns of potential vectors of dengue and yellow fever in Kacheliba, West Pokot County, Kenya.** *Acta Trop* 2018, **186:**50-57.

78.	Iwashita H, Higa Y, Futami K, Lutiali PA, Njenga SM, Nabeshima T, Minakawa N: **Mosquito arbovirus survey in selected areas of Kenya: detection of insect-specific virus.** *Trop Med Health* 2018, **46:**19.

79.	Kone B, Konan L, Fofana D, Koffi A, Coulibaly D, Benie J: **Entomological evaluation of the risk of spread ofthe dengue 3 epidemic in the health district ofCocody (Abidjan, Côte d’Ivoire).** *International Journal of Mosquito Research* 2018, **5 (5):**19-24.

80.	Mugabe VA, Ali S, Chelene I, Monteiro VO, Guiliche O, Muianga AF, Mula F, António V, Chongo I, Oludele J, et al: **Evidence for chikungunya and dengue transmission in Quelimane, Mozambique: Results from an investigation of a potential outbreak of chikungunya virus.** *PLoS One* 2018, **13:**e0192110.

81.	Ndiaye EH, Diallo D, Fall G, Ba Y, Faye O, Dia I, Diallo M: **Arboviruses isolated from the Barkedji mosquito-based surveillance system, 2012-2013.** *BMC Infect Dis* 2018, **18:**642.

82.	Mutebi JP, Crabtree MB, Kading RC, Powers AM, Ledermann JP, Mossel EC, Zeidner N, Lutwama JJ, Miller BR: **Mosquitoes of Northwestern Uganda.** *J Med Entomol* 2018, **55:**587-599.

83.	Delatte H, Desvars A, Bouetard A, Bord S, Gimonneau G, Vourc'h G, Fontenille D: **Blood-feeding behavior of Aedes albopictus, a vector of Chikungunya on La Reunion.** *Vector-Borne and Zoonotic Diseases* 2018.

84.	Aikpon R, Dramane G, Klotoé J, Brettenny M, Lawani Y, Aikpon G, Yadouléton A: **Assessment of population dynamics and biting trends of *Aedes aegypti* in northern Benin: public health implications.** *International Journal of Mosquito Research* 2019, **6**.

85.	Sissoko F, Junnila A, Traore MM, Traore SF, Doumbia S, Dembele SM, Schlein Y, Traore AS, Gergely P, Xue RD, et al: **Frequent sugar feeding behavior by Aedes aegypti in Bamako, Mali makes them ideal candidates for control with attractive toxic sugar baits (ATSB).** *PLoS One* 2019, **14:**e0214170.

86.	Fofana D, Beugré JMV, Yao-Acapovi GL, Lendzele SS: **Risk of Dengue Transmission in Cocody (Abidjan, Ivory Coast).** *J Parasitol Res* 2019, **2019:**4914137.

87.	Gneme A, Kabore J, Kpoda N, Gnoumou P, Mano K, Kabre G: **Diversity and abundance of mosquitoes in the city of Ouagadougou, Burkina Faso.** *International Journal of Entomology Research* 2019.

88.	Gorsich EE, Beechler BR, van Bodegom PM, Govender D, Guarido MM, Venter M, Schrama M: **A comparative assessment of adult mosquito trapping methods to estimate spatial patterns of abundance and community composition in southern Africa.** *Parasit Vectors* 2019, **12:**462.

89.	Mulchandani R, Massebo F, Bocho F, Jeffries CL, Walker T, Messenger LA: **A community-level investigation following a yellow fever virus outbreak in South Omo Zone, South-West Ethiopia.** *PeerJ* 2019, **7:**e6466.

90.	Iyaloo D, Doumma A, Mahamadou I, Arzika I, Soumana A, Kadri S, Idi I, Testa J: **Seasonal changes in the distribution and relative abundance of the dengue vector, Aedes albopictus in two villages in Mauritius: implications for a sterile release programme.** *African Entomology* 2019, **27**.

91.	Labbo R, Doumma A, Mahamadou I, Arzika I, Soumana A, Kadri S, Idi I, Testa J: **Distribution and relative densities of Aedes aegypti in Niger.** *Med Sante Trop* 2019, **29:**47-54.

92.	Karuitha M, Bargul J, Lutomiah J, Muriu S, Nzovu J, Sang R, Mwangangi J, Mbogo C: **Larval habitat diversity and mosquito species distribution along the coast of Kenya.** *Wellcome Open Res* 2019, **4:**175.

93.	Nebinur M, Bashir N, Dukeen M: **Presence, characterization and productivity profiles of Aedes mosquitoes (Diptera: Culicidae), in Barentu, Gash Barka Zone, Eritrea.** *International Journal of Mosquito Research* 2019.

94.	Thabet HS, Fawaz EY, Badziklou K, Tag ElDin RA, Kaldas RM, Fahmy NT, Tamekloe TA, Kere-Banla A, Diclaro JW: **Preliminary Screening of Mosquito Spatial Distribution in Togo: With Special Focus on the Aedes (Diptera: Culicidae) Species.** *J Med Entomol* 2019, **56:**1154-1158.

95.	Agha SB, Tchouassi DP, Turell MJ, Bastos ADS, Sang R: **Entomological assessment of dengue virus transmission risk in three urban areas of Kenya.** *PLoS Negl Trop Dis* 2019, **13:**e0007686.

96.	Agwu E, Isaac C, Igbinosa I: **Detection of yellow fever and dengue viruses in mosquitoes between 2014 and 2015 in Bayelsa and Benue states of Nigeria.** *Acta Entomologica Serbica* 2019, **24**.

97.	Nwachukwu WE, Yusuff H, Nwangwu U, Okon A, Ogunniyi A, Imuetinyan-Clement J, Besong M, Ayo-Ajayi P, Nikau J, Baba A, et al: **The response to re-emergence of yellow fever in Nigeria, 2017.** *Int J Infect Dis* 2020, **92:**189-196.

98.	Philbert A, Msonga A: **Dengue vector distribution and their infection status in selected regions in Tanzania.** *Tanzania Journal of Science* 2020  **46**.

99.	Diallo D, Diallo M: **Resting behavior of Aedes aegypti in southeastern Senegal.** *Parasit Vectors* 2020, **13:**356.

100.	Namountougou M, Soma D, Kabore D, N'do S, Kientega M, Sawadogo J, Kagone T, Kania D, Sanou R, Sangare I, et al: **Characterisation of the breeding sites and insecticide resistance of Aedes aegypti population in the city of Bobo-Dioulaso, Burkina Faso.** *African Entomology* 2020, **28**.

101.	Eastwood G, Sang RC, Lutomiah J, Tunge P, Weaver SC: **Sylvatic Mosquito Diversity in Kenya-Considering Enzootic Ecology of Arboviruses in an Era of Deforestation.** *Insects* 2020, **11**.

102.	Johnson T, Braack L, Guarido M, Venter M, Gouveia Almeida AP: **Mosquito community composition and abundance at contrasting sites in northern South Africa, 2014-2017.** *J Vector Ecol* 2020, **45:**104-117.

103.	Irikannu K, Onyido A, Nwankwo E, Umeanaeto P, Onwube O, Ogaraku J, Ezeagwuna D, Onyebueke A, Okoduwa A: **A survey of man-biting mosquito species in a tropical rainforest community in the Southeastern Nigeria.** *Environment and Ecology* 2020, **38**.

104.	Abílio AP, Kampango A, Armando EJ, Gudo ES, das Neves LCB, Parreira R, Sidat M, Fafetine JM, de Almeida APG: **First confirmed occurrence of the yellow fever virus and dengue virus vector Aedes (Stegomyia) luteocephalus (Newstead, 1907) in Mozambique.** *Parasit Vectors* 2020, **13:**350.

105.	Diallo D, Fall G, Diagne CT, Gaye A, Ba Y, Dia I, Faye O, Diallo M: **Concurrent amplification of Zika, chikungunya, and yellow fever virus in a sylvatic focus of arboviruses in Southeastern Senegal, 2015.** *BMC Microbiol* 2020, **20:**181.

106.	Rose NH, Sylla M, Badolo A, Lutomiah J, Ayala D, Aribodor OB, Ibe N, Akorli J, Otoo S, Mutebi JP, et al: **Climate and Urbanization Drive Mosquito Preference for Humans.** *Curr Biol* 2020, **30:**3570-3579.e3576.

107.	Captain-Esoah M, Kweku Baidoo P, Frempong KK, Adabie-Gomez D, Chabi J, Obuobi D, Kwame Amlalo G, Balungnaa Veriegh F, Donkor M, Asoala V, et al: **Biting Behavior and Molecular Identification of Aedes aegypti (Diptera: Culicidae) Subspecies in Some Selected Recent Yellow Fever Outbreak Communities in Northern Ghana.** *J Med Entomol* 2020, **57:**1239-1245.

108.	Mayi MPA, Bamou R, Djiappi-Tchamen B, Fontaine A, Jeffries CL, Walker T, Antonio-Nkondjio C, Cornel AJ, Tchuinkam T: **Habitat and Seasonality Affect Mosquito Community Composition in the West Region of Cameroon.** *Insects* 2020, **11**.

109.	Fagbohun I, Idowu E, Awolola T, Otubanjo O: **Seasonal abundance and larval habitats characterization of mosquito species in Lagos State, Nigeria.** *Scientific African* 2020, **10**.

110.	Musa AA, Muturi MW, Musyoki AM, Ouso DO, Oundo JW, Makhulu EE, Wambua L, Villinger J, Jeneby MM: **Arboviruses and Blood Meal Sources in Zoophilic Mosquitoes at Human-Wildlife Interfaces in Kenya.** *Vector Borne Zoonotic Dis* 2020, **20:**444-453.

111.	Kampango A, Furu P, Sarath DL, Haji KA, Konradsen F, Schiøler KL, Alifrangis M, Weldon CW, Saleh F: **Targeted elimination of species-rich larval habitats can rapidly collapse arbovirus vector mosquito populations at hotel compounds in Zanzibar.** *Med Vet Entomol* 2021, **35:**523-533.

112.	Waldetensai A, Gemechu F, Kinfe E, Amare H, Hagos S, Teshome D, Hailemariam A, Nigatu W, Tadesse A, Keneni D, et al: **Aedes mosquito responses to control interventionsagainst the Chikungunya outbreak of Dire Dawa, Eastern Ethiopia.** *International Journal of Tropical Insect Science* 2021.

113.	Onwuzulike I, Onyebueke A, Irikannu K, Nzeukwu C, Ogbonna C, Nwangwu R, Ochiaka C: **Relative abundance and diversity of man-biting mosquito species before and after indoor residual spraying programme in Awka and Environs, Anambra State, Nigeria.** *Trends in Entomology* 2021, **17**.

114.	Diouf B, Sene NM, Ndiaye EH, Gaye A, Ngom EHM, Gueye A, Seck F, Diagne CT, Dia I, Diallo M, Diallo D: **Resting Behavior of Blood-Fed Females and Host Feeding Preferences of Aedes aegypti (Diptera: Culicidae) Morphological Forms in Senegal.** *J Med Entomol* 2021, **58:**2467-2473.

115.	Diouf G, Seck MT, Ciss M, Faye B, Biteye B, Bakhoum MT, Fall AG: **Improving the efficiency of the BG sentinel 2 trap to assess the activity of Aedes (Stegomyia) aegypti [Linnaeus, 1762] in Senegal.** *Acta Trop* 2021, **222:**106065.

116.	Gutu MA, Bekele A, Seid Y, Mohammed Y, Gemechu F, Woyessa AB, Tayachew A, Dugasa Y, Gizachew L, Idosa M, et al: **Another dengue fever outbreak in Eastern Ethiopia-An emerging public health threat.** *PLoS Negl Trop Dis* 2021, **15:**e0008992.

117.	Kpan M, Adja A, Guindo-Coulibaly N, Assouho K, Kouadio A: **Spatial Heterogeneity and Seasonal Distribution of Aedes (Stegomyia) aegypti (L) in Abidjan, Cote d'Ivoire.** *Vector Borne Zoonotic Diseases* 2021.

118.	Hamaidia K, Soltani N: **New report of Aedes albopictus in Souk Ahras, Northeast Algeria.** *Biodiversitas* 2021.

119.	Chiuya T, Masiga DK, Falzon LC, Bastos ADS, Fèvre EM, Villinger J: **A survey of mosquito-borne and insect-specific viruses in hospitals and livestock markets in western Kenya.** *PLoS One* 2021, **16:**e0252369.

120.	Guarido MM, Riddin MA, Johnson T, Braack LEO, Schrama M, Gorsich EE, Brooke BD, Almeida APG, Venter M: **Aedes species (Diptera: Culicidae) ecological and host feeding patterns in the north-eastern parts of South Africa, 2014-2018.** *Parasit Vectors* 2021, **14:**339.

121.	Irikannu K, Onyido A, Umeanaeto P, Onyebueke A, Nzeukwu C, Ogbonna C, Ezeagwuna D, Ogaraku J, Asogwa K: **Breeding ecology and physicochemical properties of mosquito breeding sites in Awka South Local Government Area, Anambra State, Nigeria.** *Trends in Entomology* 2021.

122.	Karisa J, Muriu S, Omuoyo D, Karia B, Ngari M, Nyamwaya D, Rono M, Warimwe G, Mwangangi J, Mbogo CM: **Urban Ecology of Arboviral Mosquito Vectors Along the Kenyan Coast.** *J Med Entomol* 2021, **58:**428-438.

123.	Ngingo BL, Mboera LEG, Chengula A, Machelle I, Makange MR, Msolla M, Mwanyika GO, Rugarabamu S, Misinzo G: **Aedes aegypti abundance, larval indices and risk for dengue virus transmission in Kinondoni district, Tanzania.** *Trop Med Health* 2022, **50:**1.

124.	Owusu-Asenso CM, Mingle JAA, Weetman D, Afrane YA: **Spatiotemporal distribution and insecticide resistance status of Aedes aegypti in Ghana.** *Parasit Vectors* 2022, **15:**61.

125.	Kouadio A, Guindo-Coulibaly N, Adja A, Kpan S, Assouho K, Zoh D, Yapi A, Azongnibo K, Sadia-Kacou A: **High density in dry season and resistance to pyrethroids of Aedes aegypti populations in Bouake, Cote d'Ivoire.** *International Journal of Tropical Insect Science* 2022.

126.	Djiappi-Tchamen B, Nana-Ndjangwo MS, Nchoutpouen E, Makoudjou I, Ngangue-Siewe IN, Talipouo A, Mayi MPA, Awono-Ambene P, Wondji C, Tchuinkam T, Antonio-Nkondjio C: **Mosquito Surveillance Using Ovitraps, Sweep Nets, and Biogent Traps in the City of Yaoundé, Cameroon.** *Insects* 2022, **13**.

127.	Badolo A, Sombié A, Yaméogo F, Wangrawa DW, Sanon A, Pignatelli PM, Viana M, Kanuka H, Weetman D, McCall PJ: **First comprehensive analysis of Aedes aegypti bionomics during an arbovirus outbreak in west Africa: Dengue in Ouagadougou, Burkina Faso, 2016-2017.** *PLoS Negl Trop Dis* 2022, **16:**e0010059.

128.	Sene NM, Diouf B, Gaye A, Ndiaye EH, Ngom EM, Gueye A, Seck F, Diagne CT, Dia I, Diallo D, Diallo M: **Blood Feeding Patterns of Aedes aegypti Populations in Senegal.** *Am J Trop Med Hyg* 2022, **106:**1402-1405.

129.	Stoek F, Barry Y, Ba A, Schulz A, Rissmann M, Wylezich C, Sadeghi B, Beyit AD, Eisenbarth A, N'diaye FB, et al: **Mosquito survey in Mauritania: Detection of Rift Valley fever virus and dengue virus and the determination of feeding patterns.** *PLoS Negl Trop Dis* 2022, **16:**e0010203.

130.	Kamau WW, Sang R, Ogola EO, Rotich G, Getugi C, Agha SB, Menza N, Torto B, Tchouassi DP: **Survival rate, blood feeding habits and sibling species composition of Aedes simpsoni complex: Implications for arbovirus transmission risk in East Africa.** *PLoS Negl Trop Dis* 2022, **16:**e0010171.

131.	Ndenga BA, Mutuku FM, Ngugi HN, Mbakaya JO, Mukoko D, Kitron U, LaBeaud AD: **Night Time Extension of Aedes aegypti Human Blood Seeking Activity.** *Am J Trop Med Hyg* 2022, **107:**208-210.
