# Supplementary material for: Building resilience against the growing threat of arboviruses II: a scoping review of Aedes vector competence, larval and adult bionomics and species distribution in Africa
Source: Parasit Vectors. 2026 Jul 28;19:460. doi: 10.1186/s13071-026-07552-5 (PMC13693134; doi:10.1186/s13071-026-07552-5)
Supplement: Supplementary file 4 [file 13071_2026_7552_MOESM4_ESM.docx]

| **Supplementary Table 2.2 Use of CO_2_ with adult traps** | | | | | | |
| --- | --- | --- | --- | --- | --- | --- |
| **No.** | **Study** | **Publication year** | **Country** | **CO^2^ source** | **Traps baited with CO^2^** | **Ref** |
| 1 | Linthicum et al. | 1985 | Kenya | Dry ice (2kg/trap) | CDC-LT | [1] |
| 2 | Tchouassi et al. | 2012 | Kenya | Dry ice (1kg p/trap) | CDC-LT | [2] |
| 3 | Mutebi et al. | 2012 | Uganda | Dry ice (0.5kg/trap) | CDC-LT | [3] |
| 4 | Crabtree et al. | 2013 | Uganda | Dry ice | CDC-LT | [4] |
| 5 | Kaddumukasa et al. | 2015 | Uganda | Dry ice | CDC-LT | [5] |
| 6 | Omondi et al. | 2015 | Kenya | Not described | CDC-LT | [6] |
| 7 | Mweya et al. | 2015 | Tanzania | Not described and propane tank | CDC-LT and Mosquito Magnet Liberty Plus | [7] |
| 8 | Lutomiah et al. | 2016 | Kenya | Dry ice (0.5kg/trap) | CDC-LT | [8] |
| 9 | Mboera et al. | 2016 | Tanzania | Propane tank | Mosquito Magnet Liberty Plus | [9] |
| 10 | Agha et al. | 2017 | Kenya | Dry ice (0.5kg p/trap) | BG-Sentinel | [10] |
| 11 | Kajeguka et al. | 2017 | Tanzania | Propane tank | Mosquito Magnet Liberty Plus | [11] |
| 12 | Ndenga et al. | 2017 | Kenya | Yeast and sugar | BG-Sentinel | [12] |
| 13 | Chepkorir et al. | 2018 | Kenya | Dry ice (2L thermos) | BG-Sentinel | [13] |
| 14 | Ndiaye et al. | 2018 | Senegal | Not described | CDC-LT | [14] |
| 15 | Mutebi et al. | 2018 | Uganda | Dry ice (0.5kg/trap) | CDC-LT | [15] |
| 16 | Karuitha et al. | 2019 | Kenya | Not described | CDC-LT | [16] |
| 17 | Agha et al. | 2019 | Kenya | Dry ice | BG-Sentinel | [17] |
| 18 | Eastwood et al. | 2020 | Kenya | Dry ice | CDC-LT | [18] |
| 19 | Johnson et al. | 2020 | South Africa | Dry ice | CDC-LT and mosquito tent trap | [19] |
| 20 | Philbert et al. | 2020 | Tanzania | Propane tank | Mosquito Magnet Liberty Plus | [20] |
| 21 | Musa et al. | 2020 | Kenya | Dry ice | CDC-LT and BG-Sentinel | [21] |
| 22 | Chiuya et al. | 2021 | Kenya | Dry ice | CDC-LT and BG-Sentinel | [22] |
| 23 | Guarido et al. | 2021 | South Africa | Not described | CDC-LT, BG-Sentinel and mosquito net trap | [23] |
| 24 | Karisa et al. | 2021 | Kenya | Dry ice (3kg/trap) | BG-Sentinel | [24] |
| 25 | Ngingo et al. | 2022 | Tanzania | Propane tank | Mosquito Magnet Liberty Plus | [25] |
| 26 | Owusu-Asenso et al. | 2022 | Ghana | Yeast and sugar | BG-Sentinel | [26] |
| 27 | Djiappi-Tchamen et al. | 2022 | Cameroon | Yeast and sugar | BG-Sentinel | [27] |
| 28 | Stoek et al. | 2022 | Mauritania | CO_2_-filled bottles | Heavy-duty EVS | [28] |
| 29 | Kamau et al. | 2022 | Kenya | Not described | BG-Sentinel | [29] |

**References**

1.	Linthicum KJ, Kaburia HF, Davies FG, Lindqvist KJ: **A blood meal analysis of engorged mosquitoes found in Rift Valley fever epizootics area in Kenya.** *J Am Mosq Control Assoc* 1985, **1:**93-95.

2.	Tchouassi DP, Sang R, Sole CL, Bastos AD, Mithoefer K, Torto B: **Sheep skin odor improves trap captures of mosquito vectors of Rift Valley fever.** *PLoS Negl Trop Dis* 2012, **6:**e1879.

3.	Mutebi JP, Crabtree MB, Kading RC, Powers AM, Lutwama JJ, Miller BR: **Mosquitoes of western Uganda.** *J Med Entomol* 2012, **49:**1289-1306.

4.	Crabtree MB, Kading RC, Mutebi JP, Lutwama JJ, Miller BR: **Identification of host blood from engorged mosquitoes collected in western Uganda using cytochrome oxidase I gene sequences.** *J Wildl Dis* 2013, **49:**611-626.

5.	Kaddumukasa M, Kayondo J, Masiga D, Akoi A, Lutwama J, Masembe C: **High proportion of mosquito vectors in Zika forest, Uganda, feeding on humans has implications for the spread of new arbovirus pathogens.** *African Journal of Biotechnology* 2015, **14**.

6.	Omondi D, Masiga DK, Ajamma YU, Fielding BC, Njoroge L, Villinger J: **Unraveling Host-Vector-Arbovirus Interactions by Two-Gene High Resolution Melting Mosquito Bloodmeal Analysis in a Kenyan Wildlife-Livestock Interface.** *PLoS One* 2015, **10:**e0134375.

7.	Mweya CN, Kimera SI, Mellau LS, Mboera LE: **Inter-epidemic abundance and distribution of potential mosquito vectors for Rift Valley fever virus in Ngorongoro district, Tanzania.** *Glob Health Action* 2015, **8:**25929.

8.	Lutomiah J, Barrera R, Makio A, Mutisya J, Koka H, Owaka S, Koskei E, Nyunja A, Eyase F, Coldren R, Sang R: **Dengue Outbreak in Mombasa City, Kenya, 2013-2014: Entomologic Investigations.** *PLoS Negl Trop Dis* 2016, **10:**e0004981.

9.	Mboera LE, Mweya CN, Rumisha SF, Tungu PK, Stanley G, Makange MR, Misinzo G, De Nardo P, Vairo F, Oriyo NM: **The Risk of Dengue Virus Transmission in Dar es Salaam, Tanzania during an Epidemic Period of 2014.** *PLoS Negl Trop Dis* 2016, **10:**e0004313.

10.	Agha SB, Tchouassi DP, Bastos ADS, Sang R: **Dengue and yellow fever virus vectors: seasonal abundance, diversity and resting preferences in three Kenyan cities.** *Parasit Vectors* 2017, **10:**628.

11.	Kajeguka D, Kaaya R, Desrochers R, Iranpour M, Kavishe R, Mwakalinga S, Schioler K, Alifrangis M, Lindsay L, Dibernardo A, et al: **Mapping clusters of chikungunya and dengue transmission in northern Tanzania using disease exposure and vector data.** *Tanzania Journal of Health Research* 2017, **19 (4)**.

12.	Ndenga BA, Mutuku FM, Ngugi HN, Mbakaya JO, Aswani P, Musunzaji PS, Vulule J, Mukoko D, Kitron U, LaBeaud AD: **Characteristics of Aedes aegypti adult mosquitoes in rural and urban areas of western and coastal Kenya.** *PLoS One* 2017, **12:**e0189971.

13.	Chepkorir E, Venter M, Lutomiah J, Mulwa F, Arum S, Tchouassi DP, Sang R: **The occurrence, diversity and blood feeding patterns of potential vectors of dengue and yellow fever in Kacheliba, West Pokot County, Kenya.** *Acta Trop* 2018, **186:**50-57.

14.	Ndiaye EH, Diallo D, Fall G, Ba Y, Faye O, Dia I, Diallo M: **Arboviruses isolated from the Barkedji mosquito-based surveillance system, 2012-2013.** *BMC Infect Dis* 2018, **18:**642.

15.	Mutebi JP, Crabtree MB, Kading RC, Powers AM, Ledermann JP, Mossel EC, Zeidner N, Lutwama JJ, Miller BR: **Mosquitoes of Northwestern Uganda.** *J Med Entomol* 2018, **55:**587-599.

16.	Karuitha M, Bargul J, Lutomiah J, Muriu S, Nzovu J, Sang R, Mwangangi J, Mbogo C: **Larval habitat diversity and mosquito species distribution along the coast of Kenya.** *Wellcome Open Res* 2019, **4:**175.

17.	Agha SB, Tchouassi DP, Turell MJ, Bastos ADS, Sang R: **Entomological assessment of dengue virus transmission risk in three urban areas of Kenya.** *PLoS Negl Trop Dis* 2019, **13:**e0007686.

18.	Eastwood G, Sang RC, Lutomiah J, Tunge P, Weaver SC: **Sylvatic Mosquito Diversity in Kenya-Considering Enzootic Ecology of Arboviruses in an Era of Deforestation.** *Insects* 2020, **11**.

19.	Johnson T, Braack L, Guarido M, Venter M, Gouveia Almeida AP: **Mosquito community composition and abundance at contrasting sites in northern South Africa, 2014-2017.** *J Vector Ecol* 2020, **45:**104-117.

20.	Philbert A, Msonga A: **Dengue vector distribution and their infection status in selected regions in Tanzania.** *Tanzania Journal of Science* 2020  **46**.

21.	Musa AA, Muturi MW, Musyoki AM, Ouso DO, Oundo JW, Makhulu EE, Wambua L, Villinger J, Jeneby MM: **Arboviruses and Blood Meal Sources in Zoophilic Mosquitoes at Human-Wildlife Interfaces in Kenya.** *Vector Borne Zoonotic Dis* 2020, **20:**444-453.

22.	Chiuya T, Masiga DK, Falzon LC, Bastos ADS, Fèvre EM, Villinger J: **A survey of mosquito-borne and insect-specific viruses in hospitals and livestock markets in western Kenya.** *PLoS One* 2021, **16:**e0252369.

23.	Guarido MM, Riddin MA, Johnson T, Braack LEO, Schrama M, Gorsich EE, Brooke BD, Almeida APG, Venter M: **Aedes species (Diptera: Culicidae) ecological and host feeding patterns in the north-eastern parts of South Africa, 2014-2018.** *Parasit Vectors* 2021, **14:**339.

24.	Karisa J, Muriu S, Omuoyo D, Karia B, Ngari M, Nyamwaya D, Rono M, Warimwe G, Mwangangi J, Mbogo CM: **Urban Ecology of Arboviral Mosquito Vectors Along the Kenyan Coast.** *J Med Entomol* 2021, **58:**428-438.

25.	Ngingo BL, Mboera LEG, Chengula A, Machelle I, Makange MR, Msolla M, Mwanyika GO, Rugarabamu S, Misinzo G: **Aedes aegypti abundance, larval indices and risk for dengue virus transmission in Kinondoni district, Tanzania.** *Trop Med Health* 2022, **50:**1.

26.	Owusu-Asenso CM, Mingle JAA, Weetman D, Afrane YA: **Spatiotemporal distribution and insecticide resistance status of Aedes aegypti in Ghana.** *Parasit Vectors* 2022, **15:**61.

27.	Djiappi-Tchamen B, Nana-Ndjangwo MS, Nchoutpouen E, Makoudjou I, Ngangue-Siewe IN, Talipouo A, Mayi MPA, Awono-Ambene P, Wondji C, Tchuinkam T, Antonio-Nkondjio C: **Mosquito Surveillance Using Ovitraps, Sweep Nets, and Biogent Traps in the City of Yaoundé, Cameroon.** *Insects* 2022, **13**.

28.	Stoek F, Barry Y, Ba A, Schulz A, Rissmann M, Wylezich C, Sadeghi B, Beyit AD, Eisenbarth A, N'diaye FB, et al: **Mosquito survey in Mauritania: Detection of Rift Valley fever virus and dengue virus and the determination of feeding patterns.** *PLoS Negl Trop Dis* 2022, **16:**e0010203.

29.	Kamau WW, Sang R, Ogola EO, Rotich G, Getugi C, Agha SB, Menza N, Torto B, Tchouassi DP: **Survival rate, blood feeding habits and sibling species composition of Aedes simpsoni complex: Implications for arbovirus transmission risk in East Africa.** *PLoS Negl Trop Dis* 2022, **16:**e0010171.
