# Supplementary material for: Building resilience against the growing threat of arboviruses II: a scoping review of Aedes vector competence, larval and adult bionomics and species distribution in Africa
Source: Parasit Vectors. 2026 Jul 28;19:460. doi: 10.1186/s13071-026-07552-5 (PMC13693134; doi:10.1186/s13071-026-07552-5)
Supplement: Supplementary file 5 [file 13071_2026_7552_MOESM5_ESM.docx]

| **Supplementary Table 2.3 Bionomics- *Aedes* Biting Times** | | | | | |
| --- | --- | --- | --- | --- | --- |
| Number | Study | Publication Year | Country | Mosquito Species | Ref |
| 1 | Nwoke et al. | 1993 | Nigeria | *Ae. aegypti, Ae. vittatus* | [1] |
| 2 | Diarrassouba et al. | 1997 | Côte d’Ivoire | *Ae. aegypti, Ae. fowleri* | [2] |
| 3 | Delatte et al. | 2010 | La Réunion | *Ae. albopictus* | [3] |
| 4 | Kamgang et al. | 2012 | Cameroon | *Ae. albopictus* | [4] |
| 5 | Ndenga et al. | 2017 | Kenya | *Ae. aegypti* | [5] |
| 6 | Aikpon et al. | 2019 | Benin | *Ae. aegypti* | [6] |
| 7 | Sissoko et al. | 2019 | Mali | *Ae. aegypti* | [7] |
| 8 | Labbo et al. | 2019 | Niger | *Ae. aegypti, Ae. vittatus, Ae. luteocephalus* | [8] |
| 9 | Agwu et al. | 2019 | Nigeria | *Ae. luteocephalus, Ae, aegypti* | [9] |
| 10 | Irikannu et al. | 2020 | Nigeria | *Ae. aegypti, Ae. albopictus* | [10] |
| 11 | Captain-Esoah et al. | 2020 | Ghana | *Ae. aegypti, Ae. vittatus* | [11] |
| 12 | Diouf et al. | 2021 | Senegal | *Ae. aegypti* | [12] |
| 13 | Ndenga et al. | 2022 | Kenya | *Ae. aegypti* | [13] |

**References**

1.	Nwoke BEB, Nduka FO, Okereke OM, Ehighibe OC: **Sustainable urban development and human health: septic tank as a major breeding habitat of mosquito vectors of human diseases in south-eastern Nigeria.** *Applied Parasitology* 1993, **34:**1-10.

2.	Diarrassouba S, Dossou-Yovo J: **Rythme d'activité atypique chez *Aedes aegypti* en zone de savane sub-soudanienne de Côte d'Ivoire.** *Bulletin de la Societe de pathologie exotique* 1997, **90**.

3.	Delatte H, Desvars A, Bouétard A, Bord S, Gimonneau G, Vourc'h G, Fontenille D: **Blood-feeding behavior of Aedes albopictus, a vector of Chikungunya on La Réunion.** *Vector Borne Zoonotic Dis* 2010, **10:**249-258.

4.	Kamgang B, Nchoutpouen E, Simard F, Paupy C: **Notes on the blood-feeding behavior of Aedes albopictus (Diptera: Culicidae) in Cameroon.** *Parasit Vectors* 2012, **5:**57.

5.	Ndenga BA, Mutuku FM, Ngugi HN, Mbakaya JO, Aswani P, Musunzaji PS, Vulule J, Mukoko D, Kitron U, LaBeaud AD: **Characteristics of Aedes aegypti adult mosquitoes in rural and urban areas of western and coastal Kenya.** *PLoS One* 2017, **12:**e0189971.

6.	Aikpon R, Dramane G, Klotoé J, Brettenny M, Lawani Y, Aikpon G, Yadouléton A: **Assessment of population dynamics and biting trends of *Aedes aegypti* in northern Benin: public health implications.** *International Journal of Mosquito Research* 2019, **6**.

7.	Sissoko F, Junnila A, Traore MM, Traore SF, Doumbia S, Dembele SM, Schlein Y, Traore AS, Gergely P, Xue RD, et al: **Frequent sugar feeding behavior by Aedes aegypti in Bamako, Mali makes them ideal candidates for control with attractive toxic sugar baits (ATSB).** *PLoS One* 2019, **14:**e0214170.

8.	Labbo R, Doumma A, Mahamadou I, Arzika I, Soumana A, Kadri S, Idi I, Testa J: **Distribution and relative densities of Aedes aegypti in Niger.** *Med Sante Trop* 2019, **29:**47-54.

9.	Agwu E, Isaac C, Igbinosa I: **Detection of yellow fever and dengue viruses in mosquitoes between 2014 and 2015 in Bayelsa and Benue states of Nigeria.** *Acta Entomologica Serbica* 2019, **24**.

10.	Irikannu K, Onyido A, Nwankwo E, Umeanaeto P, Onwube O, Ogaraku J, Ezeagwuna D, Onyebueke A, Okoduwa A: **A survey of man-biting mosquito species in a tropical rainforest community in the Southeastern Nigeria.** *Environment and Ecology* 2020, **38**.

11.	Captain-Esoah M, Kweku Baidoo P, Frempong KK, Adabie-Gomez D, Chabi J, Obuobi D, Kwame Amlalo G, Balungnaa Veriegh F, Donkor M, Asoala V, et al: **Biting Behavior and Molecular Identification of Aedes aegypti (Diptera: Culicidae) Subspecies in Some Selected Recent Yellow Fever Outbreak Communities in Northern Ghana.** *J Med Entomol* 2020, **57:**1239-1245.

12.	Diouf G, Seck MT, Ciss M, Faye B, Biteye B, Bakhoum MT, Fall AG: **Improving the efficiency of the BG sentinel 2 trap to assess the activity of Aedes (Stegomyia) aegypti [Linnaeus, 1762] in Senegal.** *Acta Trop* 2021, **222:**106065.

13.	Ndenga BA, Mutuku FM, Ngugi HN, Mbakaya JO, Mukoko D, Kitron U, LaBeaud AD: **Night Time Extension of Aedes aegypti Human Blood Seeking Activity.** *Am J Trop Med Hyg* 2022, **107:**208-210.
