# Supplementary material for: Building resilience against the growing threat of arboviruses II: a scoping review of Aedes vector competence, larval and adult bionomics and species distribution in Africa
Source: Parasit Vectors. 2026 Jul 28;19:460. doi: 10.1186/s13071-026-07552-5 (PMC13693134; doi:10.1186/s13071-026-07552-5)
Supplement: Supplementary file 6 [file 13071_2026_7552_MOESM6_ESM.docx]

| **Supplementary Table 2.4. Bionomics- *Aedes* Blood-meal Publications** | | | | | | | | |
| --- | --- | --- | --- | --- | --- | --- | --- | --- |
| **Number** | **Study** | **Publication Year** | **Country** | **Trap type** | **Mosquito species** | **% human-fed** | **Primary blood-meal source** | **Ref** |
| 1 | Crabtree et al. | 2013 | Kenya | CDC-LT with CO2 | - | - | Few blood-fed *Aedes* collected | [1] |
| 2 | Diallo et al. | 2013 | Senegal | Mechanical aspirator | Ae. taylori, Ae. vittatus | 0% (0/14, 0/16) | Non-human primates and avian hosts | [2] |
| 3 | Kaddumukasa et al. | 2015 | Uganda | CDC-LT with CO2 | Ae. africanus | 100% (2/2) | Human | [3] |
| 4 | Omondi et al. | 2015 | Kenya | CDC-LT and BG-Sentinel with CO2 | Ae. aegypti | 0% (0/1) | Dog (1/1) | [4] |
| 5 | Chepkorir et al. | 2018 | Kenya | BG-Sentinel with CO2 | Ae. aegypti | 4% (3/68) | Rock hyrax 79% (54/68) | [5] |
| 6 | Agha et al. | 2019 | Kenya | BG-Sentinel with CO2 and mechanical aspirator | Ae aegypti | 24% (24/102) | Several animal species (cow, goat, dog, cat) | [6] |
| 7 | Musa et al. | 2020 | Kenya | CDC-LT and BG-Sentinel with CO2 | Ae. aegypti | 90% (9/10) | Human | [7] |
| 8 | Diouf et al. | 2021 | Senegal | BG-Sentinel and mechanical aspirator | Ae. aegypti | 92.3% (1,056/1,138) (Aaa), 86.2% (319/370) (Aaf) | Human | [8] |
| 9 | Chiuya et al. | 2021 | Kenya | CDC-LT and BG-Sentinel with CO2 | Ae. aegypti | 50% (1/2) | Human/bovine | [9] |
| 10 | Guarido et al. | 2021 | South Africa | CDC-LT and BG-Sentinel with CO2 | Mixed Aedes | - | Several mammals | [10] |
| 11 | Karisa et al. | 2021 | Kenya | CDC-LT and BG-Sentinel | Ae. aegypti | 26.7% (4/15) | Unidentified (11/15) | [11] |
| 12 | Owusu-Asenso et al. | 2022 | Ghana | BG-Sentinel and mechanical aspirator | Ae. aegypti | 90% (18/20) | Human | [12] |
| 13 | Badolo et al. | 2022 | Burkina Faso | Mechanical aspirator | Ae. aegypti | 88% (158/180) | Human | [13] |
| 14 | Sene et al. | 2022 | Senegal | BG-Sentinel and mechanical aspirator | Ae. aegypti | 78.6% (964/1,226) | Human | [14] |
| 15 | Stoek et al. | 2022 | Mauritania | Heavy-duty EVS with CO2 | Ae. aegypti | 100% (2/2) | Human | [15] |

**References**

1.	Crabtree MB, Kading RC, Mutebi JP, Lutwama JJ, Miller BR: **Identification of host blood from engorged mosquitoes collected in western Uganda using cytochrome oxidase I gene sequences.** *J Wildl Dis* 2013, **49:**611-626.

2.	Diallo D, Chen R, Diagne CT, Ba Y, Dia I, Sall AA, Weaver SC, Diallo M: **Bloodfeeding patterns of sylvatic arbovirus vectors in southeastern Senegal.** *Trans R Soc Trop Med Hyg* 2013, **107:**200-203.

3.	Kaddumukasa M, Kayondo J, Masiga D, Akoi A, Lutwama J, Masembe C: **High proportion of mosquito vectors in Zika forest, Uganda, feeding on humans has implications for the spread of new arbovirus pathogens.** *African Journal of Biotechnology* 2015, **14**.

4.	Omondi D, Masiga DK, Ajamma YU, Fielding BC, Njoroge L, Villinger J: **Unraveling Host-Vector-Arbovirus Interactions by Two-Gene High Resolution Melting Mosquito Bloodmeal Analysis in a Kenyan Wildlife-Livestock Interface.** *PLoS One* 2015, **10:**e0134375.

5.	Chepkorir E, Venter M, Lutomiah J, Mulwa F, Arum S, Tchouassi DP, Sang R: **The occurrence, diversity and blood feeding patterns of potential vectors of dengue and yellow fever in Kacheliba, West Pokot County, Kenya.** *Acta Trop* 2018, **186:**50-57.

6.	Agha SB, Tchouassi DP, Turell MJ, Bastos ADS, Sang R: **Entomological assessment of dengue virus transmission risk in three urban areas of Kenya.** *PLoS Negl Trop Dis* 2019, **13:**e0007686.

7.	Musa AA, Muturi MW, Musyoki AM, Ouso DO, Oundo JW, Makhulu EE, Wambua L, Villinger J, Jeneby MM: **Arboviruses and Blood Meal Sources in Zoophilic Mosquitoes at Human-Wildlife Interfaces in Kenya.** *Vector Borne Zoonotic Dis* 2020, **20:**444-453.

8.	Diouf B, Sene NM, Ndiaye EH, Gaye A, Ngom EHM, Gueye A, Seck F, Diagne CT, Dia I, Diallo M, Diallo D: **Resting Behavior of Blood-Fed Females and Host Feeding Preferences of Aedes aegypti (Diptera: Culicidae) Morphological Forms in Senegal.** *J Med Entomol* 2021, **58:**2467-2473.

9.	Chiuya T, Masiga DK, Falzon LC, Bastos ADS, Fèvre EM, Villinger J: **A survey of mosquito-borne and insect-specific viruses in hospitals and livestock markets in western Kenya.** *PLoS One* 2021, **16:**e0252369.

10.	Guarido MM, Riddin MA, Johnson T, Braack LEO, Schrama M, Gorsich EE, Brooke BD, Almeida APG, Venter M: **Aedes species (Diptera: Culicidae) ecological and host feeding patterns in the north-eastern parts of South Africa, 2014-2018.** *Parasit Vectors* 2021, **14:**339.

11.	Karisa J, Muriu S, Omuoyo D, Karia B, Ngari M, Nyamwaya D, Rono M, Warimwe G, Mwangangi J, Mbogo CM: **Urban Ecology of Arboviral Mosquito Vectors Along the Kenyan Coast.** *J Med Entomol* 2021, **58:**428-438.

12.	Owusu-Asenso CM, Mingle JAA, Weetman D, Afrane YA: **Spatiotemporal distribution and insecticide resistance status of Aedes aegypti in Ghana.** *Parasit Vectors* 2022, **15:**61.

13.	Badolo A, Sombié A, Yaméogo F, Wangrawa DW, Sanon A, Pignatelli PM, Viana M, Kanuka H, Weetman D, McCall PJ: **First comprehensive analysis of Aedes aegypti bionomics during an arbovirus outbreak in west Africa: Dengue in Ouagadougou, Burkina Faso, 2016-2017.** *PLoS Negl Trop Dis* 2022, **16:**e0010059.

14.	Sene NM, Diouf B, Gaye A, Ndiaye EH, Ngom EM, Gueye A, Seck F, Diagne CT, Dia I, Diallo D, Diallo M: **Blood Feeding Patterns of Aedes aegypti Populations in Senegal.** *Am J Trop Med Hyg* 2022, **106:**1402-1405.

15.	Stoek F, Barry Y, Ba A, Schulz A, Rissmann M, Wylezich C, Sadeghi B, Beyit AD, Eisenbarth A, N'diaye FB, et al: **Mosquito survey in Mauritania: Detection of Rift Valley fever virus and dengue virus and the determination of feeding patterns.** *PLoS Negl Trop Dis* 2022, **16:**e0010203.
