# Supplementary material for: Building resilience against the growing threat of arboviruses II: a scoping review of Aedes vector competence, larval and adult bionomics and species distribution in Africa
Source: Parasit Vectors. 2026 Jul 28;19:460. doi: 10.1186/s13071-026-07552-5 (PMC13693134; doi:10.1186/s13071-026-07552-5)
Supplement: Supplementary file 7 [file 13071_2026_7552_MOESM7_ESM.docx]

| **Supplementary Table S2.5 Bionomics- Collection of resting adult *Aedes*** | | | | | |
| --- | --- | --- | --- | --- | --- |
| No. | Study | Publication Year | Country | Trapping method | Ref |
| 1 | Sabatinelli et al. | 1988 | Comoros | Pyrethrum spray catch (PSC) | [1] |
| 2 | Beier et al. | 1990 | Kenya | Pyrethrum spray catch (PSC), outdoor mechanical aspirator, mouth aspirator | [2] |
| 3 | Karch et al. | 1993 | DR Congo | Pyrethrum spray catch (PSC) | [3] |
| 4 | Thonnon et al. | 1998 | Senegal | Pyrethrum spray catch (PSC) | [4] |
| 5 | Akoua-Koffi et al. | 2001 | Côte d’Ivoire | Mouth aspirator | [5] |
| 6 | Mostafa et al. | 2002 | Egypt | Pyrethrum spray catch (PSC), indoor mechanical aspirator, mouth aspirator | [6] |
| 7 | Ba et al. | 2006 | Senegal | Outdoor mechanical aspirator | [7] |
| 8 | Sang et al. | 2008 | Comoros | Indoor mechanical aspirator | [8] |
| 9 | Oguoma et al. | 2008 | Nigeria | Sweep net | [9] |
| 10 | Muturi et al. | 2008 | Kenya | Pyrethrum spray catch (PSC) | [10] |
| 11 | Gould et al. | 2008 | Sudan | Pyrethrum spray catch (PSC), indoor mechanical aspirator | [11] |
| 12 | Delatte et al. | 2010 | La Réunion | Sweep net | [12] |
| 13 | Aribodor et al. | 2011 | Nigeria | Pyrethrum spray catch (PSC) | [13] |
| 14 | Konan et al. | 2011 | Côte d’Ivoire | Pyrethrum spray catch (PSC) | [14] |
| 15 | Kamgang et al. | 2012 | Cameroon | Mouth aspirator, sweep net | [15] |
| 16 | Raharimalala et al. | 2012 | Madagascar | Sweep net | [16] |
| 17 | Mwangangi et al. | 2012 | Kenya | Pyrethrum spray catch (PSC) | [17] |
| 18 | Ebenezer et al. | 2013 | Nigeria | Pyrethrum spray catch (PSC) | [18] |
| 19 | Diallo et al. | 2013 | Senegal | Outdoor mechanical aspirator | [19] |
| 20 | Masaninga et al. | 2014 | Zambia | Pyrethrum spray catch (PSC), outdoor & indoor mechanical aspirator | [20] |
| 21 | Yadouleton et al. | 2014 | Benin | Pyrethrum spray catch (PSC) | [21] |
| 22 | Yalwala et al. | 2015 | Kenya | Outdoor mechanical aspirator, resting boxes | [22] |
| 23 | Oduola et al. | 2016 | Nigeria | Pyrethrum spray catch (PSC) | [23] |
| 24 | Arum et al. | 2016 | Kenya | Outdoor mechanical aspirator | [24] |
| 25 | Lutomiah et al. | 2016 | Kenya | Indoor mechanical aspirator, resting boxes | [25] |
| 26 | Mohamed et al. | 2017 | Sudan | Pyrethrum spray catch (PSC), mouth aspirator | [26] |
| 27 | Agha et al. | 2017 | Kenya | Outdoor mechanical aspirator | [27] |
| 28 | Ndenga et al. | 2017 | Kenya | Outdoor & indoor mechanical aspirator | [28] |
| 29 | Iwashita et al. | 2018 | Kenya | Mouth aspirator | [29] |
| 30 | Labbo et al. | 2019 | Niger | Pyrethrum spray catch (PSC) | [30] |
| 31 | Thabet et al. | 2019 | Togo | Pyrethrum spray catch (PSC) | [31] |
| 32 | Agwu et al. | 2019 | Nigeria | Pyrethrum spray catch (PSC) | [32] |
| 33 | Mulchandani et al. | 2019 | Ethiopia | Outdoor & indoor mechanical aspirator | [33] |
| 34 | Agha et al. | 2019 | Kenya | Outdoor & indoor mechanical aspirator | [34] |
| 35 | Gneme et al. | 2019 | Burkina Faso | Mouth aspirator | [35] |
| 36 | Nebinur et al. | 2019 | Eritrea | Mouth aspirator | [36] |
| 37 | Karuitha et al. | 2019 | Kenya | Resting boxes | [37] |
| 38 | Irikannu et al. | 2020 | Nigeria | Pyrethrum spray catch (PSC) | [38] |
| 39 | Diallo et al. | 2020 | Senegal | Outdoor & indoor mechanical aspirator | [39] |
| 40 | Namountougou et al. | 2020 | Burkina Faso | Outdoor mechanical aspirator | [40] |
| 41 | Eastwood et al. | 2020 | Kenya | Mouth aspirator, resting boxes | [41] |
| 42 | Mayi et al. | 2020 | Cameroon | Sweep net | [42] |
| 43 | Waldetensai et al. | 2021 | Ethiopia | Mouth aspirator | [43] |
| 44 | Hamaidia et al. | 2021 | Algeria | Mouth aspirator | [44] |
| 45 | Onwuzulike et al. | 2021 | Nigeria | Pyrethrum spray catch (PSC) | [45] |
| 46 | Gutu et al. | 2021 | Ethiopia | Pyrethrum spray catch (PSC), mouth aspirator | [46] |
| 47 | Kampango et al. | 2021 | Tanzania | Outdoor & indoor mechanical aspirator | [47] |
| 48 | Diouf et al. | 2021 | Senegal | Outdoor & indoor mechanical aspirator | [48] |
| 49 | Owusu-Asenso et al. | 2022 | Ghana | Outdoor & indoor mechanical aspirator | [49] |
| 50 | Badolo et al. | 2022 | Burkina Faso | Outdoor & indoor mechanical aspirator | [50] |
| 51 | Sene et al. | 2022 | Senegal | Outdoor & indoor mechanical aspirator | [51] |
| 52 | Djiappi-Tchamen et al. | 2022 | Cameroon | Sweep net | [52] |

**References**

1.	Sabatinelli G, Majori G: **Utilisation de *Poecilia reticulata* dans la lutte contre le paludisme dans la R.F.I des Comores.** *Parassitologia* 1988, **30**.

2.	Beier JC, Odago WO, Onyango FK, Asiago CM, Koech DK, Roberts CR: **Relative abundance and blood feeding behavior of nocturnally active culicine mosquitoes in western Kenya.** *J Am Mosq Control Assoc* 1990, **6:**207-212.

3.	Karch S, Asidi N, Manzambi ZM, Salaun JJ: **[The culicidian fauna and its nuisance in Kinshasha (Zaire)].** *Bull Soc Pathol Exot* 1993, **86:**68-75.

4.	Thonnon J, Spiegel A, Diallo M, Sylla R, Fall A, Mondo M, Fontenille D: **Yellow fever outbreak in Kaffrine, Senegal 1996: epidemiological and entomological findings.** *Trop Med Int Health* 1998, **3:**872-877.

5.	Akoua-Koffi C, Diarrassouba S, Bénié VB, Ngbichi JM, Bozoua T, Bosson A, Akran V, Carnevale P, Ehouman A: **[Investigation surrounding a fatal case of yellow fever in Côte d'Ivoire in 1999].** *Bull Soc Pathol Exot* 2001, **94:**227-230.

6.	Mostafa AA, Allam KA, Osman MZ: **Mosquito species and their densities in some Egyptian governorates.** *J Egypt Soc Parasitol* 2002, **32:**9-20.

7.	Ba Y, Diallo D, Dia I, Diallo M: **[Feeding pattern of Rift Valley Fever virus vectors in Senegal. Implications in the disease epidemiology].** *Bull Soc Pathol Exot* 2006, **99:**283-289.

8.	Sang RC, Ahmed O, Faye O, Kelly CL, Yahaya AA, Mmadi I, Toilibou A, Sergon K, Brown J, Agata N, et al: **Entomologic investigations of a chikungunya virus epidemic in the Union of the Comoros, 2005.** *Am J Trop Med Hyg* 2008, **78:**77-82.

9.	Oguoma V, Ikpeze O: **Species composition and abundance of mosquitoes of a tropical irrigation ecosystem.** *Animal Research International* 2008.

10.	Muturi EJ, Muriu S, Shililu J, Mwangangi JM, Jacob BG, Mbogo C, Githure J, Novak RJ: **Blood-feeding patterns of Culex quinquefasciatus and other culicines and implications for disease transmission in Mwea rice scheme, Kenya.** *Parasitol Res* 2008, **102:**1329-1335.

11.	Gould LH, Osman MS, Farnon EC, Griffith KS, Godsey MS, Karch S, Mulenda B, El Kholy A, Grandesso F, de Radiguès X, et al: **An outbreak of yellow fever with concurrent chikungunya virus transmission in South Kordofan, Sudan, 2005.** *Trans R Soc Trop Med Hyg* 2008, **102:**1247-1254.

12.	Delatte H, Desvars A, Bouétard A, Bord S, Gimonneau G, Vourc'h G, Fontenille D: **Blood-feeding behavior of Aedes albopictus, a vector of Chikungunya on La Réunion.** *Vector Borne Zoonotic Dis* 2010, **10:**249-258.

13.	Aribodor D, Ikpeze O, Onyido A, Okoye C: **Survey of indoor adult malaria vectors and challenges of using long lasting insecticide-treated nets in malaria control in Awka-Etiti, Anambra State, Nigeria.** *Nigerian Journal of Parasitology* 2011.

14.	Konan YL, Fofana D, Coulibaly ZI, Diallo A, Koné AB, Doannio JM, Ekra KD, Odéhouri-Koudou P: **[Entomological investigations conducted around ten cases of yellow fever in 2009 in the Denguélé sanitary region, Côte-d'Ivoire].** *Bull Soc Pathol Exot* 2011, **104:**296-302.

15.	Kamgang B, Nchoutpouen E, Simard F, Paupy C: **Notes on the blood-feeding behavior of Aedes albopictus (Diptera: Culicidae) in Cameroon.** *Parasit Vectors* 2012, **5:**57.

16.	Raharimalala FN, Ravaomanarivo LH, Ravelonandro P, Rafarasoa LS, Zouache K, Tran-Van V, Mousson L, Failloux AB, Hellard E, Moro CV, et al: **Biogeography of the two major arbovirus mosquito vectors, Aedes aegypti and Aedes albopictus (Diptera, Culicidae), in Madagascar.** *Parasit Vectors* 2012, **5:**56.

17.	Mwangangi JM, Midega J, Kahindi S, Njoroge L, Nzovu J, Githure J, Mbogo CM, Beier JC: **Mosquito species abundance and diversity in Malindi, Kenya and their potential implication in pathogen transmission.** *Parasitol Res* 2012, **110:**61-71.

18.	Ebenezer A, Ben H, Enaregha E: **Atial distribution and indoor-resting density of mosquito species in the lowland rainforest of Bayelsa state, Nigeria.** *International Journal of Tropical Medicine* 2013.

19.	Diallo D, Chen R, Diagne CT, Ba Y, Dia I, Sall AA, Weaver SC, Diallo M: **Bloodfeeding patterns of sylvatic arbovirus vectors in southeastern Senegal.** *Trans R Soc Trop Med Hyg* 2013, **107:**200-203.

20.	Masaninga F, Muleba M, Masendu H, Songolo P, Mweene-Ndumba I, Mazaba-Liwewe ML, Kamuliwo M, Ameneshewa B, Siziya S, Babaniyi OA: **Distribution of yellow fever vectors in Northwestern and Western Provinces, Zambia.** *Asian Pac J Trop Med* 2014, **7S1:**S88-92.

21.	Yadouleton A, Agbanrin R, Vodounon C, Padonou G, Badirou K, Attolou R, Ursins F, Zola J, Allagbe H, Akogbeto M: **Seasonal distribution of Aedes aegypti in southern Benin: a risk of dengue virus transmission to urban populations.** *International Journal of Innovation and Applied Studies* 2014.

22.	Yalwala S, Clark J, Oullo D, Ngonga D, Abuom D, Wanja E, Bast J: **Comparative efficacy of existing surveillance tools for Aedes aegypti in Western Kenya.** *J Vector Ecol* 2015, **40:**301-307.

23.	Oduola A, Obembe A, Adelaja O, Ande A: **Surveillance and insecticide susceptibility status of Culicine mosquitoes in selected communities utilizing long-lasting insecticidal nets in Kwara State, Nigeria.** *Animal Research International* 2016, **13**.

24.	Arum SO, Weldon CW, Orindi B, Tigoi C, Musili F, Landmann T, Tchouassi DP, Affognon HD, Sang R: **Plant resting site preferences and parity rates among the vectors of Rift Valley Fever in northeastern Kenya.** *Parasit Vectors* 2016, **9:**310.

25.	Lutomiah J, Barrera R, Makio A, Mutisya J, Koka H, Owaka S, Koskei E, Nyunja A, Eyase F, Coldren R, Sang R: **Dengue Outbreak in Mombasa City, Kenya, 2013-2014: Entomologic Investigations.** *PLoS Negl Trop Dis* 2016, **10:**e0004981.

26.	Mohamed A, Ali A, Harbach R, Reeves R, Ibrahim K, AhmedMohmed M, Azrag R: **Aedes mosquitoes in the Republic of the Sudan, with dichotomous keys for the adult and larval stages.** *Journal of Natural History* 2017.

27.	Agha SB, Tchouassi DP, Bastos ADS, Sang R: **Dengue and yellow fever virus vectors: seasonal abundance, diversity and resting preferences in three Kenyan cities.** *Parasit Vectors* 2017, **10:**628.

28.	Ndenga BA, Mutuku FM, Ngugi HN, Mbakaya JO, Aswani P, Musunzaji PS, Vulule J, Mukoko D, Kitron U, LaBeaud AD: **Characteristics of Aedes aegypti adult mosquitoes in rural and urban areas of western and coastal Kenya.** *PLoS One* 2017, **12:**e0189971.

29.	Iwashita H, Higa Y, Futami K, Lutiali PA, Njenga SM, Nabeshima T, Minakawa N: **Mosquito arbovirus survey in selected areas of Kenya: detection of insect-specific virus.** *Trop Med Health* 2018, **46:**19.

30.	Labbo R, Doumma A, Mahamadou I, Arzika I, Soumana A, Kadri S, Idi I, Testa J: **Distribution and relative densities of Aedes aegypti in Niger.** *Med Sante Trop* 2019, **29:**47-54.

31.	Thabet HS, Fawaz EY, Badziklou K, Tag ElDin RA, Kaldas RM, Fahmy NT, Tamekloe TA, Kere-Banla A, Diclaro JW: **Preliminary Screening of Mosquito Spatial Distribution in Togo: With Special Focus on the Aedes (Diptera: Culicidae) Species.** *J Med Entomol* 2019, **56:**1154-1158.

32.	Agwu E, Isaac C, Igbinosa I: **Detection of yellow fever and dengue viruses in mosquitoes between 2014 and 2015 in Bayelsa and Benue states of Nigeria.** *Acta Entomologica Serbica* 2019, **24**.

33.	Mulchandani R, Massebo F, Bocho F, Jeffries CL, Walker T, Messenger LA: **A community-level investigation following a yellow fever virus outbreak in South Omo Zone, South-West Ethiopia.** *PeerJ* 2019, **7:**e6466.

34.	Agha SB, Tchouassi DP, Turell MJ, Bastos ADS, Sang R: **Entomological assessment of dengue virus transmission risk in three urban areas of Kenya.** *PLoS Negl Trop Dis* 2019, **13:**e0007686.

35.	Gneme A, Kabore J, Kpoda N, Gnoumou P, Mano K, Kabre G: **Diversity and abundance of mosquitoes in the city of Ouagadougou, Burkina Faso.** *International Journal of Entomology Research* 2019.

36.	Nebinur M, Bashir N, Dukeen M: **Presence, characterization and productivity profiles of Aedes mosquitoes (Diptera: Culicidae), in Barentu, Gash Barka Zone, Eritrea.** *International Journal of Mosquito Research* 2019.

37.	Karuitha M, Bargul J, Lutomiah J, Muriu S, Nzovu J, Sang R, Mwangangi J, Mbogo C: **Larval habitat diversity and mosquito species distribution along the coast of Kenya.** *Wellcome Open Res* 2019, **4:**175.

38.	Irikannu K, Onyido A, Nwankwo E, Umeanaeto P, Onwube O, Ogaraku J, Ezeagwuna D, Onyebueke A, Okoduwa A: **A survey of man-biting mosquito species in a tropical rainforest community in the Southeastern Nigeria.** *Environment and Ecology* 2020, **38**.

39.	Diallo D, Diallo M: **Resting behavior of Aedes aegypti in southeastern Senegal.** *Parasit Vectors* 2020, **13:**356.

40.	Namountougou M, Soma D, Kabore D, N'do S, Kientega M, Sawadogo J, Kagone T, Kania D, Sanou R, Sangare I, et al: **Characterisation of the breeding sites and insecticide resistance of Aedes aegypti population in the city of Bobo-Dioulaso, Burkina Faso.** *African Entomology* 2020, **28**.

41.	Eastwood G, Sang RC, Lutomiah J, Tunge P, Weaver SC: **Sylvatic Mosquito Diversity in Kenya-Considering Enzootic Ecology of Arboviruses in an Era of Deforestation.** *Insects* 2020, **11**.

42.	Mayi MPA, Bamou R, Djiappi-Tchamen B, Fontaine A, Jeffries CL, Walker T, Antonio-Nkondjio C, Cornel AJ, Tchuinkam T: **Habitat and Seasonality Affect Mosquito Community Composition in the West Region of Cameroon.** *Insects* 2020, **11**.

43.	Waldetensai A, Gemechu F, Kinfe E, Amare H, Hagos S, Teshome D, Hailemariam A, Nigatu W, Tadesse A, Keneni D, et al: **Aedes mosquito responses to control interventionsagainst the Chikungunya outbreak of Dire Dawa, Eastern Ethiopia.** *International Journal of Tropical Insect Science* 2021.

44.	Hamaidia K, Soltani N: **New report of Aedes albopictus in Souk Ahras, Northeast Algeria.** *Biodiversitas* 2021.

45.	Onwuzulike I, Onyebueke A, Irikannu K, Nzeukwu C, Ogbonna C, Nwangwu R, Ochiaka C: **Relative abundance and diversity of man-biting mosquito species before and after indoor residual spraying programme in Awka and Environs, Anambra State, Nigeria.** *Trends in Entomology* 2021, **17**.

46.	Gutu MA, Bekele A, Seid Y, Mohammed Y, Gemechu F, Woyessa AB, Tayachew A, Dugasa Y, Gizachew L, Idosa M, et al: **Another dengue fever outbreak in Eastern Ethiopia-An emerging public health threat.** *PLoS Negl Trop Dis* 2021, **15:**e0008992.

47.	Kampango A, Furu P, Sarath DL, Haji KA, Konradsen F, Schiøler KL, Alifrangis M, Weldon CW, Saleh F: **Targeted elimination of species-rich larval habitats can rapidly collapse arbovirus vector mosquito populations at hotel compounds in Zanzibar.** *Med Vet Entomol* 2021, **35:**523-533.

48.	Diouf B, Sene NM, Ndiaye EH, Gaye A, Ngom EHM, Gueye A, Seck F, Diagne CT, Dia I, Diallo M, Diallo D: **Resting Behavior of Blood-Fed Females and Host Feeding Preferences of Aedes aegypti (Diptera: Culicidae) Morphological Forms in Senegal.** *J Med Entomol* 2021, **58:**2467-2473.

49.	Owusu-Asenso CM, Mingle JAA, Weetman D, Afrane YA: **Spatiotemporal distribution and insecticide resistance status of Aedes aegypti in Ghana.** *Parasit Vectors* 2022, **15:**61.

50.	Badolo A, Sombié A, Yaméogo F, Wangrawa DW, Sanon A, Pignatelli PM, Viana M, Kanuka H, Weetman D, McCall PJ: **First comprehensive analysis of Aedes aegypti bionomics during an arbovirus outbreak in west Africa: Dengue in Ouagadougou, Burkina Faso, 2016-2017.** *PLoS Negl Trop Dis* 2022, **16:**e0010059.

51.	Sene NM, Diouf B, Gaye A, Ndiaye EH, Ngom EM, Gueye A, Seck F, Diagne CT, Dia I, Diallo D, Diallo M: **Blood Feeding Patterns of Aedes aegypti Populations in Senegal.** *Am J Trop Med Hyg* 2022, **106:**1402-1405.

52.	Djiappi-Tchamen B, Nana-Ndjangwo MS, Nchoutpouen E, Makoudjou I, Ngangue-Siewe IN, Talipouo A, Mayi MPA, Awono-Ambene P, Wondji C, Tchuinkam T, Antonio-Nkondjio C: **Mosquito Surveillance Using Ovitraps, Sweep Nets, and Biogent Traps in the City of Yaoundé, Cameroon.** *Insects* 2022, **13**.
