# Supplementary material for: Building resilience against the growing threat of arboviruses II: a scoping review of Aedes vector competence, larval and adult bionomics and species distribution in Africa
Source: Parasit Vectors. 2026 Jul 28;19:460. doi: 10.1186/s13071-026-07552-5 (PMC13693134; doi:10.1186/s13071-026-07552-5)
Supplement: Supplementary file 8 [file 13071_2026_7552_MOESM8_ESM.docx]

| **Supplementary Table 2.6 Bionomics- *Aedes* larval indices** | | | | | | |
| --- | --- | --- | --- | --- | --- | --- |
| No. | Study | Publication Year | Country | Mosquito Species | Larval indices reported | Ref |
| 1 | Merlin et al. | 1986 | Republic of Congo | *Ae. aegypti* | Container, Breteau | [1] |
| 2 | Nasidi et al. | 1989 | Nigeria | *Ae. aegypti* | Container, house, Breteau | [2] |
| 3 | Zeze et al. | 1994 | Côte d’Ivoire | *Ae. aegypti* | Container, house, Breteau | [3] |
| 4 | Rodier et al. | 1996 | Djibouti | *Ae. aegypti* | Container, house, Breteau | [4] |
| 5 | Thonnon et al. | 1998 | Senegal | *Ae. aegypti* & others | Container, Breteau | [5] |
| 6 | Anyanwu et al. | 1999 | Nigeria | *Ae. aegypti* | Container, house, Breteau | [6] |
| 7 | Diarrassouba et al. | 2000 | Côte d’Ivoire | *Ae. aegypti* & others | Container, house, Breteau | [7] |
| 8 | Adebote et al. | 2006 | Nigeria | *Ae. aegypti* | Container, house, Breteau | [8] |
| 9 | Midega et al. | 2006 | Kenya | *Ae. aegypti* | Pupal | [9] |
| 10 | Appawu et al. | 2006 | Ghana | *Aedes spp.* | Container, house, Breteau | [10] |
| 11 | Dehecq et al. | 2008 | La Réunion | *Ae. albopictus* | House, Breteau | [11] |
| 12 | Sang et al. | 2008 | Comoros | *Ae. aegypti* | Container, house, Breteau | [12] |
| 13 | Tun-Lin et al. | 2009 | Kenya | *Ae. aegypti* | Pupal | [13] |
| 14 | Kamgang et al. | 2010 | Cameroon | *Ae. aegypti, Ae. albopictus* | House, Breteau, pupal | [14] |
| 15 | Konan et al. | 2011 | Côte d’Ivoire | *Ae. aegypti* & others | Container, house, Breteau | [15] |
| 16 | Seidahmed et al. | 2012 | Sudan | *Ae. aegypti* | Container, house, Breteau, pupal | [16] |
| 17 | Kamgang et al. | 2013 | Cameroon | *Ae. aegypti, Ae. albopictus* | Container, house, Breteau, pupal | [17] |
| 18 | Diallo et al. | 2013 | Senegal | *Ae. aegypti* & others | Container, house, Breteau | [18] |
| 19 | Kone et al. | 2013 | Côte d’Ivoire | *Ae. aegypti* & others | Container, house, Breteau | [19] |
| 20 | Konan et al. | 2014 | Côte d’Ivoire | *Ae. aegypti* & others | Container, house, Breteau | [20] |
| 21 | Masaninga et al. | 2014 | Zambia | *Ae. aegypti* | Container, Breteau | [21] |
| 22 | Getachew et al. | 2015 | Ethiopia | *Ae. aegypti* | Container, house, Breteau | [22] |
| 23 | Suzuki-Takashi et al. | 2016 | Ghana | *Ae. aegypti, Ae. albopictus* | Container, house, Breteau | [23] |
| 24 | Usman et al. | 2016 | Eritrea | *Ae. aegypti* | Container, house, Breteau | [24] |
| 25 | Lutomiah et al. | 2016 | Kenya | *Ae. aegypti*, *Ae. tricholabis* | Container, house, Breteau | [25] |
| 26 | Mboera et al. | 2016 | Tanzania | *Ae. aegypti* | Container, house, Breteau, pupal | [26] |
| 27 | Kone et al. | 2018 | Côte d’Ivoire | *Ae. aegypti* | Container, house, Breteau | [27] |
| 28 | Konongoi et al. | 2018 | Kenya | *Ae. aegypti* | Container, house, Breteau | [28] |
| 29 | Bakr et al. | 2018 | Sudan | *Aedes spp.* | Container, house, Breteau, pupal | [29] |
| 30 | Ouedraogo et al. | 2018 | Burkina Faso | *Ae. aegypti* | Container, house, Breteau, pupal | [30] |
| 31 | Abilio et al. | 2018 | Mozambique | *Ae. aegypti, Ae. albopictus* | Container | [31] |
| 32 | Eshag et al. | 2019 | Sudan | *Ae. aegypti* & others | Container, house, Breteau, pupal | [32] |
| 33 | Fofana et al. | 2019 | Côte d’Ivoire | *Ae. aegypti* | Container, house, Breteau | [33] |
| 34 | Ouattara et al. | 2019 | Burkina Faso | *Ae. aegypti, Ae. vittatus* | Container, house, Breteau | [34] |
| 35 | Aikpon et al. | 2019 | Benin | *Ae. aegypti* | Container, house, Breteau | [35] |
| 36 | Mulchandani et al. | 2019 | Ethiopia | *Ae. simpsoni* | Container, house, Breteau, pupal | [36] |
| 37 | Nebinur et al. | 2019 | Eritrea | *Ae. aegypti* | Container, house, Breteau | [37] |
| 38 | Nwachukwu et al. | 2020 | Nigeria | *Ae. aegypti* & others | Container, house, Breteau | [38] |
| 39 | Kahamba et al. | 2020 | Tanzania | *Ae. aegypti* | Container, house, Breteau | [39] |
| 40 | Kudom | 2020 | Ghana | *Ae. aegypti* | Container, house, Breteau | [40] |
| 41 | Saleh et al. | 2020 | Tanzania | *Ae. aegypti* | Container, house, Breteau, pupal | [41] |
| 42 | Bonnet et al. | 2020 | Burkina Faso | *Ae. aegypti* | Container, house, Breteau, pupal | [42] |
| 43 | Forsyth et al. | 2020 | Kenya | *Ae. aegypti* | Container, house, Breteau | [43] |
| 44 | Leal et al. | 2020 | Cabo Verde | *Ae. aegypti* | Container, house, Breteau | [44] |
| 45 | Wilson-Bahun et al. | 2020 | Republic of Congo | *Ae. aegypti, Ae. albopictus* | Container, house, Breteau | [45] |
| 46 | Kampango et al. | 2021 | Tanzania | *Ae. aegypti* & others | Container, pupal | [46] |
| 47 | Waldetensai et al. | 2021 | Ethiopia | *Aedes spp.* | Container, house, Breteau, pupal | [47] |
| 48 | Yenus et al. | 2021 | Eritrea | *-* | Container, house, Breteau, pupal | [48] |
| 49 | Wat’senga et al. | 2021 | DR Congo | *Ae. aegypti, Ae. albopictus* | Container, house, Breteau, pupal | [49] |
| 50 | Yenus et al. | 2021 | Eritrea | *Ae. aegypti, Ae. aegypti formosus* | Container, house, Breteau | [50] |
| 51 | Gutu et al. | 2021 | Ethiopia | *Ae. aegypti, Ae. africanus* | Container, house, Breteau | [51] |
| 52 | Hamid et al. | 2021 | Sudan | *Ae. aegypti, Ae. vittatus* | Container, house, Breteau, pupal | [52] |
| 53 | Karisa et al. | 2021 | Kenya | *Ae. aegypti* | Container, house, Breteau | [53] |
| 54 | Mbanzulu et al. | 2022 | DR Congo | *Ae. aegypti, Ae. albopictus* | Container | [54] |
| 55 | Mekuriaw et al. | 2022 | Ethiopia | *Ae. aegypti* | Container, house, Breteau | [55] |
| 56 | Ngingo et al. | 2022 | Tanzania | *Ae. aegypti* | Container, house, Breteau | [56] |
| 57 | Yao et al. | 2022 | Côte d’Ivoire | *Ae. aegypti* | Container, house, Breteau | [57] |
| 58 | Diakaridia et al. | 2022 | Côte d’Ivoire | *Ae. aegypti* | Container, house, Breteau | [58] |
| 59 | Guindo-Coulibaly et al. | 2022 | Côte d’Ivoire | *Ae. aegypti* | Container, house, Breteau | [59] |
| 60 | Badolo et al. | 2022 | Burkina Faso | *Ae. aegypti* & others | Container, house, Breteau, pupal | [60] |
| 61 | Ouedraogo et al. | 2022 | Burkina Faso | *Ae. aegypti* | Container, house, Breteau, pupal | [61] |

**References**

1.	Merlin M, Josse R, Kouka-Bemba D, Meunier D, Senga J, Simonkovich E, Malonga JR, Manoukou F, Georges AJ: **[Evaluation of immunological and entomotological indices of yellow fever in Pointe-Noire, People's Republic of Congo].** *Bull Soc Pathol Exot Filiales* 1986, **79:**199-206.

2.	Nasidi A, Monath TP, DeCock K, Tomori O, Cordellier R, Olaleye OD, Harry TO, Adeniyi JA, Sorungbe AO, Ajose-Coker AO: **Urban yellow fever epidemic in western Nigeria, 1987.** *Trans R Soc Trop Med Hyg* 1989, **83:**401-406.

3.	Zeze DG, Koffi AA: **[Stegomyian indices and epidemiological status of yellow fever in a rural area of the Ivory Coast].** *Med Trop (Mars)* 1994, **54:**324-330.

4.	Rodier GR, Gubler DJ, Cope SE, Cropp CB, Soliman AK, Polycarpe D, Abdourhaman MA, Parra JP, Maslin J, Arthur RR: **Epidemic dengue 2 in the city of Djibouti 1991-1992.** *Trans R Soc Trop Med Hyg* 1996, **90:**237-240.

5.	Thonnon J, Spiegel A, Diallo M, Sylla R, Fall A, Mondo M, Fontenille D: **Yellow fever outbreak in Kaffrine, Senegal 1996: epidemiological and entomological findings.** *Trop Med Int Health* 1998, **3:**872-877.

6.	Anyanwu G, Iwuala M: **Mosquito breeding sites: distribution and relative abundance of species in the Jos Plateau, Nigeria.** *Medical Entomology and Zoology* 1999.

7.	Diarrassouba S, Herve J, Dossou-Yovo J, Carnevale P: **Water stocking habits and seasonal variations in biting rates of Aedes (Stegomyia) aegypti Linnaeus (Diptera: Culicidae) in a sub-sudanian village of Cote d'Ivoire.** *Research and reviews in parasitology* 2000.

8.	Adebote A, Oniye J, Ndams S, Nache K: **The breeding of mosquitoes (Diptera: Culicidae) in peridomestic containers and implication in yellow fever transmission in villages around Zaria, Northern Nigeria.** *Journal of Entomology* 2006.

9.	Midega JT, Nzovu J, Kahindi S, Sang RC, Mbogo C: **Application of the pupal/demographic-survey methodology to identify the key container habitats of Aedes aegypti (L.) in Malindi district, Kenya.** *Ann Trop Med Parasitol* 2006, **100 Suppl 1:**S61-S72.

10.	Appawu M, Dadzie S, Abdul H, Asmah H, Boakye D, Wilson M, Ofori-Adjei D: **Surveillance of viral haemorrhagic fevers in ghana: entomological assessment of the risk of transmission in the northern regions.** *Ghana Med J* 2006, **40:**137-141.

11.	Dehecq J-S, Fohr G, Thiria J: **Plan de lutte contre Aedes albopictus pendant l’épidémie de chikungunyaà La Réunion en 2005-2007.** Bulletin Epidemiologique Hebdomadaire2008.

12.	Sang RC, Ahmed O, Faye O, Kelly CL, Yahaya AA, Mmadi I, Toilibou A, Sergon K, Brown J, Agata N, et al: **Entomologic investigations of a chikungunya virus epidemic in the Union of the Comoros, 2005.** *Am J Trop Med Hyg* 2008, **78:**77-82.

13.	Tun-Lin W, Lenhart A, Nam VS, Rebollar-Téllez E, Morrison AC, Barbazan P, Cote M, Midega J, Sanchez F, Manrique-Saide P, et al: **Reducing costs and operational constraints of dengue vector control by targeting productive breeding places: a multi-country non-inferiority cluster randomized trial.** *Trop Med Int Health* 2009, **14:**1143-1153.

14.	Kamgang B, Happi JY, Boisier P, Njiokou F, Hervé JP, Simard F, Paupy C: **Geographic and ecological distribution of the dengue and chikungunya virus vectors Aedes aegypti and Aedes albopictus in three major Cameroonian towns.** *Med Vet Entomol* 2010, **24:**132-141.

15.	Konan YL, Fofana D, Coulibaly ZI, Diallo A, Koné AB, Doannio JM, Ekra KD, Odéhouri-Koudou P: **[Entomological investigations conducted around ten cases of yellow fever in 2009 in the Denguélé sanitary region, Côte-d'Ivoire].** *Bull Soc Pathol Exot* 2011, **104:**296-302.

16.	Seidahmed OM, Siam HA, Soghaier MA, Abubakr M, Osman HA, Abd Elrhman LS, Elmagbol B, Velayudhan R: **Dengue vector control and surveillance during a major outbreak in a coastal Red Sea area in Sudan.** *East Mediterr Health J* 2012, **18:**1217-1224.

17.	Kamgang B, Ngoagouni C, Manirakiza A, Nakouné E, Paupy C, Kazanji M: **Temporal patterns of abundance of Aedes aegypti and Aedes albopictus (Diptera: Culicidae) and mitochondrial DNA analysis of Ae. albopictus in the Central African Republic.** *PLoS Negl Trop Dis* 2013, **7:**e2590.

18.	Diallo M, Tall A, Dia I, Ba Y, Sarr FD, Ly A, Faye J, Badiane A, Diakhaby G, Faye P, et al: **Yellow fever outbreak in central part of Senegal 2002: epidemiological findings.** *Journal of Public Health and Epidemiology* 2013, **5-7:**291-296.

19.	Kone AB, Konan YL, Coulibaly ZI, Fofana D, Guindo-Coulibaly N, Diallo M, Doannio JM, Ekra KD, Odehouri-Koudou P: **[Entomological evaluation of the risk of urban outbreak of yellow fever in 2008 in Abidjan, Côte d'Ivoire].** *Med Sante Trop* 2013, **23:**66-71.

20.	Konan YL, Coulibaly ZI, Allali KB, Tétchi SM, Koné AB, Coulibaly D, Ekra KD, Doannio JM, Oudéhouri-Koudou P: **[Management of the yellow fever epidemic in 2010 in Séguéla (Côte d'Ivoire): value of multidisciplinary investigation].** *Sante Publique* 2014, **26:**859-867.

21.	Masaninga F, Muleba M, Masendu H, Songolo P, Mweene-Ndumba I, Mazaba-Liwewe ML, Kamuliwo M, Ameneshewa B, Siziya S, Babaniyi OA: **Distribution of yellow fever vectors in Northwestern and Western Provinces, Zambia.** *Asian Pac J Trop Med* 2014, **7S1:**S88-92.

22.	Getachew D, Tekie H, Gebre-Michael T, Balkew M, Mesfin A: **Breeding Sites of Aedes aegypti: Potential Dengue Vectors in Dire Dawa, East Ethiopia.** *Interdiscip Perspect Infect Dis* 2015, **2015:**706276.

23.	Suzuki T, Osei JH, Sasaki A, Adimazoya M, Appawu M, Boakye D, Ohta N, Dadzie S: **Risk of transmission of viral haemorrhagic fevers and the insecticide susceptibilitystatus of.** *Ghana Med J* 2016, **50:**136-141.

24.	Usman A, Ball JD, Rojas DP, Berhane A, Ghebrat Y, Mebrahtu G, Gebresellasie A, Zehaie A, Mufunda J, Liseth O, et al: **Dengue fever outbreaks in Eritrea, 2005-2015: A case for strengthening surveillance, control and reporting.** *Glob Health Res Policy* 2016, **1:**17.

25.	Lutomiah J, Barrera R, Makio A, Mutisya J, Koka H, Owaka S, Koskei E, Nyunja A, Eyase F, Coldren R, Sang R: **Dengue Outbreak in Mombasa City, Kenya, 2013-2014: Entomologic Investigations.** *PLoS Negl Trop Dis* 2016, **10:**e0004981.

26.	Mboera LE, Mweya CN, Rumisha SF, Tungu PK, Stanley G, Makange MR, Misinzo G, De Nardo P, Vairo F, Oriyo NM: **The Risk of Dengue Virus Transmission in Dar es Salaam, Tanzania during an Epidemic Period of 2014.** *PLoS Negl Trop Dis* 2016, **10:**e0004313.

27.	Kone B, Konan L, Fofana D, Koffi A, Coulibaly D, Benie J: **Entomological evaluation of the risk of spread ofthe dengue 3 epidemic in the health district ofCocody (Abidjan, Côte d’Ivoire).** *International Journal of Mosquito Research* 2018, **5 (5):**19-24.

28.	Konongoi SL, Nyunja A, Ofula V, Owaka S, Koka H, Koskei E, Eyase F, Langat D, Mancuso J, Lutomiah J, Sang R: **Human and entomologic investigations of chikungunya outbreak in Mandera, Northeastern Kenya, 2016.** *PLoS One* 2018, **13:**e0205058.

29.	Bakr A, Bashir N, Kehail M: **Spatio-temporal distribution of Aedes mosquitoes (Diptera: Culicidae), in Alfasher Locality North Darfur State, Sudan.** *International Journal of Mosquito Research* 2018.

30.	Ouédraogo S, Benmarhnia T, Bonnet E, Somé PA, Barro AS, Kafando Y, Soma DD, Dabiré RK, Saré D, Fournet F, Ridde V: **Evaluation of Effectiveness of a Community-Based Intervention for Control of Dengue Virus Vector, Ouagadougou, Burkina Faso.** *Emerg Infect Dis* 2018, **24:**1859-1867.

31.	Abílio AP, Abudasse G, Kampango A, Candrinho B, Sitoi S, Luciano J, Tembisse D, Sibindy S, de Almeida APG, Garcia GA, et al: **Distribution and breeding sites of Aedes aegypti and Aedes albopictus in 32 urban/peri-urban districts of Mozambique: implication for assessing the risk of arbovirus outbreaks.** *PLoS Negl Trop Dis* 2018, **12:**e0006692.

32.	Eshag O, Bashir N, Dukeen M: **Prevalence, habitat and productivity profiles of*Aedes* mosquitoes (Diptera: Culicidae) in Sennarstate, Sudan.** 2019, **6**.

33.	Fofana D, Beugré JMV, Yao-Acapovi GL, Lendzele SS: **Risk of Dengue Transmission in Cocody (Abidjan, Ivory Coast).** *J Parasitol Res* 2019, **2019:**4914137.

34.	Ouattara LPE, Sangaré I, Namountougou M, Hien A, Ouari A, Soma DD, Kassié D, Diabaté A, Gnankiné O, Bonnet E, et al: **Surveys of Arboviruses Vectors in Four Cities Stretching Along a Railway Transect of Burkina Faso: Risk Transmission and Insecticide Susceptibility Status of Potential Vectors.** *Front Vet Sci* 2019, **6:**140.

35.	Aikpon R, Klotoe J, Dramane G, Brettenny M, Lawani Y: **Larval breeding characteristics and distribution of Aedes mosquito species in the economic capital of Benin: A public health concern.** *International Journal of Entomology Research* 2019.

36.	Mulchandani R, Massebo F, Bocho F, Jeffries CL, Walker T, Messenger LA: **A community-level investigation following a yellow fever virus outbreak in South Omo Zone, South-West Ethiopia.** *PeerJ* 2019, **7:**e6466.

37.	Nebinur M, Bashir N, Dukeen M: **Presence, characterization and productivity profiles of Aedes mosquitoes (Diptera: Culicidae), in Barentu, Gash Barka Zone, Eritrea.** *International Journal of Mosquito Research* 2019.

38.	Nwachukwu WE, Yusuff H, Nwangwu U, Okon A, Ogunniyi A, Imuetinyan-Clement J, Besong M, Ayo-Ajayi P, Nikau J, Baba A, et al: **The response to re-emergence of yellow fever in Nigeria, 2017.** *Int J Infect Dis* 2020, **92:**189-196.

39.	Kahamba NF, Limwagu AJ, Mapua SA, Msugupakulya BJ, Msaky DS, Kaindoa EW, Ngowo HS, Okumu FO: **Habitat characteristics and insecticide susceptibility of Aedes aegypti in the Ifakara area, south-eastern Tanzania.** *Parasit Vectors* 2020, **13:**53.

40.	Kudom AA: **Entomological surveillance to assess potential outbreak of Aedes-borne arboviruses and insecticide resistance status of Aedes aegypti from Cape Coast, Ghana.** *Acta Trop* 2020, **202:**105257.

41.	Saleh F, Kitau J, Konradsen F, Kampango A, Abassi R, Schiøler KL: **Epidemic risk of arboviral diseases: Determining the habitats, spatial-temporal distribution, and abundance of immature Aedes aegypti in the Urban and Rural areas of Zanzibar, Tanzania.** *PLoS Negl Trop Dis* 2020, **14:**e0008949.

42.	Bonnet E, Fournet F, Benmarhnia T, Ouedraogo S, Dabiré R, Ridde V: **Impact of a community-based intervention on Aedes aegypti and its spatial distribution in Ouagadougou, Burkina Faso.** *Infect Dis Poverty* 2020, **9:**61.

43.	Forsyth JE, Mutuku FM, Kibe L, Mwashee L, Bongo J, Egemba C, Ardoin NM, LaBeaud AD: **Source reduction with a purpose: Mosquito ecology and community perspectives offer insights for improving household mosquito management in coastal Kenya.** *PLoS Negl Trop Dis* 2020, **14:**e0008239.

44.	Leal SDV, Fernandes Varela IB, Lopes Gonçalves AAL, Sousa Monteiro DD, Ramos de Sousa CM, Lima Mendonça MDL, De Pina AJ, Alves MJ, Osório HC: **Abundance and Updated Distribution of.** *Int J Environ Res Public Health* 2020, **17**.

45.	Wilson-Bahun TA, Kamgang B, Lenga A, Wondji CS: **Larval ecology and infestation indices of two major arbovirus vectors, Aedes aegypti and Aedes albopictus (Diptera: Culicidae), in Brazzaville, the capital city of the Republic of the Congo.** *Parasit Vectors* 2020, **13:**492.

46.	Kampango A, Furu P, Sarath DL, Haji KA, Konradsen F, Schiøler KL, Alifrangis M, Saleh F, Weldon CW: **Risk factors for occurrence and abundance of Aedes aegypti and Aedes bromeliae at hotel compounds in Zanzibar.** *Parasit Vectors* 2021, **14:**544.

47.	Waldetensai A, Gemechu F, Kinfe E, Amare H, Hagos S, Teshome D, Hailemariam A, Nigatu W, Tadesse A, Keneni D, et al: **Aedes mosquito responses to control interventionsagainst the Chikungunya outbreak of Dire Dawa, Eastern Ethiopia.** *International Journal of Tropical Insect Science* 2021.

48.	Yenus M, Berhe M, Brhane M, Kiflemariam A, Sereke K: **Assessment the effectiveness of community based dengue and chikungunya intervention: Keren, Anseba zone Eritrea (2020).** *International Journal of Mosquito Research* 2021, **8**.

49.	Wat'senga Tezzo F, Fasine S, Manzambi Zola E, Marquetti MDC, Binene Mbuka G, Ilombe G, Mundeke Takasongo R, Smitz N, Bisset JA, Van Bortel W, Vanlerberghe V: **High Aedes spp. larval indices in Kinshasa, Democratic Republic of Congo.** *Parasit Vectors* 2021, **14:**92.

50.	Yenus M, Brhane M, Berhe M, Kiflemariam A, Sereke K: **Abundance, characterization and productivity profiles of Aedes mosquitoes (Diptera: Culicidae), in Keren, Elabered and Hagas, Anseba zone, Eritrea.** *International Journal of Mosquito Research* 2021.

51.	Gutu MA, Bekele A, Seid Y, Mohammed Y, Gemechu F, Woyessa AB, Tayachew A, Dugasa Y, Gizachew L, Idosa M, et al: **Another dengue fever outbreak in Eastern Ethiopia-An emerging public health threat.** *PLoS Negl Trop Dis* 2021, **15:**e0008992.

52.	Hamid H, Musa H, Ahmed A, Abdul Azeez T, Adam A, Abdel Malik M, El Tahir A, Kafy H, Azrag R: **Stegomyia indices of Aedes aquatic stages in El Geneina town, Western Sudan.** *East Mediterr Health J* 2021, **27:**1189-1196.

53.	Karisa J, Muriu S, Omuoyo D, Karia B, Ngari M, Nyamwaya D, Rono M, Warimwe G, Mwangangi J, Mbogo CM: **Urban Ecology of Arboviral Mosquito Vectors Along the Kenyan Coast.** *J Med Entomol* 2021, **58:**428-438.

54.	Mbanzulu KM, Wumba R, Mboera LEG, Kayembe JN, Engbu D, Bojabwa MM, Zanga JK, Misinzo G, Kimera SI: **Pattern of.** *Trop Med Infect Dis* 2022, **7**.

55.	Mekuriaw W, Kinde S, Kindu B, Mulualem Y, Hailu G, Gebresilassie A, Sisay C, Bekele F, Amare H, Wossen M, et al: **Epidemiological, Entomological, and Climatological Investigation of the 2019 Dengue Fever Outbreak in Gewane District, Afar Region, North-East Ethiopia.** *Insects* 2022, **13**.

56.	Ngingo BL, Mboera LEG, Chengula A, Machelle I, Makange MR, Msolla M, Mwanyika GO, Rugarabamu S, Misinzo G: **Aedes aegypti abundance, larval indices and risk for dengue virus transmission in Kinondoni district, Tanzania.** *Trop Med Health* 2022, **50:**1.

57.	Yao K, Konan Y, Coulibaly Z, Silue G, Coulibaly D, N'Goran K: **Larval ecology of Aedes mosquitoes and risk of arbovirus transmission at the port of San-pedro (Cote d'Ivoire).** *International Journal of Mosquito Research* 2022.

58.	Diakaridia F, Konan L, Kone B, Coulibaly D, Beugre J, Akaffou M, Diallo A, Benie B, Dosso M, N'Goran N: **Household water storage containers: Aedes aegypti larval breeding site and high risk of transmission of arboviruses in Abidjan, Cote d'Ivoire.** *International Journal of Biological and Chemical Sciences* 2022.

59.	Guindo-Coulibaly N, Kpan MDS, Adja AM, Kouadio AMN, Assouho KF, Zoh DD, Azongnibo KRM, Remoue F, Fournet F: **Seasonal variation and intra urban heterogeneity of the entomological risk of transmission of dengue and yellow fever in Abidjan, Côte d'Ivoire.** *Med Vet Entomol* 2022, **36:**329-337.

60.	Badolo A, Sombié A, Yaméogo F, Wangrawa DW, Sanon A, Pignatelli PM, Viana M, Kanuka H, Weetman D, McCall PJ: **First comprehensive analysis of Aedes aegypti bionomics during an arbovirus outbreak in west Africa: Dengue in Ouagadougou, Burkina Faso, 2016-2017.** *PLoS Negl Trop Dis* 2022, **16:**e0010059.

61.	Ouédraogo WM, Toé KH, Sombié A, Viana M, Bougouma C, Sanon A, Weetman D, McCall PJ, Kanuka H, Badolo A: **Impact of physicochemical parameters of Aedes aegypti breeding habitats on mosquito productivity and the size of emerged adult mosquitoes in Ouagadougou City, Burkina Faso.** *Parasit Vectors* 2022, **15:**478.
