# Supplementary material for: Building resilience against the growing threat of arboviruses II: a scoping review of Aedes vector competence, larval and adult bionomics and species distribution in Africa
Source: Parasit Vectors. 2026 Jul 28;19:460. doi: 10.1186/s13071-026-07552-5 (PMC13693134; doi:10.1186/s13071-026-07552-5)
Supplement: Supplementary file 9 [file 13071_2026_7552_MOESM9_ESM.docx]

| **Supplementary Table 2.7 Bionomics- *Aedes* larval characteristics** | | | | | |
| --- | --- | --- | --- | --- | --- |
| No. | Study | Publication Year | Country | Mosquito Species | Ref |
| 1 | Hougard et al. | 1985 | Côte d’Ivoire | *Ae. aegypti* | [1] |
| 2 | Merlin et al. | 1986 | Republic of Congo | *Ae. aegypti* | [2] |
| 3 | Zeze et al. | 1994 | Côte d’Ivoire | *Ae. aegypti* | [3] |
| 4 | Rodier et al. | 1996 | Djibouti | *Ae. aegypti* | [4] |
| 5 | Anyanwu et al. | 1999 | Nigeria | *Ae. aegypti* | [5] |
| 6 | Diarrassouba et al. | 2000 | Côte d’Ivoire | *Ae. aegypti* & others | [6] |
| 7 | Fontenille et al. | 2001 | Cameroon | *Ae. albopictus* | [7] |
| 8 | Akoua-Koffi et al. | 2001 | Côte d’Ivoire | *Ae. aegypti* | [8] |
| 9 | Simard et al. | 2005 | Cameroon | *Ae. aegypti, Ae. albopictus* | [9] |
| 10 | Mwangangi et al. | 2006 | Kenya | *Ae. aegypti* | [10] |
| 11 | Midega et al. | 2006 | Kenya | *Ae. aegypti* | [11] |
| 12 | Olayemi et al. | 2008 | Nigeria | *Ae. aegypti* | [12] |
| 13 | Dehecq et al. | 2008 | La Réunion | *Ae. albopictus* | [13] |
| 14 | Delatte et al. | 2008 | La Réunion | *Ae. albopictus* | [14] |
| 15 | Sang et al. | 2008 | Comoros | *Ae. aegypti* | [15] |
| 16 | Tun-Lin et al. | 2009 | Kenya | *Ae. aegypti* | [16] |
| 17 | Kamgang et al. | 2010 | Cameroon | *Ae. aegypti, Ae. albopictus* | [17] |
| 18 | Yaro et al. | 2010 | Burkina Faso | *Ae. aegypti* | [18] |
| 19 | Konan et al. | 2011 | Côte d’Ivoire | *Ae. aegypti* & others | [19] |
| 20 | Diallo et al. | 2012 | Senegal | *Ae. aegypti* | [20] |
| 21 | Seidahmed et al. | 2012 | Sudan | *Ae. aegypti* | [21] |
| 22 | Duarte et al. | 2013 | Cabo Verde | *Ae. aegypti* | [22] |
| 23 | Diallo et al. | 2013 | Senegal | *Ae. aegypti* & others | [23] |
| 24 | Kone et al. | 2013 | Côte d’Ivoire | *Ae. aegypti* & others | [24] |
| 25 | Kamgang et al. | 2013 | Cameroon | *Ae. aegypti, Ae. albopictus* | [25] |
| 26 | Idowu et al. | 2014 | Nigeria | *Ae. aegypti, Ae. albopictus* | [26] |
| 27 | Noden et al. | 2014 | Namibia | *Ae. aegypti* | [27] |
| 28 | Mint Lekweiry et al. | 2015 | Mauritania | *Ae. aegypti* | [28] |
| 29 | Getachew et al. | 2015 | Ethiopia | *Ae. aegypti* | [29] |
| 30 | Yalwala et al. | 2015 | Kenya | *Ae. aegypti, Ae. aegypti formosus* | [30] |
| 31 | Higa et al. | 2015 | Mozambique | *Ae. aegypti, Ae. aegypti formosus* | [31] |
| 32 | Lutomiah et al. | 2016 | Kenya | *Ae. aegypti*, *Ae. tricholabis* | [32] |
| 33 | Mboera et al. | 2016 | Tanzania | *Ae. aegypti* | [33] |
| 34 | Usman et al. | 2016 | Eritrea | *Ae. aegypti* | [34] |
| 35 | Ngugi et al. | 2017 | Kenya | *Ae. aegypti* | [35] |
| 36 | Kamgang et al. | 2017 | Cameroon | *Ae. aegypti, Ae. albopictus* | [36] |
| 37 | Mathias et al. | 2017 | Tanzania | *Ae. aegypti* | [37] |
| 38 | Mahgoub et al. | 2017 | Sudan | *Ae. aegypti* | [38] |
| 39 | Zahouli et al. | 2017 | Côte d’Ivoire | *Ae. aegypti* | [39] |
| 40 | Zahouli et al. | 2017 | Côte d’Ivoire | *Ae. aegypti* | [40] |
| 41 | Kone et al. | 2018 | Côte d’Ivoire | *Ae. aegypti* | [41] |
| 42 | Konongoi et al. | 2018 | Kenya | *Ae. aegypti* | [42] |
| 43 | Bakr et al. | 2018 | Sudan | *Aedes spp.* | [43] |
| 44 | Abilio et al. | 2018 | Mozambique | *Ae. aegypti, Ae. albopictus* | [44] |
| 45 | Mugabe et al. | 2018 | Mozambique | *Ae. aegypti* | [45] |
| 46 | Kamgang et al. | 2018 | Republic of Congo | *Ae. aegypti, Ae. albopictus* | [46] |
| 47 | Saleh et al. | 2018 | Tanzania | *Ae. aegypti* | [47] |
| 48 | Aikpon et al. | 2019 | Benin | *Ae. aegypti* | [48] |
| 49 | Ouattara et al. | 2019 | Burkina Faso | *Ae. aegypti, Ae. vittatus* | [49] |
| 50 | Eshag et al. | 2019 | Sudan | *Ae. aegypti* & others | [50] |
| 51 | Mulchandani et al. | 2019 | Ethiopia | *Ae. simpsoni* | [51] |
| 52 | Nebinur et al. | 2019 | Eritrea | *Ae. aegypti* | [52] |
| 53 | Tedjou et al. | 2019 | Cameroon | *Ae. aegypti, Ae. albopictus* | [53] |
| 54 | Kahamba et al. | 2020 | Tanzania | *Ae. aegypti* | [54] |
| 55 | Saleh et al. | 2020 | Tanzania | *Ae. aegypti* | [55] |
| 56 | Bonnet et al. | 2020 | Burkina Faso | *Ae. aegypti* | [56] |
| 57 | Forsyth et al. | 2020 | Kenya | *Ae. aegypti* | [57] |
| 58 | Leal et al. | 2020 | Cabo Verde | *Ae. aegypti* | [58] |
| 59 | Wilson-Bahun et al. | 2020 | Republic of Congo | *Ae. aegypti, Ae. albopictus* | [59] |
| 60 | Hondt et al. | 2020 | Cameroon | *Ae. aegypti, Ae. albopictus* | [60] |
| 61 | Diouf et al. | 2020 | Senegal | *Ae. aegypti* | [61] |
| 62 | Kudom | 2020 | Ghana | *Ae. aegypti* | [62] |
| 63 | Mathias et al. | 2020 | Nigeria | *Ae. aegypti* | [63] |
| 64 | Kampango et al. | 2021 | Tanzania | *Ae. aegypti* & others | [64] |
| 65 | Waldetensai et al. | 2021 | Ethiopia | *Aedes spp.* | [65] |
| 66 | Wat’senga et al. | 2021 | DR Congo | *Ae. aegypti, Ae. albopictus* | [66] |
| 67 | Yenus et al. | 2021 | Eritrea | *Ae. aegypti, Ae. aegypti formosus* | [67] |
| 68 | Gutu et al. | 2021 | Ethiopia | *Ae. aegypti, Ae. africanus* | [68] |
| 69 | Hamid et al. | 2021 | Sudan | *Ae. aegypti, Ae. vittatus* | [69] |
| 70 | Kampango et al. | 2021 | Tanzania | *Ae. aegypti* | [70] |
| 71 | Irikannu et al. | 2021 | Nigeria | *Ae. aegypti, Ae. albopictus* | [71] |
| 72 | Ouattara et al. | 2021 | Côte d’Ivoire | *Ae. aegypti* | [72] |
| 73 | Mbanzulu et al. | 2022 | DR Congo | *Ae. aegypti, Ae. albopictus* | [73] |
| 74 | Mekuriaw et al. | 2022 | Ethiopia | *Ae. aegypti* | [74] |
| 75 | Ngingo et al. | 2022 | Tanzania | *Ae. aegypti* | [75] |
| 76 | Yao et al. | 2022 | Côte d’Ivoire | *Ae. aegypti* | [76] |
| 77 | Diakaridia et al. | 2022 | Côte d’Ivoire | *Ae. aegypti* | [77] |
| 78 | Guindo-Coulibaly et al. | 2022 | Côte d’Ivoire | *Ae. aegypti* | [78] |
| 79 | Badolo et al. | 2022 | Burkina Faso | *Ae. aegypti* & others | [79] |
| 80 | Ouedraogo et al. | 2022 | Burkina Faso | *Ae. aegypti* | [80] |
| 81 | Owusu-Asenso et al. | 2022 | Ghana | *Ae. aegypti* | [81] |
| 82 | Yougang et al. | 2022 | Cameroon | *Ae. aegypti, Ae. albopictus* | [82] |
| 83 | Silue et al. | 2022 | Côte d’Ivoire | *Aedes spp.* | [83] |
| 84 | Eze et al. | 2022 | Nigeria | *Ae. aegypti, Ae. albopictus* | [84] |
| 85 | Traore et al. | 2022 | Côte d’Ivoire | *Ae. aegypti* | [85] |
| 86 | Mataba et al. | 2022 | Tanzania | *Ae. aegypti* | [86] |
| 87 | Oforka et al. | 2022 | Nigeria | *Ae. aegypti* | [87] |
| 88 | Obi et al. | 2022 | Nigeria | *Ae. vittatus* | [88] |

**References**

1.	Hougard J, Duval J, Escaffre H: **Field evaluation of larvicidal activity of a water-dispersible concentrate of Bacillus thuringiensis H-14 in a yellow fever focus in Ivory Coast.** *Cahiers ORSTOM, Serie Entomologie Medicale et Parasitologie* 1985, **23**.

2.	Merlin M, Josse R, Kouka-Bemba D, Meunier D, Senga J, Simonkovich E, Malonga JR, Manoukou F, Georges AJ: **[Evaluation of immunological and entomotological indices of yellow fever in Pointe-Noire, People's Republic of Congo].** *Bull Soc Pathol Exot Filiales* 1986, **79:**199-206.

3.	Zeze DG, Koffi AA: **[Stegomyian indices and epidemiological status of yellow fever in a rural area of the Ivory Coast].** *Med Trop (Mars)* 1994, **54:**324-330.

4.	Rodier GR, Gubler DJ, Cope SE, Cropp CB, Soliman AK, Polycarpe D, Abdourhaman MA, Parra JP, Maslin J, Arthur RR: **Epidemic dengue 2 in the city of Djibouti 1991-1992.** *Trans R Soc Trop Med Hyg* 1996, **90:**237-240.

5.	Anyanwu G, Iwuala M: **Mosquito breeding sites: distribution and relative abundance of species in the Jos Plateau, Nigeria.** *Medical Entomology and Zoology* 1999.

6.	Diarrassouba S, Herve J, Dossou-Yovo J, Carnevale P: **Water stocking habits and seasonal variations in biting rates of Aedes (Stegomyia) aegypti Linnaeus (Diptera: Culicidae) in a sub-sudanian village of Cote d'Ivoire.** *Research and reviews in parasitology* 2000.

7.	Fontenille D, Toto JC: **Aedes (Stegomyia) albopictus (Skuse), a potential new Dengue vector in southern Cameroon.** *Emerg Infect Dis* 2001, **7:**1066-1067.

8.	Akoua-Koffi C, Diarrassouba S, Bénié VB, Ngbichi JM, Bozoua T, Bosson A, Akran V, Carnevale P, Ehouman A: **[Investigation surrounding a fatal case of yellow fever in Côte d'Ivoire in 1999].** *Bull Soc Pathol Exot* 2001, **94:**227-230.

9.	Simard F, Nchoutpouen E, Toto JC, Fontenille D: **Geographic distribution and breeding site preference of Aedes albopictus and Aedes aegypti (Diptera: culicidae) in Cameroon, Central Africa.** *J Med Entomol* 2005, **42:**726-731.

10.	Mwangangi J, Shililu J, Muturi E, Gu W, Mbogo C, Kabiru E, Jacob B, Githure J, Novak R: **Dynamics of immature stages of Anopheles arabiensis and other mosquito species (Diptera: Culicidae) in relation to rice cropping in a rice agro-ecosystem in Kenya.** *J Vector Ecol* 2006, **31:**245-251.

11.	Midega JT, Nzovu J, Kahindi S, Sang RC, Mbogo C: **Application of the pupal/demographic-survey methodology to identify the key container habitats of Aedes aegypti (L.) in Malindi district, Kenya.** *Ann Trop Med Parasitol* 2006, **100 Suppl 1:**S61-S72.

12.	Olayemi I, Ande A: **Species composition and larval habitats of mosquitoes (Diptera: Culicidae) in Ilorin, Nigeria.** *Environmental Science, Biology* 2008.

13.	Dehecq J-S, Fohr G, Thiria J: **Plan de lutte contre Aedes albopictus pendant l’épidémie de chikungunyaà La Réunion en 2005-2007.** Bulletin Epidemiologique Hebdomadaire2008.

14.	Delatte H, Dehecq JS, Thiria J, Domerg C, Paupy C, Fontenille D: **Geographic distribution and developmental sites of Aedes albopictus (Diptera: Culicidae) during a Chikungunya epidemic event.** *Vector Borne Zoonotic Dis* 2008, **8:**25-34.

15.	Sang RC, Ahmed O, Faye O, Kelly CL, Yahaya AA, Mmadi I, Toilibou A, Sergon K, Brown J, Agata N, et al: **Entomologic investigations of a chikungunya virus epidemic in the Union of the Comoros, 2005.** *Am J Trop Med Hyg* 2008, **78:**77-82.

16.	Tun-Lin W, Lenhart A, Nam VS, Rebollar-Téllez E, Morrison AC, Barbazan P, Cote M, Midega J, Sanchez F, Manrique-Saide P, et al: **Reducing costs and operational constraints of dengue vector control by targeting productive breeding places: a multi-country non-inferiority cluster randomized trial.** *Trop Med Int Health* 2009, **14:**1143-1153.

17.	Kamgang B, Happi JY, Boisier P, Njiokou F, Hervé JP, Simard F, Paupy C: **Geographic and ecological distribution of the dengue and chikungunya virus vectors Aedes aegypti and Aedes albopictus in three major Cameroonian towns.** *Med Vet Entomol* 2010, **24:**132-141.

18.	Yaro S, Zango A, Rouamba J, Diabaté A, Dabiré R, Kambiré C, Tiendrebeogo SM, Yonli T, Ouango JG, Diagbouga SP: **[Epidemiological situation of yellow fever in Burkina Faso from 2003 to 2008].** *Bull Soc Pathol Exot* 2010, **103:**44-47.

19.	Konan YL, Fofana D, Coulibaly ZI, Diallo A, Koné AB, Doannio JM, Ekra KD, Odéhouri-Koudou P: **[Entomological investigations conducted around ten cases of yellow fever in 2009 in the Denguélé sanitary region, Côte-d'Ivoire].** *Bull Soc Pathol Exot* 2011, **104:**296-302.

20.	Diallo D, Diagne CT, Hanley KA, Sall AA, Buenemann M, Ba Y, Dia I, Weaver SC, Diallo M: **Larval ecology of mosquitoes in sylvatic arbovirus foci in southeastern Senegal.** *Parasit Vectors* 2012, **5:**286.

21.	Seidahmed OM, Siam HA, Soghaier MA, Abubakr M, Osman HA, Abd Elrhman LS, Elmagbol B, Velayudhan R: **Dengue vector control and surveillance during a major outbreak in a coastal Red Sea area in Sudan.** *East Mediterr Health J* 2012, **18:**1217-1224.

22.	Duarte E, Pereira J, Oliveira H, Lima H, Perez A, Pile E: **Aedes (Stegomyia) aegypti (Diptera: Culicidae) in some islands of Cape Verde: types of breeding sites and their relationship with larval presence.** *Arq Inst Biol* 2013.

23.	Diallo M, Tall A, Dia I, Ba Y, Sarr FD, Ly A, Faye J, Badiane A, Diakhaby G, Faye P, et al: **Yellow fever outbreak in central part of Senegal 2002: epidemiological findings.** *Journal of Public Health and Epidemiology* 2013, **5-7:**291-296.

24.	Kone AB, Konan YL, Coulibaly ZI, Fofana D, Guindo-Coulibaly N, Diallo M, Doannio JM, Ekra KD, Odehouri-Koudou P: **[Entomological evaluation of the risk of urban outbreak of yellow fever in 2008 in Abidjan, Côte d'Ivoire].** *Med Sante Trop* 2013, **23:**66-71.

25.	Kamgang B, Ngoagouni C, Manirakiza A, Nakouné E, Paupy C, Kazanji M: **Temporal patterns of abundance of Aedes aegypti and Aedes albopictus (Diptera: Culicidae) and mitochondrial DNA analysis of Ae. albopictus in the Central African Republic.** *PLoS Negl Trop Dis* 2013, **7:**e2590.

26.	Idowu O, Adeleke M, Oriaku E: **Assessment of natural and artificial breeding sites of mosquitoes in rural and urban areas of Abeokuta, Ogun State, Nigeria.** *Munis Entomology and Zoology* 2014.

27.	Noden BH, Musuuo M, Aku-Akai L, van der Colf B, Chipare I, Wilkinson R: **Risk assessment of flavivirus transmission in Namibia.** *Acta Trop* 2014, **137:**123-129.

28.	Mint Lekweiry K, Ould Ahmedou Salem MS, Ould Brahim K, Ould Lemrabott MA, Brengues C, Faye O, Simard F, Ould Mohamed Salem Boukhary A: **Aedes aegypti (Diptera: Culicidae) in Mauritania: First Report on the Presence of the Arbovirus Mosquito Vector in Nouakchott.** *J Med Entomol* 2015, **52:**730-733.

29.	Getachew D, Tekie H, Gebre-Michael T, Balkew M, Mesfin A: **Breeding Sites of Aedes aegypti: Potential Dengue Vectors in Dire Dawa, East Ethiopia.** *Interdiscip Perspect Infect Dis* 2015, **2015:**706276.

30.	Yalwala S, Clark J, Oullo D, Ngonga D, Abuom D, Wanja E, Bast J: **Comparative efficacy of existing surveillance tools for Aedes aegypti in Western Kenya.** *J Vector Ecol* 2015, **40:**301-307.

31.	Higa Y, Abílio AP, Futami K, Lázaro MA, Minakawa N, Gudo ES: **Abundant Aedes (Stegomyia) aegypti aegypti mosquitoes in the 2014 dengue outbreak area of Mozambique.** *Trop Med Health* 2015, **43:**107-109.

32.	Lutomiah J, Barrera R, Makio A, Mutisya J, Koka H, Owaka S, Koskei E, Nyunja A, Eyase F, Coldren R, Sang R: **Dengue Outbreak in Mombasa City, Kenya, 2013-2014: Entomologic Investigations.** *PLoS Negl Trop Dis* 2016, **10:**e0004981.

33.	Mboera LE, Mweya CN, Rumisha SF, Tungu PK, Stanley G, Makange MR, Misinzo G, De Nardo P, Vairo F, Oriyo NM: **The Risk of Dengue Virus Transmission in Dar es Salaam, Tanzania during an Epidemic Period of 2014.** *PLoS Negl Trop Dis* 2016, **10:**e0004313.

34.	Usman A, Ball JD, Rojas DP, Berhane A, Ghebrat Y, Mebrahtu G, Gebresellasie A, Zehaie A, Mufunda J, Liseth O, et al: **Dengue fever outbreaks in Eritrea, 2005-2015: A case for strengthening surveillance, control and reporting.** *Glob Health Res Policy* 2016, **1:**17.

35.	Ngugi HN, Mutuku FM, Ndenga BA, Musunzaji PS, Mbakaya JO, Aswani P, Irungu LW, Mukoko D, Vulule J, Kitron U, LaBeaud AD: **Characterization and productivity profiles of Aedes aegypti (L.) breeding habitats across rural and urban landscapes in western and coastal Kenya.** *Parasit Vectors* 2017, **10:**331.

36.	Kamgang B, Yougang AP, Tchoupo M, Riveron JM, Wondji C: **Temporal distribution and insecticide resistance profile of two major arbovirus vectors Aedes aegypti and Aedes albopictus in Yaoundé, the capital city of Cameroon.** *Parasit Vectors* 2017, **10:**469.

37.	Mathias L, Baraka V, Philbert A, Innocent E, Francis F, Nkwengulila G, Kweka EJ: **Habitat productivity and pyrethroid susceptibility status of Aedes aegypti mosquitoes in Dar es Salaam, Tanzania.** *Infect Dis Poverty* 2017, **6:**102.

38.	Mahgoub MM, Kweka EJ, Himeidan YE: **Characterisation of larval habitats, species composition and factors associated with the seasonal abundance of mosquito fauna in Gezira, Sudan.** *Infect Dis Poverty* 2017, **6:**23.

39.	Zahouli JBZ, Koudou BG, Müller P, Malone D, Tano Y, Utzinger J: **Urbanization is a main driver for the larval ecology of Aedes mosquitoes in arbovirus-endemic settings in south-eastern Côte d'Ivoire.** *PLoS Negl Trop Dis* 2017, **11:**e0005751.

40.	Zahouli JBZ, Koudou BG, Müller P, Malone D, Tano Y, Utzinger J: **Effect of land-use changes on the abundance, distribution, and host-seeking behavior of Aedes arbovirus vectors in oil palm-dominated landscapes, southeastern Côte d'Ivoire.** *PLoS One* 2017, **12:**e0189082.

41.	Kone B, Konan L, Fofana D, Koffi A, Coulibaly D, Benie J: **Entomological evaluation of the risk of spread ofthe dengue 3 epidemic in the health district ofCocody (Abidjan, Côte d’Ivoire).** *International Journal of Mosquito Research* 2018, **5 (5):**19-24.

42.	Konongoi SL, Nyunja A, Ofula V, Owaka S, Koka H, Koskei E, Eyase F, Langat D, Mancuso J, Lutomiah J, Sang R: **Human and entomologic investigations of chikungunya outbreak in Mandera, Northeastern Kenya, 2016.** *PLoS One* 2018, **13:**e0205058.

43.	Bakr A, Bashir N, Kehail M: **Spatio-temporal distribution of Aedes mosquitoes (Diptera: Culicidae), in Alfasher Locality North Darfur State, Sudan.** *International Journal of Mosquito Research* 2018.

44.	Abílio AP, Abudasse G, Kampango A, Candrinho B, Sitoi S, Luciano J, Tembisse D, Sibindy S, de Almeida APG, Garcia GA, et al: **Distribution and breeding sites of Aedes aegypti and Aedes albopictus in 32 urban/peri-urban districts of Mozambique: implication for assessing the risk of arbovirus outbreaks.** *PLoS Negl Trop Dis* 2018, **12:**e0006692.

45.	Mugabe VA, Ali S, Chelene I, Monteiro VO, Guiliche O, Muianga AF, Mula F, António V, Chongo I, Oludele J, et al: **Evidence for chikungunya and dengue transmission in Quelimane, Mozambique: Results from an investigation of a potential outbreak of chikungunya virus.** *PLoS One* 2018, **13:**e0192110.

46.	Kamgang B, Wilson-Bahun TA, Irving H, Kusimo MO, Lenga A, Wondji CS: **Geographical distribution of.** *Wellcome Open Res* 2018, **3:**79.

47.	Saleh F, Kitau J, Konradsen F, Alifrangis M, Lin CH, Juma S, Mchenga SS, Saadaty T, Schiøler KL: **Habitat Characteristics For Immature Stages of.** *J Am Mosq Control Assoc* 2018, **34:**190-200.

48.	Aikpon R, Klotoe J, Dramane G, Brettenny M, Lawani Y: **Larval breeding characteristics and distribution of Aedes mosquito species in the economic capital of Benin: A public health concern.** *International Journal of Entomology Research* 2019.

49.	Ouattara LPE, Sangaré I, Namountougou M, Hien A, Ouari A, Soma DD, Kassié D, Diabaté A, Gnankiné O, Bonnet E, et al: **Surveys of Arboviruses Vectors in Four Cities Stretching Along a Railway Transect of Burkina Faso: Risk Transmission and Insecticide Susceptibility Status of Potential Vectors.** *Front Vet Sci* 2019, **6:**140.

50.	Eshag O, Bashir N, Dukeen M: **Prevalence, habitat and productivity profiles of*Aedes* mosquitoes (Diptera: Culicidae) in Sennarstate, Sudan.** 2019, **6**.

51.	Mulchandani R, Massebo F, Bocho F, Jeffries CL, Walker T, Messenger LA: **A community-level investigation following a yellow fever virus outbreak in South Omo Zone, South-West Ethiopia.** *PeerJ* 2019, **7:**e6466.

52.	Nebinur M, Bashir N, Dukeen M: **Presence, characterization and productivity profiles of Aedes mosquitoes (Diptera: Culicidae), in Barentu, Gash Barka Zone, Eritrea.** *International Journal of Mosquito Research* 2019.

53.	Tedjou AN, Kamgang B, Yougang AP, Njiokou F, Wondji CS: **Update on the geographical distribution and prevalence of Aedes aegypti and Aedes albopictus (Diptera: Culicidae), two major arbovirus vectors in Cameroon.** *PLoS Negl Trop Dis* 2019, **13:**e0007137.

54.	Kahamba NF, Limwagu AJ, Mapua SA, Msugupakulya BJ, Msaky DS, Kaindoa EW, Ngowo HS, Okumu FO: **Habitat characteristics and insecticide susceptibility of Aedes aegypti in the Ifakara area, south-eastern Tanzania.** *Parasit Vectors* 2020, **13:**53.

55.	Saleh F, Kitau J, Konradsen F, Kampango A, Abassi R, Schiøler KL: **Epidemic risk of arboviral diseases: Determining the habitats, spatial-temporal distribution, and abundance of immature Aedes aegypti in the Urban and Rural areas of Zanzibar, Tanzania.** *PLoS Negl Trop Dis* 2020, **14:**e0008949.

56.	Bonnet E, Fournet F, Benmarhnia T, Ouedraogo S, Dabiré R, Ridde V: **Impact of a community-based intervention on Aedes aegypti and its spatial distribution in Ouagadougou, Burkina Faso.** *Infect Dis Poverty* 2020, **9:**61.

57.	Forsyth JE, Mutuku FM, Kibe L, Mwashee L, Bongo J, Egemba C, Ardoin NM, LaBeaud AD: **Source reduction with a purpose: Mosquito ecology and community perspectives offer insights for improving household mosquito management in coastal Kenya.** *PLoS Negl Trop Dis* 2020, **14:**e0008239.

58.	Leal SDV, Fernandes Varela IB, Lopes Gonçalves AAL, Sousa Monteiro DD, Ramos de Sousa CM, Lima Mendonça MDL, De Pina AJ, Alves MJ, Osório HC: **Abundance and Updated Distribution of.** *Int J Environ Res Public Health* 2020, **17**.

59.	Wilson-Bahun TA, Kamgang B, Lenga A, Wondji CS: **Larval ecology and infestation indices of two major arbovirus vectors, Aedes aegypti and Aedes albopictus (Diptera: Culicidae), in Brazzaville, the capital city of the Republic of the Congo.** *Parasit Vectors* 2020, **13:**492.

60.	Hondt O, Akono P, Hiol J, Edou D, Tonga C, Dadji G, Kekeunou S: **Competitive adaptation of Aedes albopictus, Skuse 1894 in the presence of Aedes aegypti Linne 1862 in temporary larvae breeding sites and in the context of pyrethroids resistance in Douala (Cameroon).** *Bulletin de la Societe de Pathologie Exotique* 2020, **113**.

61.	Diouf B, Gaye A, Diagne CT, Diallo M, Diallo D: **Zika virus in southeastern Senegal: survival of the vectors and the virus during the dry season.** *BMC Infect Dis* 2020, **20:**371.

62.	Kudom AA: **Entomological surveillance to assess potential outbreak of Aedes-borne arboviruses and insecticide resistance status of Aedes aegypti from Cape Coast, Ghana.** *Acta Trop* 2020, **202:**105257.

63.	Mathias E, O'Woma O, Chidiebere U, Igwe O: **Habitat preference of mosquito larvae in Michael Okpara University of agriculture, Umudike, Nigeria.** *International Journal of Mosquito Research* 2020.

64.	Kampango A, Furu P, Sarath DL, Haji KA, Konradsen F, Schiøler KL, Alifrangis M, Saleh F, Weldon CW: **Risk factors for occurrence and abundance of Aedes aegypti and Aedes bromeliae at hotel compounds in Zanzibar.** *Parasit Vectors* 2021, **14:**544.

65.	Waldetensai A, Gemechu F, Kinfe E, Amare H, Hagos S, Teshome D, Hailemariam A, Nigatu W, Tadesse A, Keneni D, et al: **Aedes mosquito responses to control interventionsagainst the Chikungunya outbreak of Dire Dawa, Eastern Ethiopia.** *International Journal of Tropical Insect Science* 2021.

66.	Wat'senga Tezzo F, Fasine S, Manzambi Zola E, Marquetti MDC, Binene Mbuka G, Ilombe G, Mundeke Takasongo R, Smitz N, Bisset JA, Van Bortel W, Vanlerberghe V: **High Aedes spp. larval indices in Kinshasa, Democratic Republic of Congo.** *Parasit Vectors* 2021, **14:**92.

67.	Yenus M, Brhane M, Berhe M, Kiflemariam A, Sereke K: **Abundance, characterization and productivity profiles of Aedes mosquitoes (Diptera: Culicidae), in Keren, Elabered and Hagas, Anseba zone, Eritrea.** *International Journal of Mosquito Research* 2021.

68.	Gutu MA, Bekele A, Seid Y, Mohammed Y, Gemechu F, Woyessa AB, Tayachew A, Dugasa Y, Gizachew L, Idosa M, et al: **Another dengue fever outbreak in Eastern Ethiopia-An emerging public health threat.** *PLoS Negl Trop Dis* 2021, **15:**e0008992.

69.	Hamid H, Musa H, Ahmed A, Abdul Azeez T, Adam A, Abdel Malik M, El Tahir A, Kafy H, Azrag R: **Stegomyia indices of Aedes aquatic stages in El Geneina town, Western Sudan.** *East Mediterr Health J* 2021, **27:**1189-1196.

70.	Kampango A, Furu P, Sarath DL, Haji KA, Konradsen F, Schiøler KL, Alifrangis M, Weldon CW, Saleh F: **Targeted elimination of species-rich larval habitats can rapidly collapse arbovirus vector mosquito populations at hotel compounds in Zanzibar.** *Med Vet Entomol* 2021, **35:**523-533.

71.	Irikannu K, Onyido A, Umeanaeto P, Onyebueke A, Nzeukwu C, Ogbonna C, Ezeagwuna D, Ogaraku J, Asogwa K: **Breeding ecology and physicochemical properties of mosquito breeding sites in Awka South Local Government Area, Anambra State, Nigeria.** *Trends in Entomology* 2021.

72.	Ouattara A, Kouame K, Adjobe C, Koudou B: **Hotspots of Aedes aegypti (Diptera: Culicidae) larvae in Abidjan, southern Cote d'Ivoire.** *International Journal of Mosquito Research* 2021.

73.	Mbanzulu KM, Wumba R, Mboera LEG, Kayembe JN, Engbu D, Bojabwa MM, Zanga JK, Misinzo G, Kimera SI: **Pattern of.** *Trop Med Infect Dis* 2022, **7**.

74.	Mekuriaw W, Kinde S, Kindu B, Mulualem Y, Hailu G, Gebresilassie A, Sisay C, Bekele F, Amare H, Wossen M, et al: **Epidemiological, Entomological, and Climatological Investigation of the 2019 Dengue Fever Outbreak in Gewane District, Afar Region, North-East Ethiopia.** *Insects* 2022, **13**.

75.	Ngingo BL, Mboera LEG, Chengula A, Machelle I, Makange MR, Msolla M, Mwanyika GO, Rugarabamu S, Misinzo G: **Aedes aegypti abundance, larval indices and risk for dengue virus transmission in Kinondoni district, Tanzania.** *Trop Med Health* 2022, **50:**1.

76.	Yao K, Konan Y, Coulibaly Z, Silue G, Coulibaly D, N'Goran K: **Larval ecology of Aedes mosquitoes and risk of arbovirus transmission at the port of San-pedro (Cote d'Ivoire).** *International Journal of Mosquito Research* 2022.

77.	Diakaridia F, Konan L, Kone B, Coulibaly D, Beugre J, Akaffou M, Diallo A, Benie B, Dosso M, N'Goran N: **Household water storage containers: Aedes aegypti larval breeding site and high risk of transmission of arboviruses in Abidjan, Cote d'Ivoire.** *International Journal of Biological and Chemical Sciences* 2022.

78.	Guindo-Coulibaly N, Kpan MDS, Adja AM, Kouadio AMN, Assouho KF, Zoh DD, Azongnibo KRM, Remoue F, Fournet F: **Seasonal variation and intra urban heterogeneity of the entomological risk of transmission of dengue and yellow fever in Abidjan, Côte d'Ivoire.** *Med Vet Entomol* 2022, **36:**329-337.

79.	Badolo A, Sombié A, Yaméogo F, Wangrawa DW, Sanon A, Pignatelli PM, Viana M, Kanuka H, Weetman D, McCall PJ: **First comprehensive analysis of Aedes aegypti bionomics during an arbovirus outbreak in west Africa: Dengue in Ouagadougou, Burkina Faso, 2016-2017.** *PLoS Negl Trop Dis* 2022, **16:**e0010059.

80.	Ouédraogo WM, Toé KH, Sombié A, Viana M, Bougouma C, Sanon A, Weetman D, McCall PJ, Kanuka H, Badolo A: **Impact of physicochemical parameters of Aedes aegypti breeding habitats on mosquito productivity and the size of emerged adult mosquitoes in Ouagadougou City, Burkina Faso.** *Parasit Vectors* 2022, **15:**478.

81.	Owusu-Asenso CM, Mingle JAA, Weetman D, Afrane YA: **Spatiotemporal distribution and insecticide resistance status of Aedes aegypti in Ghana.** *Parasit Vectors* 2022, **15:**61.

82.	Yougang AP, Keumeni CR, Wilson-Bahun TA, Tedjou AN, Njiokou F, Wondji C, Kamgang B: **Spatial distribution and insecticide resistance profile of Aedes aegypti and Aedes albopictus in Douala, the most important city of Cameroon.** *PLoS One* 2022, **17:**e0278779.

83.	Silue G, Konan Y, Yao-Acapovi G, N'Tamon N, Yao K, Coulibaly Z, N'Goran K: **Seasonal variation of mosquito breeding sites in the industrial areas of the city of Abidjan, south-eastern Cote d'Ivoire.** *International Journal of Mosquito Research* 2022.

84.	Eze S, Aribodor D, Nwangwu U, Ogbuefi E: **Breeding ecology of mosquito species in Umunze community, Orumba south local government area, Anambra state, Nigeria.** *International Journal of Entomology Research* 2022.

85.	Traore I, Coulibaly ZI, Allali KB, Tangena JA, Konan YL, Yapi A, Dosso M: **Mosquito (Diptera: Culicidae) Larval Ecology in Rubber Plantations and Rural Villages in Dabou (Côte d'Ivoire).** *Ecohealth* 2022, **19:**259-272.

86.	Mataba GR, Munishi L, Brendonck L, Vanschoenwinkel B: **The role of anthropogenic container habitats as mosquito oviposition habitats in rural settlements in northern Tanzania.** *J Vector Ecol* 2022, **47:**69-80.

87.	Oforka C, Adeleke M: **Dry season larval habitats of mosquito vectors (Culicidae) in Ajeromi-Ifelodun area of Lagos, Nigeria.** *Munis Entomology and Zoology* 2022.

88.	Obi OA, Adebote DA, Nock IH, Josiah JG: **Ecology of.** *J Vector Borne Dis* 2022, **59:**265-274.
